# Fortuitous Encounters between Seagliders and Adult Female Northern Fur Seals (*Callorhinus ursinus*) off the Washington (USA) Coast: Upper Ocean Variability and Links to Top Predator Behavior

**DOI:** 10.1371/journal.pone.0101268

**Published:** 2014-08-25

**Authors:** Noel A. Pelland, Jeremy T. Sterling, Mary-Anne Lea, Nicholas A. Bond, Rolf R. Ream, Craig M. Lee, Charles C. Eriksen

**Affiliations:** 1 School of Oceanography, University of Washington, Seattle, Washington, United States of America; 2 National Marine Mammal Laboratory, National Oceanographic and Atmospheric Administration, Seattle, Washington, United States of America; 3 Institute for Marine and Antarctic Studies, University of Tasmania, Hobart, Tasmania, Australia; 4 Joint Institute for the Study of the Atmosphere and Ocean, Seattle, Washington, United States of America; 5 Applied Physics Laboratory, University of Washington, Seattle, Washington, United States of America; Texas A&M University-Corpus Christi, United States of America

## Abstract

Behavioral responses by top marine predators to oceanographic features such as eddies, river plumes, storms, and coastal topography suggest that biophysical interactions in these zones affect predators' prey, foraging behaviors, and potentially fitness. However, examining these pathways is challenged by the obstacles inherent in obtaining simultaneous observations of surface and subsurface environmental fields and predator behavior. In this study, migratory movements and, in some cases, diving behavior of 40 adult female northern fur seals (NFS; *Callorhinus ursinus*) were quantified across their range and compared to remotely-sensed environmental data in the Gulf of Alaska and California Current ecosystems, with a particular focus off the coast of Washington State (USA) – a known foraging ground for adult female NFS and where autonomous glider sampling allowed opportunistic comparison of seal behavior to subsurface biophysical measurements. The results show that in these ecosystems, adult female habitat utilization was concentrated near prominent coastal topographic, riverine, or inlet features and within 200 km of the continental shelf break. Seal dive depths, in most ecosystems, were moderated by surface light level (solar or lunar), mirroring known behaviors of diel vertically-migrating prey. However, seal dives differed in the California Current ecosystem due to a shift to more daytime diving concentrated at or below the surface mixed layer base. Seal movement models indicate behavioral responses to season, ecosystem, and surface wind speeds; individuals also responded to mesoscale eddies, jets, and the Columbia River plume. Foraging within small scale surface features is consistent with utilization of the inner coastal transition zone and habitats near coastal capes, which are known eddy and filament generation sites. These results contribute to our knowledge of NFS migratory patterns by demonstrating surface and subsurface behavioral responses to a spatially and temporally dynamic ocean environment, thus reflecting its influence on associated NFS prey species.

## Introduction

Northern fur seal (NFS; *Callorhinus ursinus*) migration and overwinter foraging represents a critical portion of its annual life cycle. For the Eastern Stock (animals breeding on the Pribilof Islands and Bogoslof Island, Alaska, USA) this migration begins at the onset of subarctic fall as most animals leave the breeding grounds in the Bering Sea for pelagic overwinter habitat at more southerly latitudes, remaining away from land and foraging on the open sea for the following ∼8 months. During the migration, foraging success is of fundamental importance to pregnant females, who must invest energy in their fetus and improve their own physical condition prior to the return trip to the breeding grounds in the summer [1]–[5]. Reproductive females concentrate their overwintering activity mainly in the productive coastal transition zones of the Gulf of Alaska (GA) and California Current (CC) ecosystems, though some make their way to the mid-ocean Transition Zone Chlorophyll Front [1], [6].

Within the CC and GA ecosystems and along migratory pathways to and from the breeding grounds, environmental variability that affects the abundance, distribution, availability, and quality of prey (biotic factors) and/or NFS metabolism or swimming energetics (abiotic factors) could potentially have important consequences for overwinter survival and reproductive success of adult female NFS. Since female recruitment to breeding age and annual adult female survivorship are two of the most important determinants of age structure and long-term stability in the population [3], [7]–[11], environmental variability affecting overwintering females could thus potentially exert significant influence on demography and population trends in the Eastern Stock as a whole. However, despite this potential importance, the pathways by which changes in ocean surface patterns influence foraging opportunities and success of individual adult female NFS outside of the Bering Sea, and how this is reflected and expressed in patterns of their horizontal movement, diving frequency, and vertical localization in the marine environment, are not fully understood. This lack of understanding hinders the effort to explain population declines since the 1980s within the Eastern Stock, which may be due to changes in Pacific Ocean climate, human-related causes, predation, or interactions between these factors [3], [12]–[15]. These declines are isolated to the Pribilof Islands (St. Paul and St. George Islands), collectively the largest NFS breeding sites [3], [9], [11], [16], [17]. In contrast to trends at the Pribilof Islands, pup production on other breeding islands in the eastern North Pacific Ocean (NP) and Bering Sea has been stable or increasing since the 1980s. The Bogoslof Island population has experienced exponential growth, in part due to immigration, though this flux is of insufficient magnitude to solely account for the Pribilof Islands population decline [18]–[20]. Pup production on San Miguel Island, California (USA), the largest breeding island in the California Stock, has also increased over a similar time period, though with large interannual fluctuations that are mostly explained by El Niño-Southern Oscillation (ENSO) events [21], [22].

Studies undertaken during and immediately prior to the decline of the Pribilof Islands population raised important questions surrounding the role that large and small scale environmental oceanic variability plays in influencing fitness and survivorship during the pelagic phase for juvenile and adult female NFS [3], [9], [14], [23]. At the large scales, basin-wide patterns of hydrography and marine ecosystems in the NP could play a role in the location of overwintering habitat of the NFS population and its segregation by sex and age class. It is commonly assumed that the extreme size dimorphism observed in NFS means that larger adult males are physiologically capable of remaining in the central NP, GA, or southern Bering Sea during the overwintering period, exploiting prey fields that are in colder water and located deeper in the water column [24]–[26]. The smaller females, juveniles, and pups cannot dive as deeply and may contend with greater mass-specific body heat loss in cold water (<2°C; [27]). While the thermal tolerance of juvenile and adult females allows them to exploit a wide range of winter habitats in the NP, they are likely unable to remain in the southern Bering Sea; this is especially true for pups, whose thermoregulatory abilities are not fully developed [27], [28]. For these components of the population it is assumed that overwintering habitat is more suitable off the west coast of North America where they benefit from the productive CC and GA boundary current ecosystems [29]. Here, energy is transferred from lower trophic levels to pelagic schooling fishes and squids that comprise the bulk of NFS prey [1], [30]–[32]. The idea that basin-scale patterns of surface ocean biophysical conditions, and interannual perturbations to these patterns, are important to the NFS Eastern Stock is supported by the fact that they exert a strong influence on San Miguel Island NFS and other pinniped species in the CC, where many females and pups from the Eastern Stock overwinter. For example, strong ENSO events have significantly affected NFS pup, juvenile, and adult survivorship at San Miguel Island [22] and both the abundance and feeding habits of another Eastern Pacific otariid, the California sea lion (*Zalophus californianus*; [33], [34]). Warm ENSO events cause elevated sea level height, sea surface temperatures, and a deepening of the mixed-layer depth (MLD), thermocline and nutricline that results in reduced ocean productivity and abundance and availability of pinniped prey. Along the South American west coast, ENSO also affects pup production of Galapagos fur seals (*Arctocephalus galapagoensis*) and sea lions (*Z. wollebaeki*; [35], [36]). Furthermore, periods of growth and decline in monk seal (*Monachus schauinslandi*) abundance are associated with positive and negative phases of the Pacific Decadal Oscillation – a low-frequency pattern of basin-wide ocean-atmosphere variability in the NP [37], [38].

Embedded within these broad patterns are smaller-scale or higher-frequency environmental features, such as storms, ocean eddies, or bathymetric features, to which individuals or groups of NFS have demonstrated behavioral responses. Satellite tagging of NFS during the summer breeding period and winter migration has revealed that eddy edges and surface fronts can act as a movement and/or foraging cue for adult females, males, and juvenile males [1], [24], [39], [40]. These behavioral responses have also been detected for other pinnipeds and seabirds [41]–[46]. Once out of the Bering Sea, adult female NFS are often sighted in a cross-shore band 70 to 130 km from the coast, or quoting Kajimura [31] near “sea valleys, submarine canyons, seamounts, and along the continental shelf and slope where abrupt changes…in depths and upwellings of nutrient-rich water occur.” This is presumably due to aggregations of prey in these areas. Atmospheric variability is also important during the end of the breeding season and the early fall migration. For example, pup dispersal from the Pribilof Islands in the fall tends to be abrupt and is often triggered by single storms [47]–[50]. Dispersal during transient large-scale atmospheric events or foraging near bathymetric features, eddies, or ocean fronts suggests that physical interactions in these areas are affecting behavior and potentially reproductive success of NFS [24].

The goal of this study was to investigate how variability in the ocean environment affects adult female NFS distribution, movement, and diving behavior during their overwintering phase. To do so, we examine satellite tracks for 40 Eastern Stock (St. Paul and Bogoslof Islands) seals collected in seven migratory seasons. Our approach combines remotely sensed and *in situ* environmental data to compare to seal distribution and individual behaviors at both large and small scales. At large scales, we provide a general description of the spatial distribution of females overwintering in the CC and GA and compare seal diving depths to a time series of ocean profiles taken off the coast of Washington State (WA, USA), an important overwinter foraging ground for adult females. We use statistical models to examine the effect of different environmental variables on adult female behavioral state, diving frequency, and dive depth. Furthermore, we quantify the amount of time spent in coherent mesoscale oceanographic features by ecosystem, and examine differences in habitat utilization relative to these features between behavioral modes. At small scales we combined fortuitous encounters between three individual seals and Seagliders, which are a type of long-range, profiling ocean glider [51]. These females, equipped with dive recorders, passed near profiling Seagliders, providing an opportunity to examine behavior of these individuals relative to mesoscale hydrography and subsurface structure off the WA coast. These results are supplemented by satellite measurements of sea level anomaly (SLA) and chlorophyll.

## Methods

### Ethics Statement

All work was conducted in accordance with and under the authority of the United States Marine Mammal Protection Act (National Marine Fisheries Service [NMFS] Permits 782–1455 and 782–1708). The Marine Mammal Protection Act was established in 1972 requiring all research conducted on marine mammals in the United States be done under the authority of federal permits issued by either NMFS or the United States Fish and Wildlife Service (USFWS). All applications for a permit to conduct research on marine mammals have gone through a four-stage review process that includes: 1) agency review (either NMFS or USFWS); 2) a public notice and review period; 3) review and recommendation from the Scientific Advisors to the United States Marine Mammal Commission; and 4) a final action by the reviewing agency. All capture and handling activities described in this manuscript have gone through and been approved by this process. At the time this work was conducted there was no additional requirement for review of these procedures by an institutional review board or ethics committee. In 2010, a NMFS Institutional Animal Care and Use Committee was established for the Alaska Fisheries and Northwest Fisheries Science Centers and the capture and handling protocols described here were reviewed and approved by this committee.

### Adult Female Satellite-Telemetry Data

**Figure 1:**
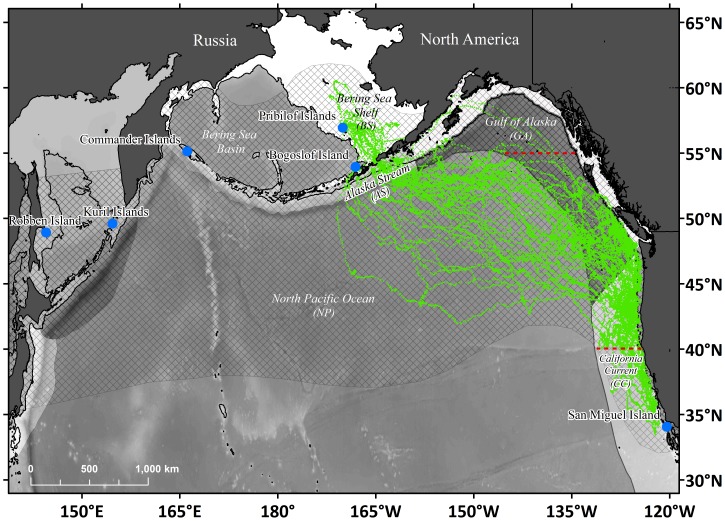
Northern fur seal habitat within the North Pacific Ocean. Blue circles indicate breeding site locations and cross-hatching corresponds to approximate range extent. *Italic* labels denote Large Marine Ecosystems (LMEs) used in this study. California Current (CC) and Gulf of Alaska (GA) LMEs are indicated by shaded polygons. Within these LMEs, shelf habitat is indicated by filled polygons adjacent to the coast. Other LMEs used in this study include the Bering Sea Shelf (BS, filled polygon within the Bering Sea), Alaska Stream (AS, light-shaded coastal habitat extending west from GA), and North Pacific Ocean (NP, all habitat seaward of the CC, GA, and AS). The Pribilof Islands (St. Paul and St. George Islands, Alaska) are collectively the largest northern fur seal breeding sites. Satellite tags were applied to 40 adult female northern fur seals on St. Paul Island and Bogoslof Island, yielding 41 satellite tracks. One animal was tracked in two separate migratory seasons. Tracking data used in this study are shown by green dots. To be considered for use, tracks were required to have entered the CC or GA LMEs and to have spent some time between 40°N–55°N (latitude boundaries indicated by dashed red lines).

Adult female NFS satellite tracking and diving data were included in the analysis if the animal migrated into the GA or CC ecosystems and spent some time between 40°N–55°N latitude (Fig. 1). It is important to note that in any NFS migratory season, many seals migrate to regions outside our ecosystem selection criteria and that our goals specifically aimed to integrate seal behavior and ecosystem processes that took place in the GA and CC ecosystems [1], [52]. To spatially define the GA and CC ecosystems we used boundaries from the National Oceanographic and Atmospheric Administration (NOAA) Large Marine Ecosystems (LMEs) of the World (http://www.lme.noaa.gov; [53]). Forty-one migratory routes from forty seals (one seal was monitored during two separate migrations) met the criteria and the dataset spanned seven migratory seasons between 2002/03 and 2009/10 (Table 1). Of the forty seals, eight were equipped with satellite transmitters that also recorded and transmitted diving behavior.

**Table 1:** Migration summary of satellite tagged adult female northern fur seals (NFS) from St. Paul and Bogoslof Islands used in this study.

Year	ID	Island	Departure date	Date of entry into NP	Date of entry into GA	Date of entry into CC	Days to arrival in the NP	Days to arrival in the GA	Days to arrival in the CC	Total tracking days
2005	283	Bogoslof	11/16/05	11/16/05	12/31/05	01/02/06	0.5	45.5	46.8	60.5
2005	285	Bogoslof	11/03/05	11/05/05	01/02/06	01/07/06	2.0	60.3	65.0	72.3
2005	291	Bogoslof	11/14/05	11/15/05	12/30/05		1.3	46.0		47.5
2005	293	Bogoslof	11/16/05	11/17/05	01/13/06	01/25/06	1.3	57.8	70.5	97.0
2005	295	Bogoslof	11/14/05	11/16/05	01/06/06	02/02/06	2.3	52.5	79.5	131.3
2005	296	Bogoslof	11/14/05	11/15/05	01/28/06	01/29/06	1.8	75.3	76.3	132.3
2005	297	Bogoslof	11/19/05	11/22/05		02/18/06	2.8		91.0	107.3
2006	450	Bogoslof	11/09/06	11/12/06	12/20/06	01/06/07	3.0	41.5	58.5	96.3
2006	456	Bogoslof	11/06/06	11/10/06	01/03/07	01/07/07	3.8	57.5	62.3	92.3
2006	460*	Bogoslof	11/17/06	11/18/06	01/04/07	01/04/07	1.3	48.0	48.3	211.5
		**Mean**	11/12	11/14	01/04	01/18	2.0	53.8	66.5	104.8
		**(SD)**	(4.9)	(4.7)	(10.7)	(16.6)	(1.0)	(10.3)	(14.5)	(46.5)
2002	189	St. Paul	11/23/02	12/04/02	02/24/03	03/12/03	11.0	93.0	108.5	240.0
2002	192	St. Paul	11/25/02	12/23/02	01/20/03	01/28/03	28.3	56.5	64.0	194.5
2002	193	St. Paul	11/23/02	12/02/02	01/22/03	01/31/03	9.3	60.5	69.0	155.0
2002	197	St. Paul	11/29/02	12/07/02	12/22/02		8.3	23.5		213.3
2002	198	St. Paul	11/26/02	11/30/02	01/16/03	01/19/03	3.8	51.0	53.8	71.0
2002	200	St. Paul	11/29/02	12/08/02	01/14/03	01/22/03	9.3	46.3	54.0	87.0
2002	201	St. Paul	11/28/02	12/05/02	02/12/03	03/26/03	6.8	76.0	117.3	195.0
2004	243	St. Paul	11/10/04	11/20/04	12/24/04		10.8	44.3		138.8
2004	246	St. Paul	11/13/04	11/19/04	01/27/05		6.0	75.3		148.3
2004	251	St. Paul	11/21/04	11/29/04	02/02/05	02/03/05	7.8	72.5	74.0	114.8
2004	254	St. Paul	11/14/04	12/05/04	01/04/05	03/23/05	21.0	51.3	129.5	198.0
2004	256	St. Paul	11/17/04	12/07/04	03/04/05		19.8	107.0		194.3
2004	257	St. Paul	11/13/04	11/20/04	12/18/04	01/02/05	6.8	35.5	50.5	111.5
2005	305	St. Paul	11/06/05	11/13/05	01/07/06		7.8	62.8		64.3
2005	317	St. Paul	11/08/05	11/14/05	12/20/05	01/24/06	6.0	42.5	77.5	82.3
2006	427	St. Paul	11/29/06	12/13/06	01/16/07	02/09/07	13.3	48.0	71.8	159.5
2006	439	St. Paul	11/12/06	11/16/06	12/20/06	12/21/06	4.8	38.0	39.5	132.8
2006	442	St. Paul	11/12/06	11/19/06	01/03/07	01/11/07	7.0	52.3	60.5	66.5
2007	626*	St. Paul	11/17/07	11/23/07	01/15/08	04/15/08	5.3	58.3	149.5	249.5
2007	627	St. Paul	11/18/07	11/23/07	01/10/08	01/16/08	5.0	53.5	59.3	81.8
2007	628*	St. Paul	11/17/07	11/23/07	12/26/07	01/07/08	6.3	39.0	51.0	217.8
2007	630	St. Paul	11/18/07	11/28/07	12/02/07	01/28/08	10.0	13.8	70.8	182.3
2008	661	St. Paul	11/21/08	11/25/08	01/09/09	01/11/09	4.0	49.3	51.3	220.5
2008	662	St. Paul	11/19/08	11/29/08	12/05/08	01/02/09	9.8	15.5	43.8	178.5
2008	663	St. Paul	11/20/08	11/27/08	12/27/08	01/05/09	7.5	37.5	46.5	153.0
2008	666	St. Paul	11/21/08	11/28/08	01/04/09	01/06/09	7.3	44.5	46.3	56.0
2008	668	St. Paul	11/21/08	11/28/08	03/18/09	03/23/09	6.8	116.8	122.0	225.0
2009	662B	St. Paul	11/11/09	11/19/09	12/30/09	01/19/10	8.0	48.8	68.8	122.0
2009	670	St. Paul	11/05/09	11/09/09	11/26/09		4.8	21.3		110.0
2009	676	St. Paul	11/12/09	11/16/09	01/05/10	01/11/10	4.0	54.5	60.3	119.0
2009	677	St. Paul	10/15/09	10/29/09	01/02/10	01/08/10	14.3	79.0	85.3	139.0
		**Mean**	11/17	11/26	01/09	01/29	9.1	53.8	73.0	149.1
		**(SD)**	(9.1)	(10.9)	(25.4)	(31.0)	(5.4)	(23.8)	(29.6)	(56.8)

Table columns indicate deployment year, seal identification number, breeding island of origin, departure date from breeding island, dates of entry into the North Pacific (NP), Gulf of Alaska (GA), and California Current (CC) Large Marine Ecosystems, travel time to these ecosystems, and total tracking days.

*Female NFS equipped with dive recorders who foraged near Seagliders.

Seal capture and satellite transmitter deployments took place on two islands, Bogoslof (n = 10 deployments; 53.94°N, 168.04°W) and St. Paul (n = 31 deployments; 57.11°N, 170.29°W) Islands, Alaska. Satellite transmitter types used included KiwiSat 101 and 202 Platform Terminal Transmitters (PTTs; Sirtrack Limited, Havelock North, New Zealand) and ST10 and ST16 Satellite Dive Recorder, SPLASH, and SPOT 5 PTTs (Wildlife Computers, Redmond, WA). All instruments were consistently programmed to transmit during two periods every 24 h. The time of day at which these periods took place varied between years. Descriptions of how each transmitter type was programmed can be found in Loughlin et al. [54] and Ream et al. [1] for the KiwiSat 101, ST10 and ST16. Lea et al. [49], [55] and Sterling et al. [25] describe instrument programming methods and dive data processing for SPLASH, SPOT 5, and KiwiSat 202 satellite transmitters. Transmitted tag information, location estimates, and in some cases, dive behavior summaries were received and processed by Service Argos (http://www.argos-system.org).

Wildlife Computers ST10, ST16 and SPLASH satellite-dive recorders were programmed to collect data in 6 h time periods and distribute dive depths among 14 pre-defined dive depth bins (2, 4, 6, 10, 20, 34, 50, 74, 100, 124, 150, 174, 200, >200 m). Dives >6 m were analyzed and the average dive depth and total number of dives for each 6 h period were used as response variables in generalized linear mixed-effects models (GLMM) of diving behavior (see Methods section *Behavior Statistical Analyses*).

### Modeling Seal Movement

Raw location data for each tag, calculated by Service Argos, were obtained at irregular time intervals within a deployment and with varying degrees of spatial error. However, for analysis purposes, it is desirable to interpolate these location data to a time base with regular intervals, such that they are aligned with the time base of environmental variables to be used as explanatory variables in GLMMs. To do so, we fitted seal Argos location data with a switching state-space model (SSSM), which estimated the evolution of each animal's position and behavior through time by modeling seal movement as a finite-difference correlated random walk process [25], [56]–[59]. Given the animal's release position, and assumptions about the distribution of turning angle and correlation between direction and move speed during transit movements [60], SSSMs use a Bayesian approach with Markov chain Monte Carlo (MCMC) estimation to fit a model to each animal track. The MCMC procedure was performed using WinBUGS and implemented with R statistical software (http://www.r-project.org; V2.14.1) and the R2WinBUGS package. The SSSMs estimate seal locations uniformly spaced in time every 6 h and allow for all observations to be compared at the same temporal scale. These models have additional advantages of accounting for the spatial error associated with Argos positions, regardless of the listed quality class of each position measurement, and providing an estimate of seal horizontal movement behavior for each 6 h period on a continuous scale from 0 (“transient,” fast, somewhat linear or directed movements) to 1 (“resident,” area-restricted search or foraging movements) [61]. Estimates of seal horizontal behavior defined in this way are hereafter referred to as “behavioral state” [25] and indicated by the mathematical symbol 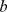 (for a glossary of mathematical symbols and acronyms used in this manuscript, refer to Table 2).

**Table 2:** Glossary of acronyms and mathematical symbols used in this manuscript.

Acronym	Definition
AIC	Akaike's Information Criterion
AVISO	Archiving, Validation, and Interpretation of Satellite Oceanographic data (source for SLA products)
BC	British Columbia, Canada
BS	Bering Sea Shelf
CC	California Current
CF	Cape Flattery, USA
Chl*a*	Chlorophyll-*a*
DESW1	National Buoy Data Center Destruction Island station
EKE	Eddy kinetic energy – see Methods section *Supplementary Environmental Data*
ENSO	El Niño-Southern Oscillation
GA	Gulf of Alaska
GAM	Generalized additive model – *see* Methods section *Statistical Analysis of Behavior Relative to Seaglider Data*
GH	Grays Harbor, USA
GLMM	Generalized linear mixed-effects model – *see* Methods section *Behavioral Statistical Analyses*
K-S	Kolmogorov-Smirnov
LME	NOAA Large Marine Ecosystem
MCMC	Markov chain Monte Carlo
MLD	Mixed-layer depth
MODIS Aqua	Moderate Resolution Imaging Spectroradiometer – Aqua (ocean color satellite)
NCEP2	National Centers for Environmental Prediction-Department of Energy Reanalysis 2 product
NFS	Northern fur seal (*Callorhinus ursinus*)
NMFS	National Marine Fisheries Service
NOAA	National Oceanographic and Atmospheric Administration
NP	North Pacific Ocean
OR	Oregon, USA
PDF	Probability density function
PTT	Platform Terminal Transmitter
SD	Standard deviation
SeaWiFS	Sea-viewing Wide Field-of-view Sensor (ocean color satellite)
SG	Seaglider
SLA	Sea level anomaly
SSSM	Switching state-space model – *see* Methods section *Modeling Seal Movement*
USFWS	United States Fish and Wildlife Service
UTC	Coordinated Universal Time
WA	Washington State, USA
WET	Western Environmental Technologies

### Estimates of Horizontal Habitat Utilization

#### Two-Dimensional, Alongshore, and Cross-Shore Utilization

Using the estimated 6 h female locations, we analyzed two-dimensional overwintering habitat utilization with kernel-smoothed estimates of adult female range in the eastern Pacific Ocean destination zone, defined as the region between 140°W–120°W and 30°N–55°N. This domain was divided into a grid with 0.1° resolution, and a bivariate normal kernel density estimate of female utilization distribution was computed at each grid point using a fixed 15 km smoothing parameter [62]. The smoothing parameter length was chosen arbitrarily in order to resolve large-scale features of the overwintering distribution while still providing adequate detail around prominent coastal features and female distribution peaks. The kernel-smoothed density grid allows calculation of the 95% utilization contour, which is the minimum area that could be drawn to encompass 95% of female habitat utilization in the destination region. This method was also used to calculate range extent for various lower utilization percentiles, which illustrate smaller-scale peaks in adult female foraging distribution. Utilization contours were computed for the full dataset as well as for a subset of 6 h locations corresponding only to area-restricted search behavior, defined as those with 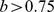 following Jonsen et al. [60].

We constructed empirical alongshore distributions for the females as they traveled through the CC and GA LMEs (Fig. 1) from January to June. Total time spent by tracked females within 0.5° latitudinal bands between 33°N to 61°N was estimated monthly. The northern boundary of this domain was extended relative to the two-dimensional utilization analysis in order to display a small amount of time spent in the northern GA ecosystem early in the overwintering period. Interannual variability was not explored, since sampling effort and tag duration varied markedly both within and between years, making it difficult to isolate real differences in foraging distribution between years. The empirical cross-shore distribution (time spent versus distance from the shelf break) of adult female distribution in 20 km bins was also computed over the entire study period. The shelf break was defined as the 200 m isobath and distances from the shelf break were computed along a great circle line perpendicular to the regional orientation of the continental shelf edge. The cross-shore distribution considered only estimated 6 h locations within a subset of the CC and GA LMEs between 41°N and 52°N. These latitudes were chosen to represent the region where the majority of female foraging occurred (see Results section *Migratory Distribution*).

#### Utilization of Mesoscale Features by State

We investigated the habitat utilization of adult female NFS relative to mesoscale eddies as identified from altimetric measurements of SLA by Chelton et al. [63]. Specifically, we explored whether the utilization distribution relative to eddies was conditioned by behavioral state. Chelton et al. [63] provide eddy estimates at 7 d time steps. For each 6 h adult female estimated location during the overwintering period, at the closest 7 d time step, the nearest mesoscale eddy center position to the female's location was identified. The distance to this eddy center, divided by the eddy's reported length scale 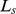 (see below in section Supplementary Environmental Data), was defined as the normalized radius 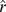. We computed kernel-smoothed probability density functions (PDFs; smoothing parameter = 0.1) for adult female utilization as a function of 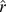 to the nearest eddy, for two categories of estimated behavioral state: area-restricted search (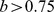) and transit (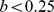). These categorical definitions follow Jonsen et al. [60]. We quantify the difference between these two distributions using measures of both distribution location and shape. As a measure of distribution location, we calculate the median 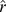 at each state, and compute the difference between these values to establish which distribution is more localized towards the center of eddy features in our observations [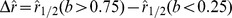, where ½ subscript indicates the median value]. For distribution shape, we calculate the difference between the probability density functions as a function of 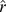, to illustrate radial positions where probability densities are higher or lower between states [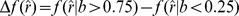, where 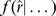 denotes the probability density at radius 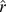, conditioned by behavioral state].

The two-sample Kolmogorov-Smirnov (K-S) test is a standard statistical test that could be used to test for differences between the area-restricted search and transit radial distributions. This test evaluates the significance of a single statistic that is sensitive to both differences in location and shape between the distributions of two sample populations. However, the high degree of autocorrelation within tracks of the satellite dataset reduces the robustness of the two-sample K-S test and introduces difficulty when computing critical test statistics based on the number of independent samples within the dataset. Instead, we test for differences between the area-restricted search and transit 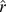 distributions using a bootstrap method. The bootstrap method is designed to measure the sensitivity of the observed differences in distribution shape and location to a random sample of individual fur seals. In other words, this test assumes that the dominant random effects in our dataset are between rather than within tracks, and explores whether between-track variance in 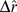 and 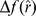 in our dataset is large compared to the observed values. In each bootstrap iteration, a random sample of 41 adult female satellite tracks was drawn with replacement from the observed 41 tracks, to create a synthetic dataset. The quantities 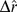 and 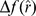 were computed for this synthetic dataset and this was repeated for 10,000 iterations. Confidence bounds for the observed values of 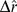 and 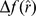 were computed from the bootstrap distributions using bias-correction and acceleration [64].

### Behavior Statistical Analyses

#### Generalized Linear Mixed-Effects Models

We followed methods detailed in Sterling et al. [25] and used GLMMs to investigate the effects of several environmental variables on three seal response variables – behavioral state, and the average dive depth and total number of dives in each 6 h dive period. In a large tracking dataset like the one employed in this study, between-animal variability in the degree and character of behavioral responses can confound the ability of ordinary linear models to detect correlations between predictor and response variables [65]. Generalized linear mixed-effects models were chosen for their flexibility in allowing us to specify individual animals as a random effect in our dataset, and for their established use in modeling behavioral responses of this and other pinniped species [25], [55], [57]. For seal behavioral state, all 41 seal migration tracks consisting of 22,597 estimated locations and behavioral states were used. We assumed an AR(1) autocorrelation structure within each track and to assist with normality, we added 0.0001 to behavioral state values of 0 and subtracted 0.0001 from behavioral state values of 1 prior to logit transforming all the behavioral state values. With respect to seal dive behavior, only 8 of the 41 female seal tracks had corresponding dive data sets. These tracks were collected in four migratory seasons (Table 1) and all eight seals traveled to the GA ecosystem, while seven of these seals entered the CC ecosystem. From these eight seals we received 1,888 dive summaries, which we then used to calculate the average dive depth and the number of dives for each 6 h dive histogram period [25], [55]. Both the average dive depth and number of dives in each 6 h period were log-transformed prior to model fitting.

Movement and dive behavior were assessed with respect to several environmental and habitat fixed effects variables. For behavioral state, these included ecosystem, season, surface wind speed, and surface ocean kinetic energy. For the average dive depth and number of dives in each 6 h period, we added the effects of light from both the sun and moon. As a proxy for sunlight, we calculated the fraction of daylight time in each 6 h interval (hereafter, “proportion daylight”) using the NOAA Sunrise/Sunset and Solar Position calculators (http://www.srrb.noaa.gov/highlights/sunrise/sunrise.html). For moonlight level, we used calculations of the lunar fractions (illuminated area divided by total area) extracted from the United States Naval Observatory website (http://aa.usno.navy.mil/data/docs/MoonFraction.php). To consider ecosystem-specific effects, we used the same modification of NOAA's LMEs described in Sterling et al. [25] as explanatory variables. These included the Alaska Stream (AS) and Bering Sea Shelf (BS), in addition to the NP, CC and GA (Fig. 1). There were very few estimated locations within the Bering Sea Basin (n = 19 dive summaries, n = 72 behavioral state estimates) and these were excluded from the behavioral statistical analyses. Season was defined as days since 1 October.

Surface wind speeds were obtained for each 6 h seal location from the National Centers for Environmental Prediction-Department of Energy Reanalysis 2 product (hereafter, “NCEP2”). These data are distributed by the NOAA Office of Oceanic and Atmospheric Research, Earth Sciences Research Laboratory Physical Sciences Division, Boulder, Colorado (USA), and made available from their web site at http://www.esrl.noaa.gov/psd/. The NCEP2 product gives surface (10 m height) east-west (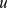) and north-south (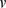) wind velocity components at four daily time steps (0000, 0600, 1200, and 1800 Coordinated Universal Time [UTC]) on a 2.5° resolution global grid. Seal location data were intentionally aligned with these time intervals using SSSMs. At each time point, 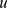 and 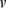 estimates from NCEP2 were interpolated from the global grid to seal estimated locations and converted to wind speed (m s^−1^) and direction. In addition to wind, previous studies have identified surface ocean mesoscale circulation as an influence on horizontal behavior [1]. We calculated eddy kinetic energy (EKE) from satellite estimates of surface geostrophic velocity anomaly (see below in section *Supplementary Environmental Data*) and evaluated this variable as a predictor of estimated behavioral state [66]. For the reasons outlined above in section *Estimates of Horizontal Habitat Utilization*, interannual variability was not explored in analyses of movement and diving behavior, which likely contributes to unexplained variance in each model.

Models were built and calculated using the nlme V3.1-103 package within the R statistical software. For all models the NP ecosystem response was used as the base or reference model. Several model configurations for each response variable were constructed using the main effects and interaction terms, fit by maximum likelihood methods, and then contrasted and ranked using Akaike's Information Criterion (AIC; [67]), where the top models were judged by lowest AIC score. The configuration of main effects and interaction terms for the top three models of each response variable are presented in Table 3. For the top-ranked model, the coefficients and significance level of each term are also presented (see Results section *Diving and Movement Behavior*).

**Table 3:** Top-ranked generalized linear mixed-effects models (GLMMs) result for number of dives per 6 h period, average maximum dive depth per 6 h period, and estimated behavioral state.

Response Variable		Model Terms	AIC	ΔAIC
**Number of Dives**				
(n = 1888)				
	(a)	DAYL+LME+SEAS	5909.3	0.0
		+(DAYL×LME)		
	(b)	DAYL+LME+LUN	5970.5	61.2
		+(DAYL×LME)+(DAYL×LUN)		
	(c)	DAYL+LME+LUN+SEAS	5970.7	61.5
		+(SEAS×DAYL)		
**Dive Depth**				
(n = 1888)				
	(a)	DAYL+LME+LUN+SEAS	2857.1	0.0
		+(DAYL×LME)+(DAYL×LUN)+(LUN×LME)		
	(b)	DAYL+LME+LUN+SEAS	2910.5	53.4
		+(DAYL×LUN)+(LUN×LME)		
	(c)	DAYL+LME+LUN	2920.1	63.0
		+(DAYL×LME)+(DAYL×LUN)+(LUN×LME)		
**Behavioral State**				
(n = 22597)				
	(a)	WIND+SEAS+LME	61974.3	0.0
		+(WIND×SEAS)		
	(b)	WIND+SEAS+LME	61979.6	5.3
		+(WIND×LME)		
	(c)	WIND+SEAS	61984.8	10.5
		+(WIND×SEAS)		

Columns indicate response variable, model terms, Akaike's Information Criterion (AIC) score, and difference in AIC score from the best model (ΔAIC). Model terms are abbreviated as follows: DAYL (proportion daylight in each 6 h period), LME (NOAA Large Marine Ecosystem), SEAS (days since 1 October), LUN (lunar fraction in each 6 h period), WIND (NCEP2 interpolated 10 m wind speed).

### Washington Coast Seaglider Sampling

**Figure 2:**
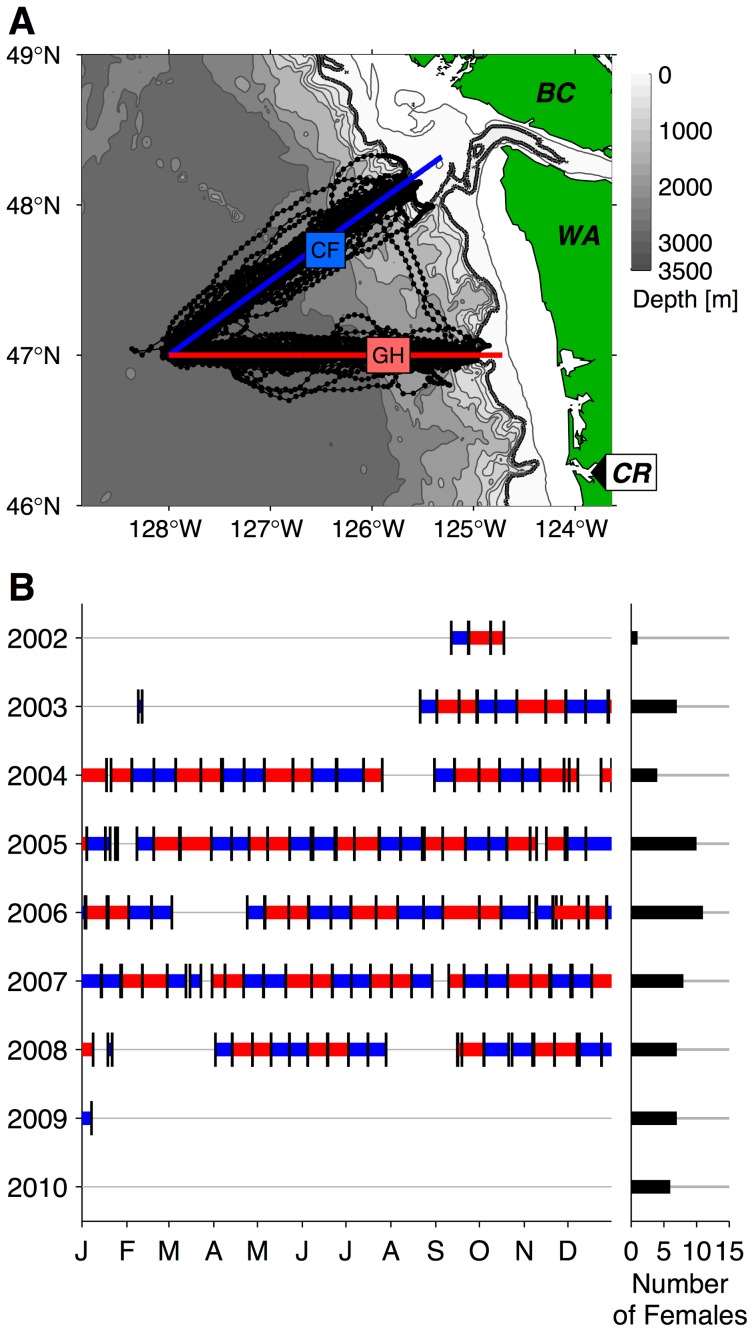
Seaglider time series over the Washington continental slope region. (A) Intended navigational track pattern for Cape Flattery (blue, labeled CF) and Grays Harbor (red, labeled GH) transects, with Seaglider tracks (black lines) and dive mid-point locations (black circles). Isobaths are contoured at 100 and 200 m, at 200 m intervals to 1000 m, and at 500 m intervals at depths greater than 1000 m (depth scale at top right). The 200 m isobath is highlighted in dark gray. Black labels denote Washington (WA), Vancouver Island, British Columbia (BC), and the mouth of the Columbia River (CR). (B) Yearly Seaglider pattern occupation and overlap with satellite-tagged northern fur seal adult female presence in the California Current and Gulf of Alaska ecosystems. The left portion of panel B shows Seaglider transit along the Cape Flattery line (blue), Grays Harbor line (red), and section boundaries (turnaround points, indicated by black vertical lines). Black bars at right show the number of individual satellite-tagged adult females present in the California Current and Gulf of Alaska ecosystems during each year.

We compared adult NFS female behavioral patterns and satellite-tracked movements to a time series of remote *in situ* hydrography collected by Seagliders operating off the WA coast in the same years and same area that adult female NFS overwintered. The Seaglider time series included data collected from two test deployments in late 2002 and early 2003 and nearly continuous data from late 2003 to early 2009 (Fig. 2; [68]–[70]). Seaglider is a long-range ocean glider, which operates by decreasing (increasing) its buoyancy in order to descend (ascend) from the surface to 1000 m [51]. It is equipped with small wings that generate lift (downforce) during the descent (ascent) phase, allowing the vehicle to move horizontally in a sawtooth pattern with an average horizontal speed through the water of approximately 0.2 m s^−1^. A dive-climb cycle to full depth lasts 8 hours and covers 6 km horizontally. Seagliders were programmed to surface and transmit profile data after every dive-climb cycle. Washington coast deployments typically lasted several months over which time a Seaglider performed several hundred of these cycles. Each Seaglider was equipped with a Paine 211-75-710-05 pressure transducer and custom-fitted SeaBird Electronics SBE-3 thermistor and SBE-4 conductivity cell mounted in a dorsal sting in order to sample temperature, salinity, and pressure. Conductivity cells and thermistors on WA coast Seagliders were calibrated by SeaBird before and after each deployment. Each vehicle also carried a Western Environmental Technologies (WET) Labs ECO-BB2F optical “puck”-style sensor, which sampled chlorophyll-*a* (Chl*a*) fluorescence and optical backscatter (proxies for phytoplankton pigment and particle concentration, respectively) in the top 150 m of each vertical profile [68].

During the 5.5-year time series, Seagliders collected hydrographic data while navigating along two cross-shore transect lines. These 210–225 km long transects extended from two points along the shelf break and were joined at their offshore end at 47°N, 128°W (Fig. 2A). A single crossing usually required two weeks of transit time and recorded 50–75 dive-climb cycles, thus yielding 100–150 vertical profiles. Over each profile, all sensors sampled every 10 seconds in the top 150 m of the water column, which corresponds to a vertical resolution of 0.6–1.0 m. Adult female NFS dives rarely approach this depth in the CC [1], [25]. Samples were collected every 20 seconds to 300 m and every 30 seconds between 300 m and 1000 m. Seagliders made 63 crossings along the northern transect (Cape Flattery; CF) and 62 along the southern transect (Grays Harbor; GH) and achieved near-continuous coverage from August 2003 to January 2009 (Fig. 2). Hardware faults resulted in some data gaps, the longest of which was from mid-January to early April 2008 (Fig. 2B). Seaglider data coverage overlaps with the period during which the most satellite-tagged females were present in the CC and GA LMEs, providing good coverage of the overwinter periods between 2003–04 and late 2008-early 2009 (Fig. 2B).

### Seaglider Data Processing

Seaglider observations of seawater conductivity, temperature, and pressure were used to derive profiles of salinity and density as described in [70]. In contrast to shipboard measurements, flow of seawater past the Seaglider conductivity sensor is not driven by a pump, and is instead flushed by the motion of the vehicle. This can produce large spikes or biases in salinity if not properly accounted for. The post-processing procedure outlined in [70] makes corrections for this where possible or discards portions of profiles that cannot fully be corrected. A detailed description of the correction applied to Seaglider salinity measurements is forthcoming [Eriksen, CC; unpublished data]. Seaglider measurements of salinity are accurate to 0.03 (parts per thousand, hereafter presented without units) in regions of strong vertical temperature gradient or 0.01 in other regions of the water column, and temperature sample accuracy is 0.003°C.

Mixed-layer depth for each profile was calculated based on the density step algorithm of de Boyer Montegut et al. [71], using a density step equivalent to a 0.2°C temperature decrease from a 10 m reference value. Although profile data are used where possible, observations for each 2-week crossing were also interpolated to a grid with regular horizontal and vertical spacing as described in [70].

The fluorescence and optical backscatter sensors (collectively referred to as the optical sensors) fielded on WA Seagliders provide qualitative information about the distribution of phytoplankton pigment and biomass. We report results from the fluorescence sensor only. Perry et al. [68] and Sackmann [69] analyzed the Seaglider optical data through the year 2007, including comparison to satellite-inferred surface Chl*a*, and we followed their procedures for processing and interpretation. Starting from reported digital sensor counts at each sample, we subtracted a background offset unique to each sensor, determined from *in situ* measurements in clear, dark water, and then applied the manufacturer's calibration formula to convert fluorescence counts above background to Chl*a* concentration (mg m^−3^). We used night values of Chl*a* only in order to avoid the effects of fluorescence quenching, which produces a low bias in daytime measurements collected near the surface [68], [69], [72]. It should be emphasized that absolute concentrations derived using this method, and reported herein, are unreliable due to the unavailability of contemporary shipboard *in situ* data for comparison. However, relative temporal and spatial structure measured by the sensors is robust [68], [69], [73]. For an extended description of the fluorescence processing, see the supporting information (Supporting Methodology S1).

### Seaglider Analyses

For physical and optical variables, we generated an average annual cycle for the surface ocean zone (top 150 m) within a cross-shore band 60 to 80 km from the shelf break. Observations of Chl*a*, salinity, and density anomaly (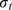; kg m^−3^) in this zone were sorted into bins by depth and days since 1 January of each year. Bins had 6 m vertical and three-week temporal width and 66% overlap between adjacent bins, i.e., adjacent bins have their center one week apart in time and 2 m in depth. The overlap between bins acts to smooth the resulting averages. The amount of overlap was chosen in order to reduce noise in the average annual cycle that is generated by interannual variability in the timing of events such as the spring mixed layer shoaling and phytoplankton bloom. The mean value in each bin was taken first for all observations within each year and then the median value of these means was taken across years.

### Statistical Analysis of Behavior Relative to Seaglider Data

The upper-ocean properties observed from Seagliders were used to investigate seasonal trends in adult female daytime diving in the CC ecosystem. The choice of daytime diving characteristics was motivated by a parallel study of NFS migration during a single year, in which Sterling et al. [25] observed a single adult female enter the CC ecosystem and increase the proportion of dives which occurred during daytime relative to all other ecosystems. Specifically, we examined the average depth of female dives in 6 h daytime periods, defined as those with >80% proportion daylight, along with Seaglider observations of MLD in the region 60–80 km from the shelf break, versus days since 1 January using a generalized additive model (GAM). A GAM is a model in which the assumption of a linear response to predictor variables, even in transformed space, is relaxed and the predictor terms may take arbitrary form [65]. The GAM is an appropriate choice for modeling these response variables since we expect that some portions of their winter-summer evolution may be nonlinear or discontinuous, particularly in the case of MLD which shoals abruptly after the spring transition [68]. In our case, we used a GAM to model log-transformed depth – either depth of the surface mixed layer or depth of day dives averaged in 6 h periods – using an intercept and two predictor terms that are functions of yearday. The model takes the form
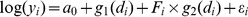
(1)where 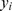 is the depth (either MLD or seal dive depth) and 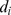 the day of observation 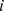, 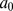 the intercept, 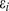 the residual, and 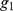 and 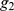 are arbitrary functions to be estimated. 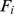 is the interaction coefficient, which is set to 0 for observations of MLD and 1 for observations of average day dive depth. Thus, the function 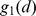 is a fit to the annual cycle of MLD over the months January–June while 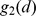 quantifies a possible time-dependent offset of adult female NFS day dive depths relative to the MLD.

Each predictor term is in practice a locally weighted regression of the observations and thus acts similar to a smoothing filter or running average. The model fit is accomplished by minimizing the negative penalized log likelihood function, which considers not only the model disagreement from data but also the “roughness” of the empirical functions 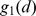 and 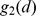. Thus the GAM is intermediate between a function that produces an exact fit to all observed MLD/average day dive depth observations (e.g., spline interpolation) and one that applies a linear fit to all observations in each category versus yearday (analysis of covariance). The degree of compromise between these two – the relative weight applied to model misfit versus model roughness – is determined through cross-validation. The GAM was implemented in R using the *gam* package V1.09. Residuals from the GAM fit were approximately normally distributed and did not show significant evidence of nonstationarity (Fig. S1).

### Supplementary Environmental Data

To compare seal tracks to mesoscale oceanographic circulation, we utilized gridded SLA and surface geostrophic velocity anomaly products obtained from the Archiving, Validation, and Interpretation of Satellite Oceanographic data (AVISO) Reference Series merged delayed-time product (http://www.aviso.oceanobs.com). From these we calculated EKE [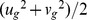 (m^2^ s^−2^), where 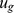 and 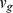 are zonal (positive eastward) and meridional (positive northward) geostrophic velocity anomalies, respectively]. To identify coherent mesoscale features and compare female movements to eddy locations in regions outside of the Seaglider transect pattern, we used eddy trajectories of Chelton et al. [63], available online from http://cioss.coas.oregonstate.edu/eddies/. This dataset also utilizes the AVISO Reference Series gridded SLA product, giving eddy center latitudes and longitudes, length scales (degrees of arc length), polarities (cyclonic or anticyclonic), and strengths (in cm of SLA) at 7 d time steps for the period October 1992 to January 2011 at the time of download. Chelton et al. [63] report eddy length scales (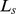) as the radius of a circle with area equal to that enclosed by the contour of maximum velocity around each eddy. It should be noted that an individual eddy is in fact rarely circular, though consistent radial structure does appear when averaging over a large number of eddies [63]. The gridded SLA data used for qualitative interpretation in our analysis, and also used for eddy detection by Chelton et al., [63] are produced by interpolating and smoothing the output of two altimeters. Chelton et al. [63] analyzed the approximate spatial and temporal filtering characteristics and found that eddies with spatial scales of 30–40 km were the smallest that the gridded product could resolve at the latitudes considered in this study. Altimetry-resolved eddies in the CC have horizontal radii typically ranging from 60–90 km, and for long-lived coherent features, lifetimes on the order of weeks [74]. Mean propagation speeds are ∼0.05 m s^−1^ or less at the latitudes considered in this study, which indicates that eddies move relatively slowly in comparison to the 7 d gridded altimetry time scale.

Several environmental variables were utilized for qualitative comparison and discussion relative to seal behavior. For spatial representation of surface Chl*a* and temperature patterns relative to Seaglider and seal foraging data we utilized Sea-viewing Wide Field-of-view Sensor (SeaWiFS) and Moderate Resolution Imaging Spectroradiometer – Aqua (MODIS Aqua) Level-2 surface ocean color and temperature swaths, processed and made available online by the National Aeronautics and Space Administration Ocean Color project (http://oceancolor.gsfc.nasa.gov/; [75]). All bathymetry data shown were sourced from the NOAA Earth Topography One Arc-Minute Global Relief Model (ETOPO1) gridded dataset (http://www.ngdc.noaa.gov/mgg/bathymetry/relief.html). We obtained profile data from one Argo float from the United States Global Ocean Data Assimilation Experiment Argo Page (http://www.usgodae.org/argo/argo.html). Wind data for the WA coast were obtained from National Data Buoy Center station Destruction Island (DESW1), available at http://www.ndbc.noaa.gov. Wind vector measurements at DESW1, made at a height of 52.7 m above mean sea level, were daily averaged and rotated into an alongshore/cross-shore coordinate system, with positive alongshore direction (poleward winds) defined as being along 338°T, the local orientation of the coastline near DESW1.

## Results

### Migratory Distribution

Migratory tracks from all 40 females (41 tracks total) are shown in Figure 1. Seals departing St. Paul Island averaged 9.1 d (n = 31, standard deviation [SD] = 5.4 d) to reach the NP (Table 1), while seals from Bogoslof Island took only 2.0 d (n = 10, SD = 1.0 d) due to their closer proximity to the NP (Fig. 1). The earliest arrival in the GA ecosystem was 26 November (mean: 8 January) and the earliest arrival in the CC was 21 December (mean: 26 January). Mean time between departure and first entry to the GA and CC ecosystems was 53.8 d (n = 40, SD = 21.4 d) and 71.3 d (n = 34, SD = 26.4 d) respectively and mean track duration was 138.3 d (n = 41, SD = 57.2 d).

**Figure 3:**
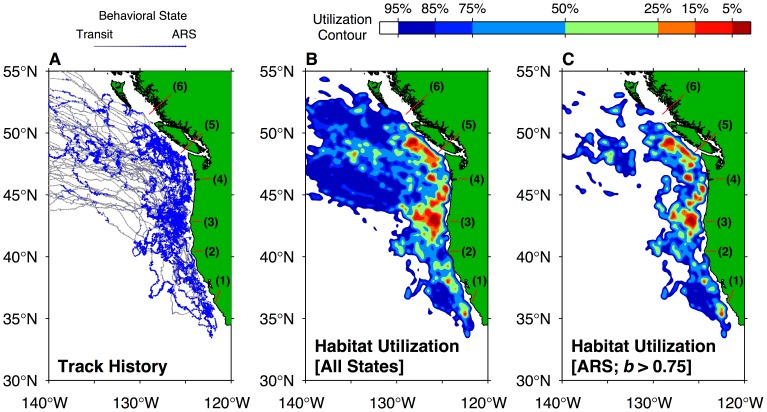
Track density and habitat utilization of adult female northern fur seals in the California Current and southern Gulf of Alaska. (A) Satellite-tracked migratory routes of adult female northern fur seals. Tracks are colored and weighted by estimated behavioral state: thin gray lines correspond to transit behavior, while thick blue lines indicate area-restricted search (ARS, scale at top left). (B) Density-kernel estimate of horizontal habitat utilization in the California Current and southern Gulf of Alaska. Contours indicate the range size enclosed by each rank percentile of habitat utilization; i.e., the 50% contour is the minimum possible area that could be drawn to enclose 50% of female time spent within the domain. These contours are generated from a fixed-kernel habitat density estimate using a 15 km spatial smoothing scale (scale at top right). (C) Area-restricted search habitat utilization estimate – as for panel B, but obtained using a subset of tracking points with seal behavioral state (indicated by variable 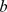) greater than 0.75. In each panel, prominent coastal features are labeled as follows: (1) Point Sur; (2) Cape Mendocino; (3) Cape Blanco; (4) Columbia River; (5) Strait of Juan de Fuca mouth; (6) Queen Charlotte Sound.

Individuals in this study typically entered the CC and GA ecosystems at latitudes between 45°N–55°N and, once in the destination region, foraged within a broad zone between 140°W–120°W, 30°N–55°N (Fig. 3A–C). The 95% habitat utilization contour calculated using only locations within this region extends from ∼54°N to as far south as 34°N (Fig. 3A–B). South of 45°N, fur seal habitat utilization was primarily confined between the coast and 130°W (Fig. 3B). North of 45°N, significant utilization extended west of 130°W, but this was primarily driven by seals arriving into the GA and CC ecosystems during their early migration transit phase (Fig. 3A). Confining the two-dimensional distribution analysis to only locations exhibiting area-restricted search behavior (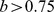) showed that these locations were more closely confined to the coast, with some limited area-restricted search utilization near 135°W, near the eastern terminus of the North Pacific Current, the broad eastward-flowing current that forms the boundary between the northeast Pacific subtropical and subarctic gyres (Fig. 3C; [76]).

**Figure 4:**
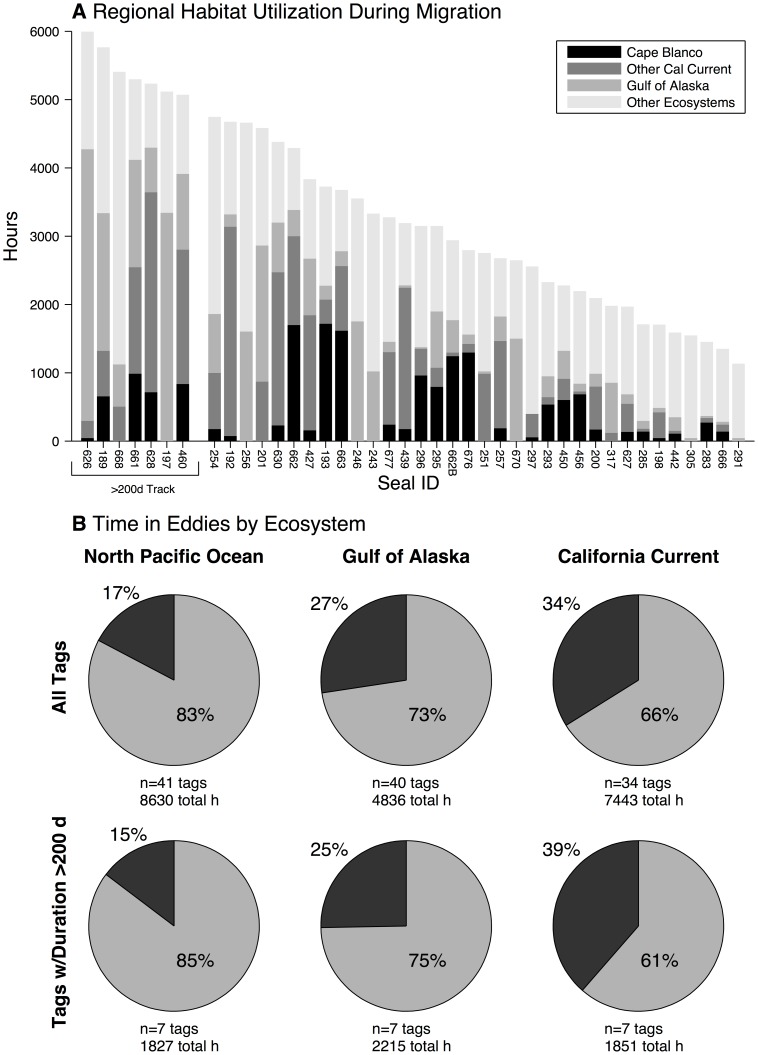
Regional habitat utilization and time spent in eddies by adult female northern fur seals. For each satellite-tracked migratory seal, panel (A) shows number of hours spent in the Cape Blanco region (black, defined as 41.5°N to 44.5°N and east of 128.5°W), other portions of the California Current (dark gray), the Gulf of Alaska ecosystem (medium gray), and all other ecosystems (light gray). (B) The fraction of total female time spent within 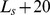 km of the center of mesoscale eddies as identified by Chelton et al. [63] in each ecosystem, where 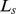 is each eddy's reported radial length scale. The first row displays results for the full dataset, while the second row shows results for only seals with a tracking duration >200 d.

The area-restricted search-only distribution shows several distinct regions of concentrated adult female habitat utilization, the largest of which is near Cape Blanco, Oregon (OR, USA; 42.84°N). For each track, Figure 4 displays the amount of time spent in four migratory regions: the coastal zone proximate to Cape Blanco (defined as between 41.5°N–44.5°N, and east of 128.5°W), portions of the CC ecosystem excluding Cape Blanco, the GA ecosystem, and all other ecosystems. Twenty migratory females spent at least one week in the Cape Blanco zone, while 9 spent more than one month, confirming that the elevated utilization of this zone was not driven exclusively by a relatively small number of animals (Fig. 4A). Figure 4A also demonstrates the consistency across the animals in this study of migratory transit duration between the breeding grounds and destination ecosystems – this is shown by the length of time spent in ecosystems outside the CC or GA in each track (labeled “Other Ecosystems” and indicated by the lightest gray shading in Fig. 4A). For tracks with shorter tag lifetimes, time spent in the CC or GA was reduced, but time spent in other ecosystems, presumably during the transit phase, remained relatively constant. As females moved closer to the continental margin, and eddy generation regions within the CC and GA eastern boundary current systems, their fraction of time spent within 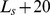 km of the center of identified coherent eddy features increased by a factor of 2 (Fig. 4B). This signal was most pronounced in the 7 of 41 tracks with duration >200 d (Fig. 4B bottom).

**Figure 5:**
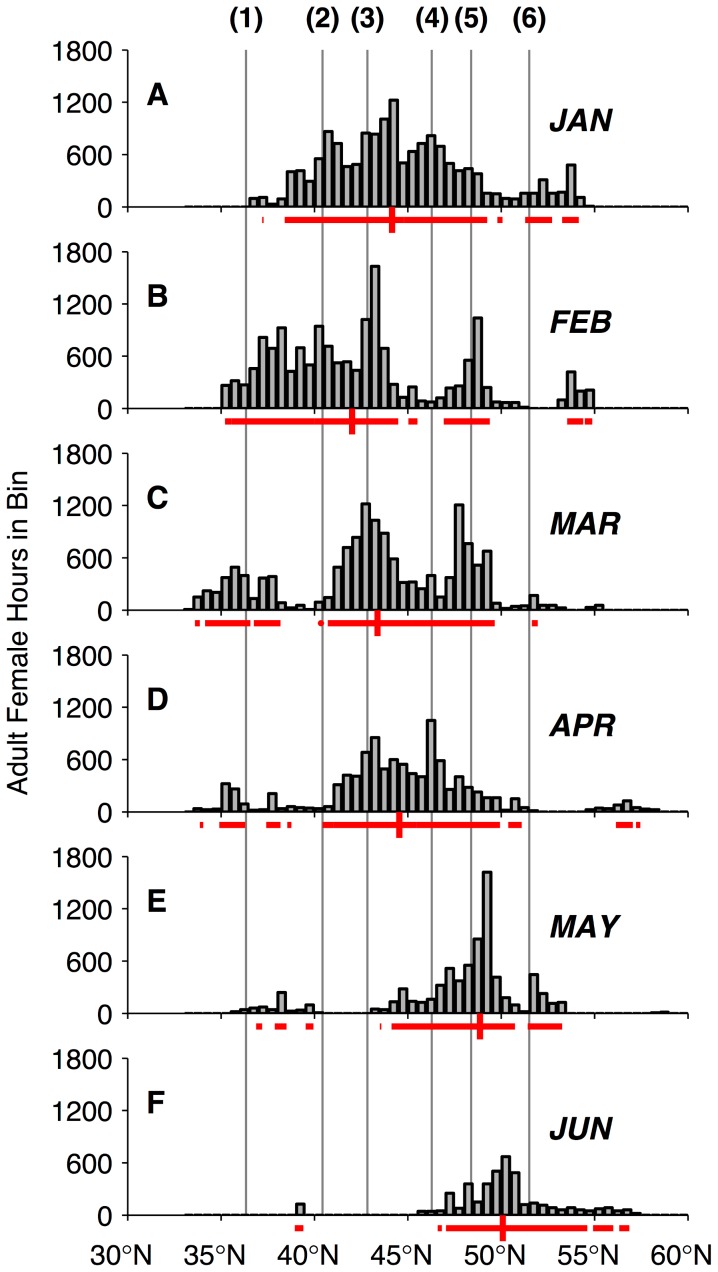
Monthly evolution of adult female northern fur seal alongshore distribution. January (A) through June (F). Gray bars in each plot are a histogram of time spent in that month in 0.5° latitude bins within the California Current and Gulf of Alaska Large Marine Ecosystems east of 145°W, during the winters 2002–03 to 2009–10. Red horizontal lines below A–F indicate the latitudes in which 95% of time spent occurred in that month. Vertical red lines indicate median latitude of the alongshore distribution. Vertical gray lines extending through panels A–F correspond to the latitudes of prominent coastal features, illustrated in Figure 3, which are also numbered above panel A in this figure as follows: (1) Point Sur; (2) Cape Mendocino; (3) Cape Blanco; (4) Columbia River; (5) Strait of Juan de Fuca mouth; (6) Queen Charlotte Sound.

Adult female distribution as a function of latitude along the continental margin was not static throughout the overwintering period, reflecting large-scale ecosystem seasonal patterns and migratory pressure to return to the breeding grounds late in the overwintering period (Fig. 5). Adult female time spent off California peaked in February and declined from March onwards. As females began traveling northward for the return leg of their migration, the median latitude of their distribution shifted northward to the OR and WA coasts in March and April and British Columbia (BC, Canada) in May and June (Fig. 5D–F). Females occupied latitudes off the OR, WA, and southern BC coasts consistently from January–April and some remained off the WA and BC coasts into May and June. In addition to Cape Blanco (January–April), the latitudinal distribution exhibited local peaks of female time spent near other prominent coastal topographic, riverine, or inlet features such as the Columbia River mouth (April), the Strait of Juan de Fuca, (February–March, May), Queen Charlotte Sound, BC (May–June), and Point Sur/Monterey Bay, California (February–April). Females also spent elevated time near latitudes corresponding to Cape Mendocino, California (January–February), though examination of the two-dimensional habitat utilization shows that this time was farther offshore than near other coastal capes.

**Figure 6:**
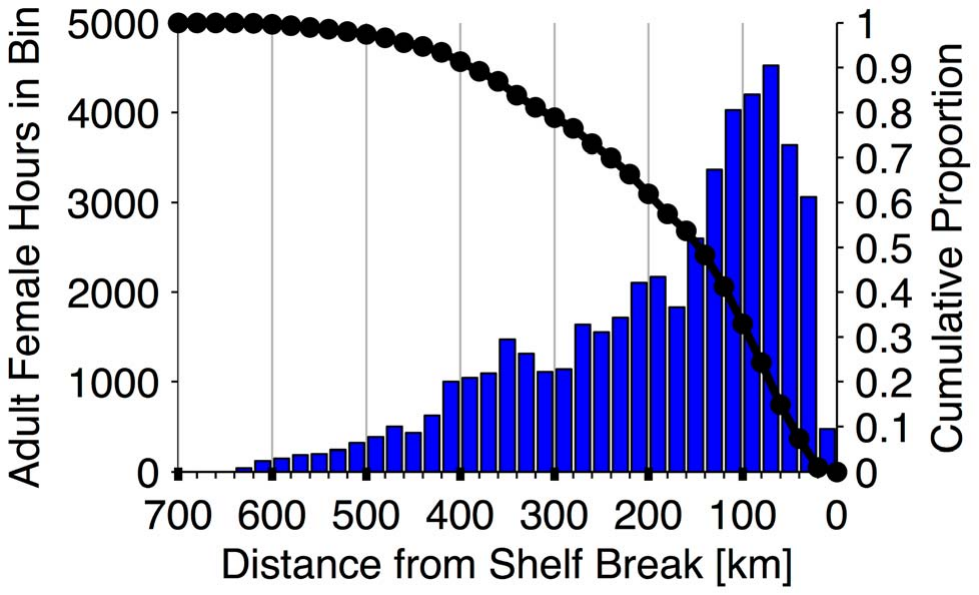
Cross-shore distribution of adult female northern fur seals. Figure depicts time spent versus distance from the shelf break, binned in 20 km segments (blue bars), by seals from 41°N to 52°N over the study duration. These latitudes were chosen to represent the region where the majority of adult female habitat utilization occurred. Cumulative proportion of female time spent from the shelf break (200 m isobath) shown in black.

Seals in the CC and GA ecosystems spent the majority of their time between 41°N and 52°N (72.0% of 68,016 total off-shelf hours in the months January–June; Fig. 5). From March onwards, females spent 80.6% of their time in these latitudes. The cross-shore distribution of time spent within this zone peaked between 60 and 80 km from the shelf break (Fig. 6). Females spent 33% of their off-shelf time within 100 km of the shelf break and 62% within 200 km, the latter of which is the approximate cross-shore zone sampled by Seagliders off WA (Fig. 2). The median (50% of time spent) of the cross-shore distribution occurred at 146 km offshore from the shelf break. When evaluating the cross-shore distribution by month (Fig. S2), the median value had a maximum of 260 km (January) and minimum of 71 km (May).

### Diving and Movement Behavior

**Figure 7:**
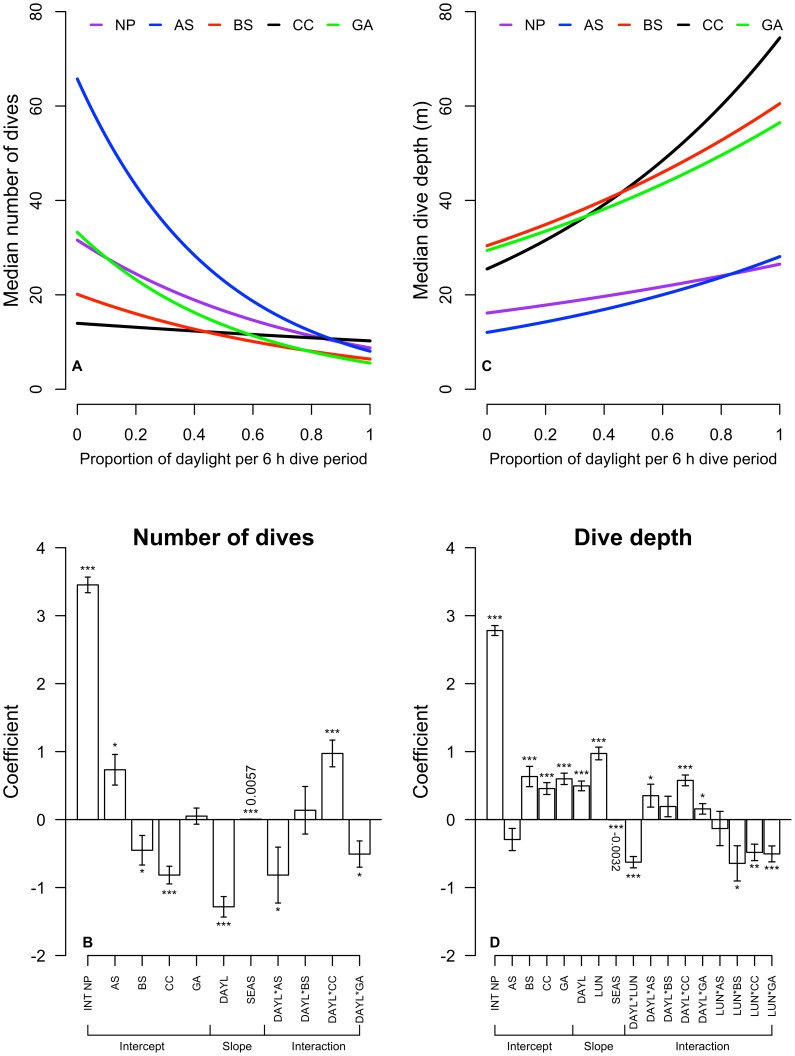
Generalized linear mixed-effects model (GLMM) results for adult female northern fur seal dive behavior. (A) Predicted model response for median number of dives per 6 h period as a function of proportion daylight and ecosystem [North Pacific (NP) = purple, Alaska Stream (AS) = blue, Bering Sea Shelf (BS) = red, California Current (CC) = black, and Gulf of Alaska (GA) = green]. The CC was the only ecosystem to show a nearly equal amount of dives throughout the diurnal cycle, contrasting all other ecosystems where more dives occurred at night and fewer with increasing proportion daylight. (B) Plot of the coefficients of predictor terms in the best GLMM for number of dives per 6 h period. Each vertical bar corresponds to one term. Size of the bar indicates its magnitude (positive = increasing number of dives per 6 h period, scale on y-axis) and whiskers indicate standard error. Terms are labeled below the x-axis and grouped by intercept, main effects or slope, and interaction terms. Asterisks above or below each bar indicate the significance level of each term: 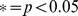; 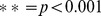; 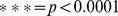. Coefficients are labeled where bars are too small to see. Abbreviations, in addition to ecosystems labeled above, are as follows: INT NP (intercept, North Pacific base model), DAYL (proportion daylight per 6 h period), SEAS (season, days since 1 October). Note that while the season term is small, its effect may be large since season has values that range from 0 to 297 d, while proportion daylight, as an example, varies from 0 to 1. (C) Modeled response for average dive depth per 6 h period as a function of proportion daylight and ecosystem, symbols as in panel A. The CC also showed the greatest response of increasing average dive depth with increasing proportion daylight. (D) Schematic plot of coefficients for terms in the best GLMM for average dive depth per 6 h period, symbols as in panel B. Lunar fraction is abbreviated by LUN.

The top-ranking GLMM for seal number of dives per 6 h period identified proportion daylight, ecosystem, season, and the interaction between ecosystem and proportion daylight as significant predictors (Table 3). In the NP (the base model) increasing proportion daylight was a predictor of fewer dives per 6 h period (Fig. 7A–B). However, the intercepts for the AS, BS, and CC ecosystems differed, resulting in more dives in complete darkness in the AS ecosystem and fewer dives in complete darkness in the BS and CC ecosystems (Fig. 7A–B). The slope of the ecosystem and proportion daylight interaction term in the CC differed from the NP ecosystem due to more female dives occurring during the daytime as they entered coastal transition zone foraging habitat off the coast of North America (Fig. 7A–B). This result is contrary to other ecosystems considered – in the CC, adult female dives were evenly spread throughout the day and showed virtually no correlation with proportion daylight per 6 h period (Fig. 7A).

For seal average dive depth per 6 h period, the top-ranking model identified proportion daylight, ecosystem, lunar fraction, season, and the interactions between proportion daylight and ecosystem, proportion daylight and lunar fraction, lunar fraction and ecosystem as significant predictors (Table 3). Proportion daylight and lunar fraction were both associated with increased average dive depth per 6 h period (Fig. 7C–D). Both of these terms affect the depth distributions of diel migrating prey fields, which respond to increasing light levels regardless of whether they are due to the sun or moon. Ecosystem interactions were important in altering the modeled response of average dive depth to increasing proportion daylight, with the CC ecosystem having the strongest response and deepest average depth during daytime (Fig. 7C). Furthermore, season was a negative predictor of average dive depth per 6 h period, as dives tended to shoal later in the overwintering period (Fig. 7C–D). When compared to the NP, seal average dive depths in all other ecosystems except the AS were deeper at night and were less affected by lunar fraction (Fig. 7C–D).

**Figure 8:**
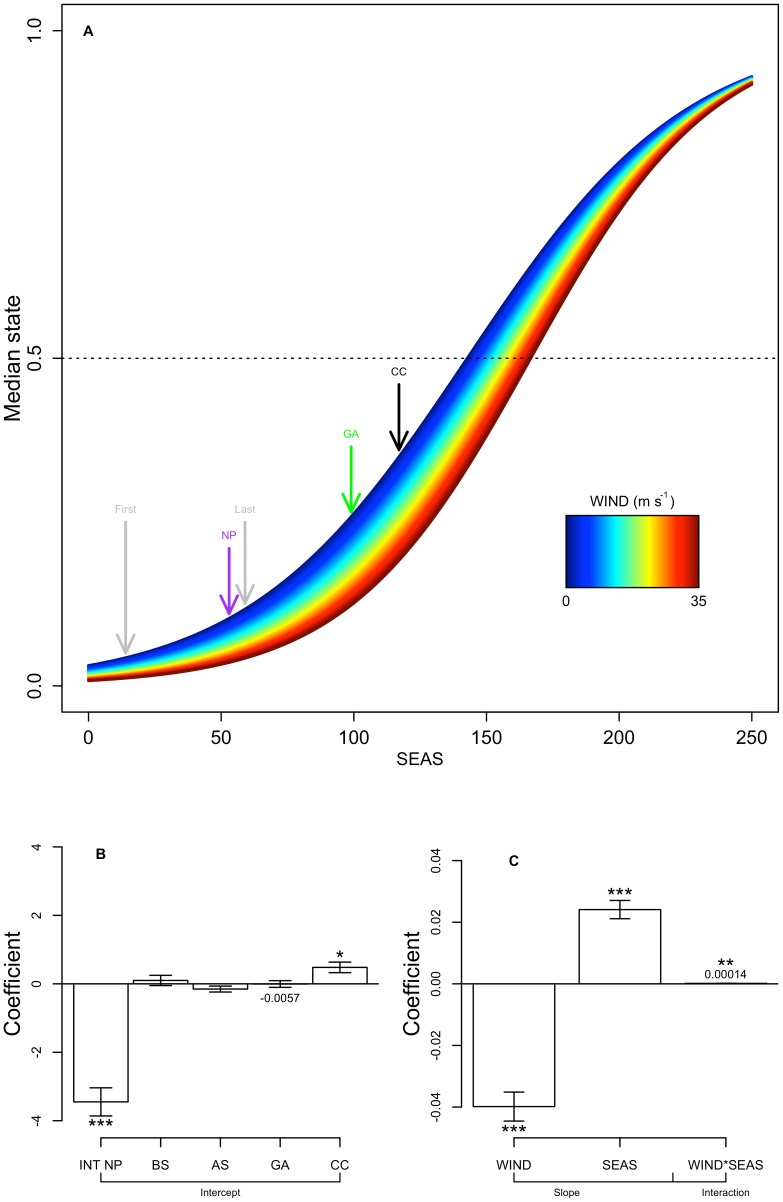
Generalized linear mixed-effects model (GLMM) results for adult female northern fur seal estimated behavioral state. (A) Predicted model response for behavioral state (0 = transient, 1 = area-restricted search) as a function of season (SEAS, days since 1 October, x-axis) and surface wind speed (WIND, m s^−1^, indicated by width of curve and color scale). Note that the surface wind speed effect diminishes with increasing days since 1 October, as indicated by curve width decreasing after reaching its maximum at ∼125 d. Vertical arrows indicate first and last dates of departure from the breeding grounds (gray), and mean date of first entry into North Pacific (NP, purple), Gulf of Alaska (GA, green), and California Current (CC, black) ecosystems. (B) Plot of intercept terms in the best GLMM for behavioral state. Abbreviations as follows: INT NP (intercept, North Pacific base model), BS (Bering Sea Shelf), AS (Alaska Stream), other abbreviations as in panel A. Bar size gives coefficient magnitude and whiskers indicate standard error. Asterisks indicate the significance level of each term: 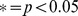; 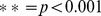; 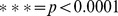. Coefficients are labeled where bars are too small to see. (C) Plot of the coefficients for main effects or slope and interaction terms in the best GLMM for behavioral state – symbols as in panel B, though note difference in y-axis scale. Positive values indicate increasing state (towards area-restricted search).

Surface wind speed, season, and ecosystem were factors consistently identified in the top-ranked models for seal behavioral state (Table 3). Eddy kinetic energy was not found to be an important predictor in the top three models. Increasing surface wind speed was a predictor of reduced behavioral state, which indicates a tendency away from area-restricted search and towards transient behavior with higher wind speeds (Fig. 8A). Of the ecosystem intercept terms in the best model for behavioral state, only the CC was significantly different from the NP ecosystem, reflecting increased behavioral state within the CC (Fig. 8A–B). Season was a positive predictor of behavioral state, reflecting the strong tendency towards transit during the initial movement across the NP and more area-restricted search behavior after mid-February when most females had arrived to their destination areas (Fig. 8A, C). The season/wind speed interaction term shows that this effect is strongest early in the migratory period, as high surface winds have a diminished effect on behavioral state with time (Fig. 8A, C).

### Vertical Localization

**Figure 9:**
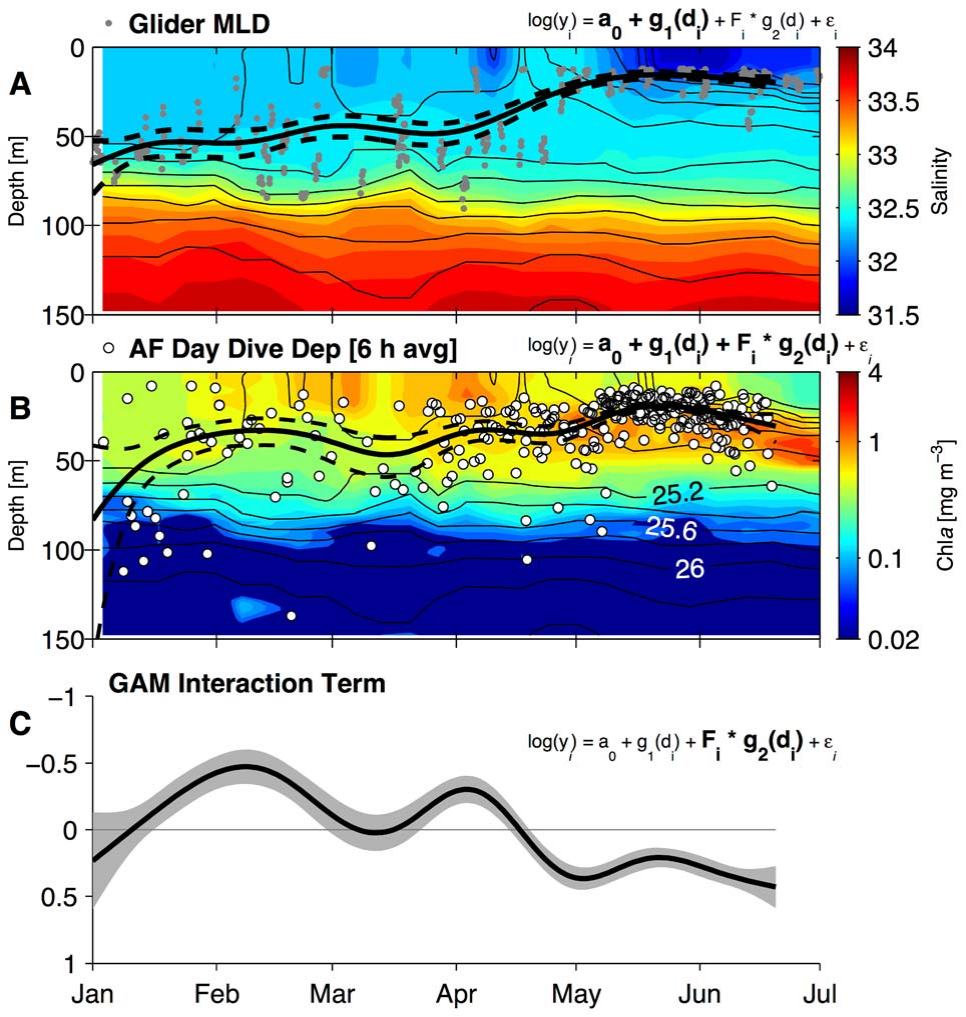
Composite annual cycle of upper-ocean characteristics, mixed-layer depth (MLD) and adult female northern fur seal daytime diving. (A)–(B) Colors show average annual cycles of salinity (panel A) and chlorophyll-*a* (Chl*a*, panel B) collected 60–80 km off the Washington shelf break 2003–09 from Seagliders. This region was chosen to correspond with the cross-shore peak of adult female northern fur seal foraging as shown in Figure 6. Only the overwintering portion of the annual cycle is shown. Contours in each panel are average annual cycle of density anomaly (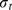, kg m^−3^) in intervals of 0.2 kg m^−3^. Gray filled circles in panel A indicate MLD calculated from individual Seaglider profiles, while white circles in panel B indicate average dive depths in day (>80% proportion daylight) 6 h periods in the California Current ecosystem. Solid black lines in panels A and B display generalized additive model (GAM) results for MLD and female average day dive depth, respectively (dashed black lines are 95% confidence intervals). The GAM model form, equation (1) in the main text, is shown above each panel and terms being displayed in each panel are bold. (C) GAM interaction term (solid line) and 95% confidence interval (gray shading) in log space. This term models the time-dependent depth offset of adult female seal day diving relative to the MLD; a negative interaction term indicates mean diving depths shallower than the MLD. Note y-scale direction in panel C is set positive downwards, in order to match the direction of depth axes in panels A and B.

Generalized linear mixed-effects models indicated that the rate of female NFS daytime diving was greater in the CC than in other ecosystems. The average annual cycle of upper-ocean physical structure, as described by Seaglider data off the WA coast, suggests that the depth of the surface mixed layer may influence seasonal patterns in vertical localization of these daytime dives. The average annual cycle of surface ocean physical properties and Chl*a* obtained from the Seagliders in a region 60–80 km seaward of the shelf break is shown in Figure 9A–B. This zone corresponded with the cross-shore distribution peak of female fur seals. The MLD in this zone off WA reached maximum depths of 45–75 m in January–February and was most variable in March and April. This is partially driven by the occasional presence of Columbia River plume water 60–80 km seaward of the shelf break in these months, which is reflected by low values of average near-surface salinity prior to the average mixed layer shoaling in May (Fig. 9A). The MLD off WA shoals in late April or early May, and average Chl*a* patterns in these months show the onset of the spring bloom in the mixed layer followed by development of a subsurface Chl*a* maximum in June.

Female daytime diving depths shoal and become more vertically localized late in the overwintering period; this pattern suggests an association with the MLD, which exhibits a similar winter-summer progression (Fig. 9A–B). Recall that the GAM quantifies this relationship by seeking a time-varying fit to MLD and female dive depth using two predictor terms in (1). The first term 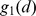 fits only the MLD (Fig. 9A), while the second (interaction) term 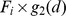 estimates the additive effect of female daytime diving response above or below the MLD and the change of this response with yearday. The total adult female response, MLD+interaction, is shown in Figure 9B; the isolated interaction term is shown in Figure 9C. The GAM results suggest a rough partition of the overwintering period into two regimes, separated by the shoaling of the MLD around 1 May. Prior to this time, the MLD is deep, shoals slowly, and is more variable (Fig. 9A). Female day dive depths are on average shallower than the MLD though the interaction term is not significant for the entirety of this period (Fig. 9C). After shoaling, the MLD is uniformly 15–20 m depth, with much less variability (smaller confidence bounds in Fig. 9A). Female dive depths are also much shallower during this time and are less variable (Fig. 9B). The interaction term changes sign prior to the shoaling and indicates that late spring day diving is on average 5–10 m below the MLD (Fig. 9B–C). The GAM smoothing scale was long enough to avoid effects of the occasional shoaling of MLD due to freshwater plumes, which is not an important part of the annual cycle in other locations in the CC at which female dives were collected.

**Figure 10:**
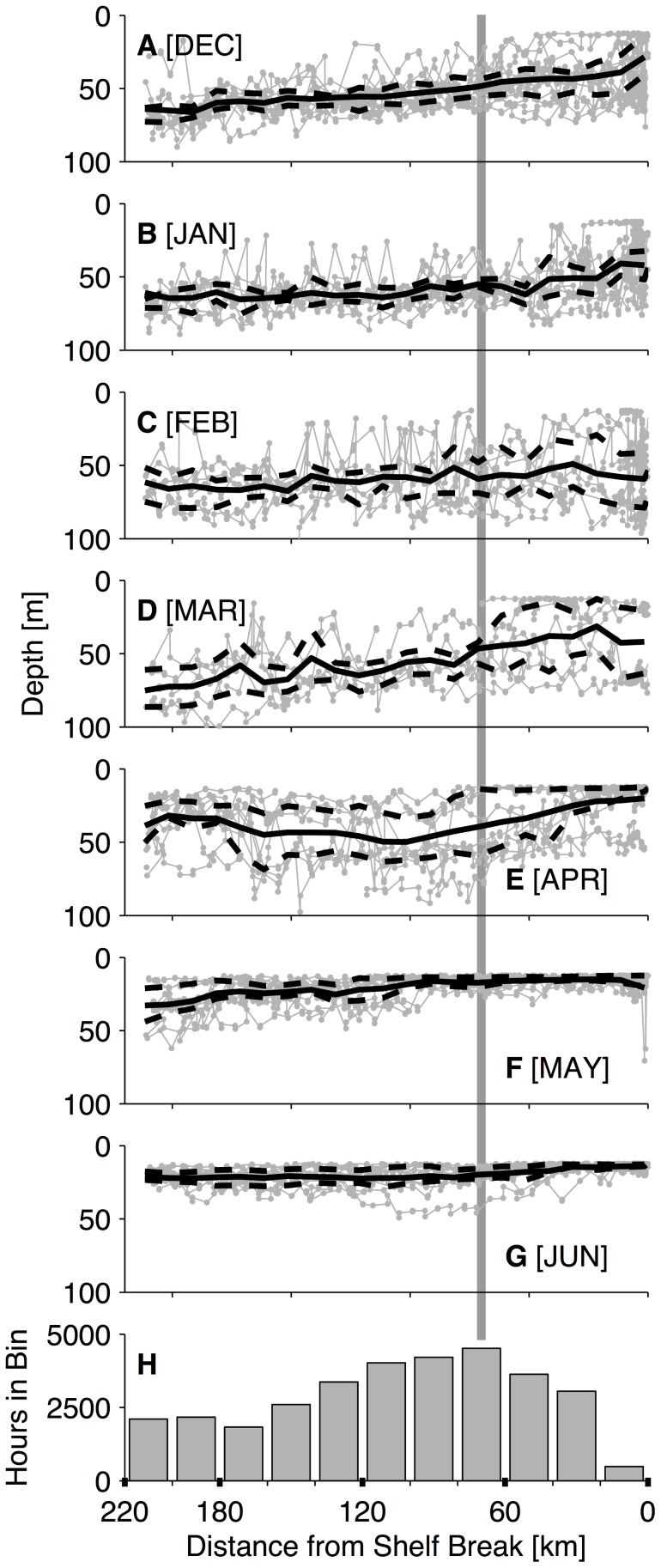
Cross-shore mixed-layer depth profile by month. (A) December to (G) June. Mixed-layer depth calculated from individual Seaglider crossings in gray, mean profile in 10 km bins in black. Dashed black lines in A–G indicate the 1^st^ and 3^rd^ quartile of the observations within each bin. Panel H shows a portion of the cross-shore distribution of adult female northern fur seal time spent versus distance from the shelf break, truncated at 220 km offshore (the full distribution is shown in Fig. 6). The vertical gray line through all panels corresponds to the station of the bin with the greatest amount of female time spent in panel H.

Seaglider surveys observed MLDs that were on average shallower near the coast throughout much of the overwintering period (Fig. 10A–G). The offshore-inshore difference in MLD varied throughout the year and was greatest in both March (Fig. 10D) and September–October (not shown). The peak in the adult female cross-shore distribution (Fig. 10H) corresponded to the region of steepest onshore shoaling in March and April. It should be noted that the late winter cross-shore difference in average MLD off WA is probably driven in part by freshwater plumes from the Columbia River or other smaller rivers along the WA coast [77], and may not be representative of all locations in the CC. Washington coast Seagliders recorded negligible cross-shore gradients in summer (Fig. 10F–G), when strong surface heating and weaker wind stress prevail over the region.

### Links to Mesoscale Circulation and Coastal Topography

The distribution results show that female habitat utilization during the overwintering period is concentrated off the shelf but within the inner coastal transition zone, and that the fraction of time spent near eddies increases twofold in the CC ecosystem (Fig. 4B). Examination of individual animal tracks suggests that females respond behaviorally to some (though not all) mesoscale eddies and jets by altering movement and behavioral state. Examples of this are shown below for tracks of four animals, one of which additionally foraged near a Seaglider, which will be described separately in the section *Individual Case Studies*.

**Figure 11:**
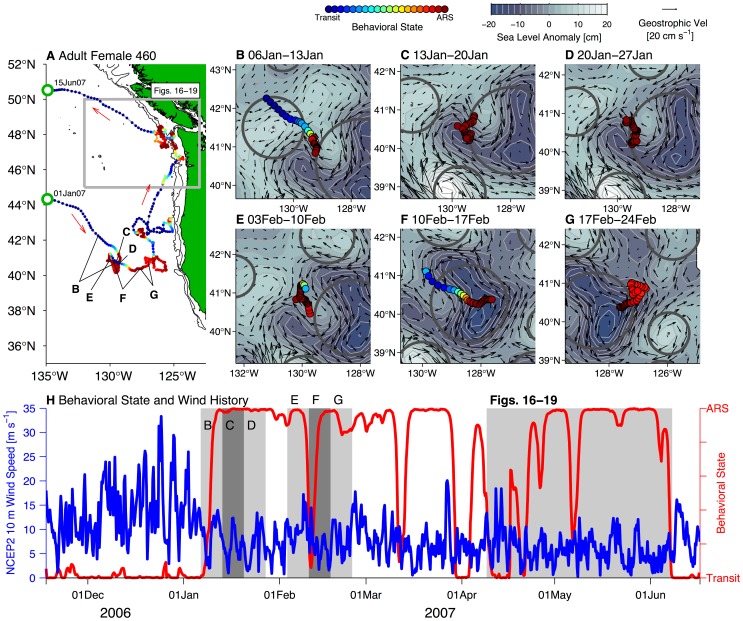
Behavioral responses of adult female northern fur seal 460 to mesoscale circulation and surface wind speed. (A) Overview of satellite-tracked locations of female 460 in the California Current and Gulf of Alaska ecosystems from 01 January 2007 to 15 June 2007. Six h locations are colored according to estimated behavioral state (scale at top, ARS = area-restricted search). Gray box in panel A and gray shading in panel H indicates the spatial and temporal extent covered by Figures 16A–19A, when female seal 460 foraged in close proximity to the Seaglider region. (B)–(G) show weekly intervals of 460's estimated locations and behavioral state in 6 h periods (filled color circles, scale at top) plotted over sea level anomaly (color contours) and surface geostrophic velocity anomaly (Vel, black arrows, scale at top right). Each plot is centered on female seal 460's locations over the weekly period. Note that one weekly interval is omitted between panels D and E, during which time female seal 460 continued to forage in the same location as in panel D. Thick gray circles indicate the locations and approximate spatial extent of altimetry-identified mesoscale eddies from Chelton et al. [63]. Eddies are plotted as circular features though this is intended for illustration purposes only. (H) Plot of estimated behavioral state (red line, scale on right y-axis) and 10 m height wind speed (m s^−1^) at seal 460's location (blue line, scale on left y-axis) versus time for the overwintering period 2007–08. Wind speed estimates are obtained by interpolating National Centers for Environmental Prediction Reanalysis 2 (NCEP2) product to 460's estimated locations at each 6 h time point. Gray bars in alternating shading display the extent of time covered by panels B–G.

Female seal 460, tracked during the 2006–07 overwintering migration, was one of seven individuals with track duration of >200 d (Fig. 4A); she entered the CC ecosystem from a more southerly route than was typical of other tracks (Figs. 3A, 11A). Female 460 transitioned to area-restricted search behavior coincident with her encounter with the edge of a mesoscale cyclonic eddy (counterclockwise-rotating, locally low SLA) on 9 January 2007. This followed a prolonged period of transit, during which time 460 bypassed [25] an elongated anticyclonic (clockwise, locally high SLA) feature immediately to the northwest of where she began foraging on 9 January (Fig. 11A–B). Panels B–G of Figure 11 illustrate weekly portions of the following two-month period in which 460 foraged directly west of Cape Mendocino. On 9 February, 460 began transit movement to the SE, possibly initiated by increased surface wind speeds (Fig. 11H), and began following currents in an adjacent meander, before foraging near the NNE edge of a cyclone/anticyclone dipole (Fig. 11E–G). Female seal 460 later moved north, spent some time to the west of Cape Blanco, and foraged for two months off the WA coast. Figure 11H shows other instances in 460's track in which alterations in behavioral state over time periods of ∼2–3 d were associated with increases in surface wind speed. Additionally, this panel illustrates that female 460 experienced overall lower wind speeds while foraging in the CC and GA in comparison to the early migratory transit phase (prior to 9 January). Similar instances of altering movement behavior in response to some mesoscale features were found in other satellite tracks in this dataset and two are included in the supporting information (Figs. S3, S4).

**Figure 12:**
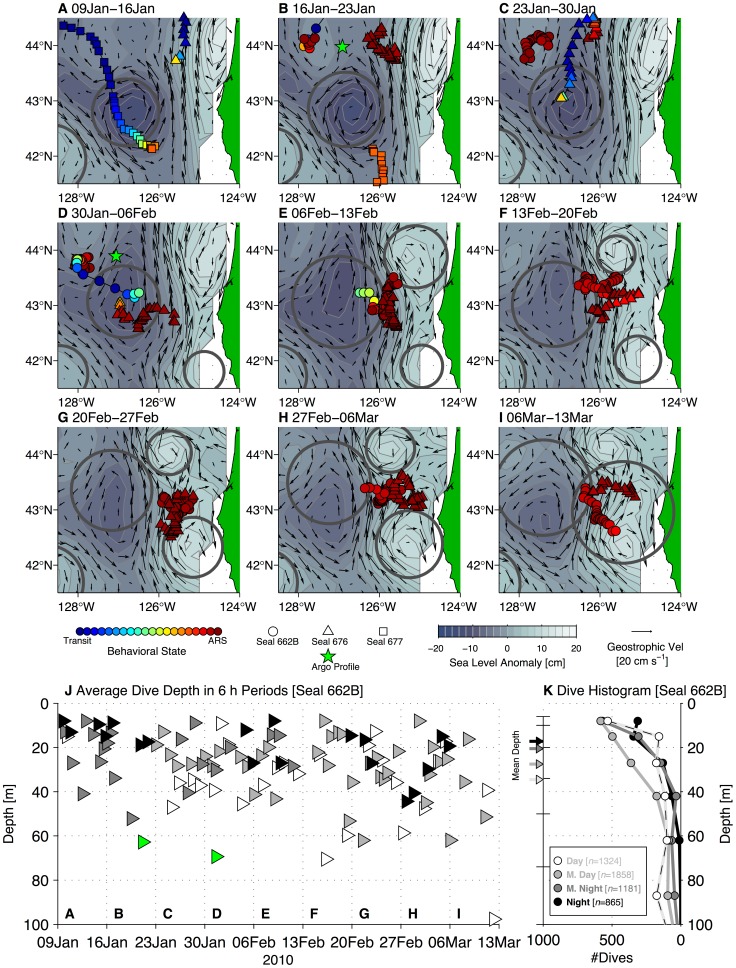
Winter surface circulation at Cape Blanco in 2010 and habitat utilization by three adult female northern fur seals. (A)–(I) Weekly intervals of sea level anomaly (color contours, scale below panel H), surface geostrophic velocity anomaly (Vel, black arrows, scale below panel I), and estimated locations of three satellite-tagged adult females (colored by estimated behavioral state, scale below panel G, ARS = area-restricted search) in the Cape Blanco region in 2010. Symbol types for each individual are also shown below panels G–H. As in Figure 11, thick gray circles indicate the locations and approximate spatial extent of altimetry-identified mesoscale eddies from Chelton et al. [63]. In panels B and D, green stars show the location of two Argo float profiles collected during these time periods. (J) Mixed-layer depth calculated from Argo profiles (green triangles), along with average dive depth in 6 h periods from female seal 662B track. Each 6 h period is sorted into one of four categories based on its proportion daylight (black triangles = night, dark gray = mostly night, light gray = mostly day, white = day, as defined in Results section *Links to Mesoscale Circulation and Coastal Topography*). (K) Histograms, one for each proportion daylight category, showing number of dives in that category that have maximum depth within each dive tag pre-programmed depth bin. Depth bin bounds are indicated on the left y-axis by whiskers. Arrows at left of panel K indicate average dive depth by proportion daylight category. Symbol shading follows that of panel J.

Observations of movement and behavioral state changes toward area-restricted search in association with mesoscale eddies suggests one possible reason for increased female density near coastal capes such as Cape Blanco and Cape Mendocino: these irregularities in the continental shelf influence alongslope currents, creating instabilities, meanders, and eddies. Figure 12 displays an example of observed eddy generation at Cape Blanco in the 2009–10 overwintering period, and utilization of this region by three separate adult female NFS. One of these individuals (track 662B; triangles) carried a dive recorder and entered the Cape Blanco region on 21 January, stopping in a small, weak anticyclonic feature on the northern edge of a larger cyclonic eddy centered at 43°N, 126.5°W (Fig. 12B–D). Another individual (677; squares) had passed through this region a week prior but remained in transit mode (Fig. 12A). Female 662B foraged for three weeks on the NW side of the cyclone before moving across the eddy to the east and foraging within a strong poleward jet that formed the boundary between the offshore cyclonic feature and a newly developing anticyclone on the inshore side of the jet (Fig. 12E–I). Another satellite-tagged female (track 676; circles) also foraged within a similar spatial extent from 6 February onwards, appearing to utilize the same mesoscale habitat feature (Fig. 12E–I).

Dive patterns recorded by female 662B during this period are characteristic of patterns apparent in the statistical analyses of number of dives and average dive depth per 6 h period. Figure 12J–K display average dive depth in 6 h periods versus time and depth histograms of female 662B diving, with each 6 h dive period classified into one of four categories of proportion daylight: day (proportion daylight ≥80%), mostly day (50% ≤ proportion daylight <80%), mostly night (20% ≤ proportion daylight <50%), and night (proportion daylight <20%). The majority of diving during 9 January–13 March was collected during 6 h periods classified as day or mostly day (Fig. 12J–K). Average dive depth increased with increasing proportion daylight (Fig. 12K). Two temperature/salinity profiles were collected near female seal 662B's foraging on 21 January and 1 February from Argo float ID 4900574. Mixed-layer depth, as calculated from these two profiles, was 20–30 m deeper than the average depths of 662B's day dives during this period (Fig. 12J). This is consistent with the results of the GAM for average depth in daytime 6 h period during this portion of the season – i.e., within the mixed layer prior to the transition to spring conditions in late April/early May.

**Figure 13:**
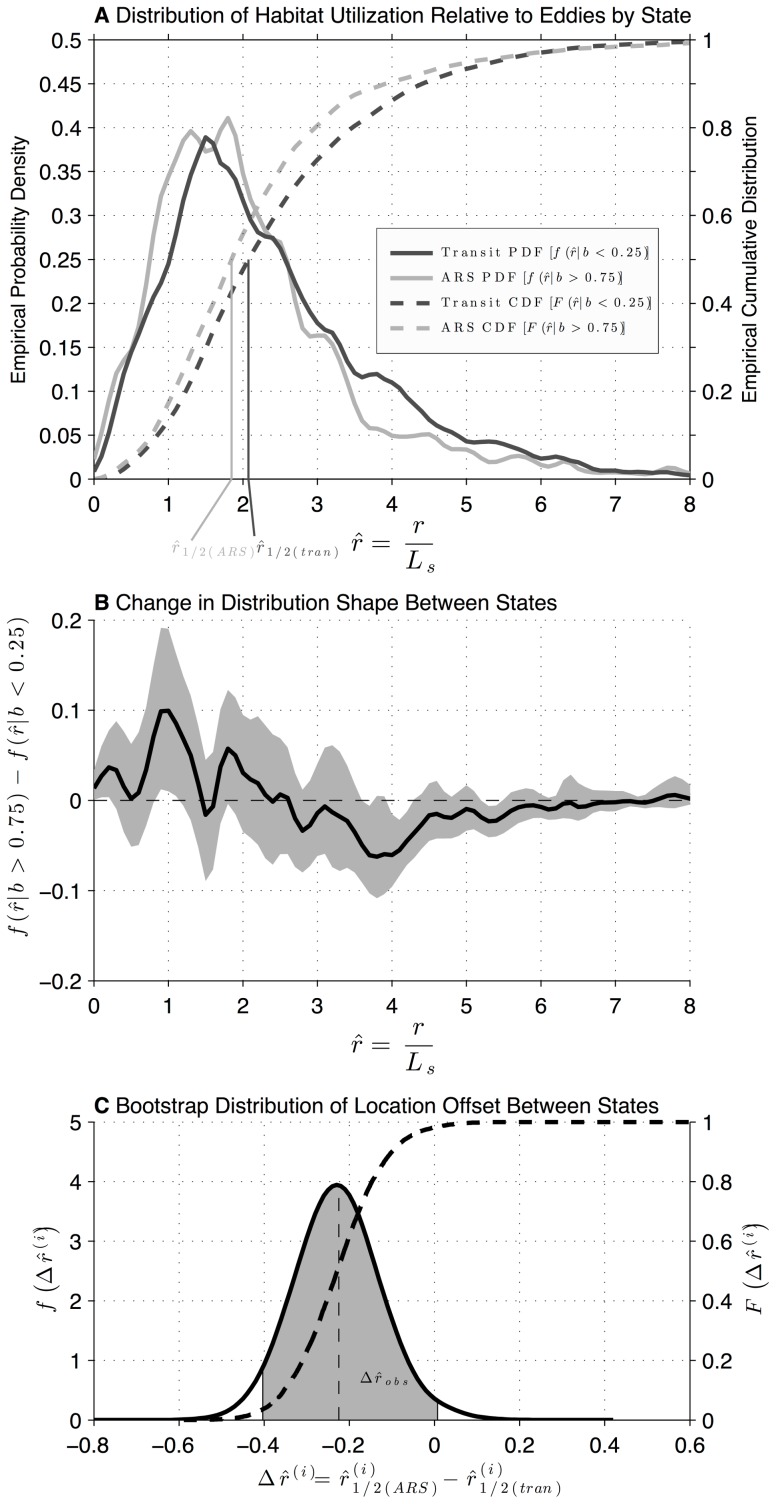
Adult female northern fur seal habitat utilization relative to eddy features, conditioned by estimated behavioral state. (A) One-dimensional radial probability density functions [PDFs, 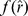] and cumulative distribution functions [CDFs, 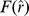] for habitat utilization relative to eddy features at two categories of behavioral state 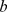 (transit = dark gray, area-restricted search [ARS] = light gray). “Transit” periods are those with 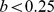; “ARS” periods are those with 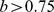. Solid lines show PDFs, dashed lines are CDFs (scale on right y-axis). Distributions are computed as a function of normalized distance to the nearest eddy center 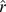, defined as absolute distance to the nearest eddy divided by that eddy's radial length scale 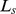 [63]. (B) Observed difference between area-restricted search PDF and transit PDF (solid black line) with 95% confidence bounds computed using a bootstrap method (gray shading). (C) Bootstrap distribution of 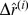, the difference of median values of 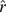 between area-restricted search and transit states on bootstrap iteration 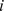. Solid black line shows the PDF of these values, thick dashed line shows CDF (scale at right). Thin vertical dashed line is the observed value of 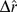. The gray shaded area denotes the bias-corrected/accelerated 95% confidence interval on the observed value of 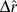. For the observed value of 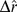 to be significantly different from zero using a two-tailed test, this confidence interval must not include zero.

Though efforts to model the effect of eddies on estimated behavioral state using an EKE term were unsuccessful, adult female habitat utilization relative to coherent eddy features identified by Chelton et al. [63] showed some differences with estimated behavioral state. For both area-restricted search (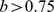) and transit (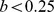), the PDF of female habitat utilization as a function of 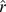 (normalized radial distance from the nearest eddy center) was unimodal with a peak near 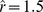 and a long positive tail (Fig. 13A). However, the PDF of area-restricted search points peaked closer to the center and had a weaker positive tail; i.e., area-restricted search locations were more closely aligned with identified eddy features than transit.

For area-restricted search locations, 55.8% were within 2 radii of the nearest eddy center, while this was true of 47.8% of transit locations (Fig. 13A). Differences in PDF shape between states were significant near 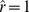 (higher search density; 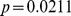, Fig. 13B) and 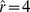 (higher transit density; 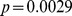). A similar pattern, though with greater differences in PDF shape, was found when performing the same analysis but exclusively for tracks with length >200 d (Fig. S5). These differences indicate that, when in area-restricted search, individuals were more likely to utilize areas near 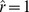 and less likely to utilize areas near 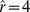 than when in transit. The median value of 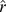 was 1.85 for area-restricted search and 2.07 for transit, though the difference between these two values was not significant based on the bootstrap method (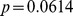, Fig. 13C). The observed difference in median values was enhanced in tracks >200 d and was significant (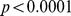, Fig. S5).

### Individual Case Studies

Three females equipped with dive recorders (individuals numbered 460, 626, and 628, Table 1) foraged near Seagliders off the WA coast. The following sections compare the movement and diving records of each individual to subsurface physical and bio-optical oceanographic structure as revealed by the Seaglider vertical profiles. Two animals (female seals 626 and 628; this section) foraged for periods ∼1 week, exhibiting diel diving patterns consistent with those revealed in the statistical analysis. A third animal, female seal 460, foraged for a month within the Columbia River freshwater plume.

#### Diel Diving Patterns

**Figure 14:**
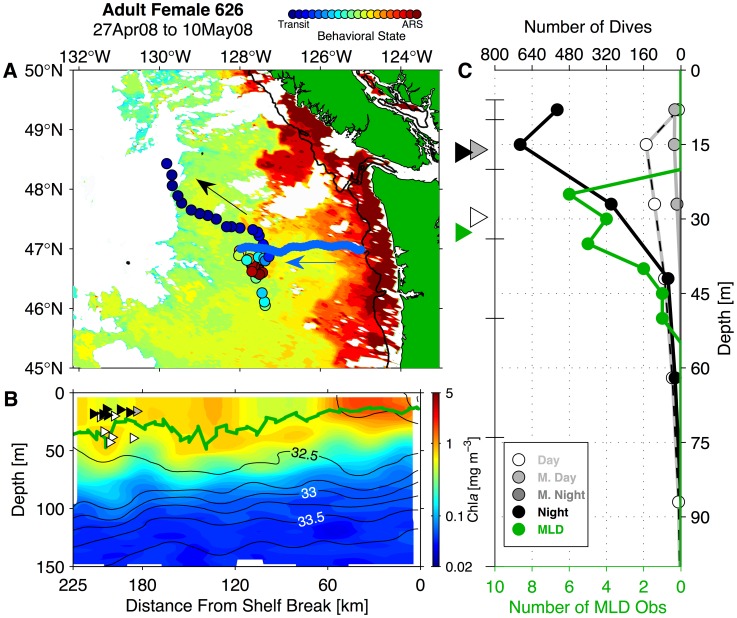
Adult female northern fur seal 626 near Seaglider 101 (SG101) during the time period 27 April to 10 May 2008. (A) Estimated locations of female 626 (filled color circles) and reported GPS locations of SG101 (thick blue line) off the Washington and British Columbia coast (green). The 200 m isobath is indicated by thin black line. Arrows indicate direction of travel of seal 626 and SG101. Colors of filled circles indicate behavioral state (scale at top, ARS = area-restricted search). Background colors in panel A indicate surface chlorophyll-*a* (Chl*a*) in cloud-free regions on 16 May 2008. Chlorophyll color scaling is the same as that in panel B. Blank regions are portions of the image removed due to cloud cover (16 May was the clearest available image from the surrounding time period). (B) Upper-ocean conditions by depth and distance from the shelf break recorded by SG101 during its crossing: Chl*a* (background colors), salinity (black contours), and mixed-layer depth (MLD, dark green line). Triangles indicate average depths of female northern fur seal 626's dives in each 6 h period. Triangles are plotted by cross-shore distance along the Seaglider line, and triangle fill colors indicate proportion daylight category of each 6 h period (black triangles = night, dark gray = mostly night, light gray = mostly day, white = day, as defined in Results section *Links to Mesoscale Circulation and Coastal Topography*). (C) Histograms of seal 626's diving, one for each proportion daylight category, showing number of dives within each dive tag pre-programmed depth bin. Depth bin bounds are indicated on the left y-axis by whiskers. Green line in panel C is a histogram of MLD observations collected >175 km from the shelf break using 5 m depth bins. Filled triangles to the left of Panel C indicate the average dive depth in each category of proportion daylight and the average MLD.

Two individuals (female seals 626 and 628) recorded brief bouts of area-restricted search movement near Seaglider transects as the females made their way northward along the OR and WA coasts in late spring and early summer 2008. Female 626 was first to arrive in the WA area in late April, making her way northward roughly 200 km from the shelf break and parallel to the coast while slowing or stopping in irregular fashion, presumably to forage (Fig. 14A). On 28 April, she slowed movement near 46.8°N, 127.5°W, within 50 km of the offshore end of the Seaglider 101 (SG101) transect (Fig. 14A). She departed to the north a week later, crossing 47°N at 127.42°W on 6 May. At that time SG101 was heading offshore from the shelf break along 47°N and crossed 127.42°W on 7 May at 1900 UTC. The mapped SG101 data for the transect (Fig. 14B) show moderate concentrations of Chla and MLDs that had begun to shoal from the winter maximum to 30–40 m. Female 626's diving during 6 h periods classified as day (mean = 29.6 m, n = 391; Fig. 14C) showed correspondence with the MLD measured by SG101 (mean = 32.7 m of n = 19 profiles collected >175 km from shelf break). Night dives (black) were more numerous and shallower (mean = 16.6 m, n = 1603; Fig. 14C). The partition of diving effort (night/day dives ratio = 4.1) is more typical of diving in the in the offshore migratory transit period in the NP, while the correspondence between day dives and the depth of the mixed layer is typical of statistical results in the CC.

**Figure 15:**
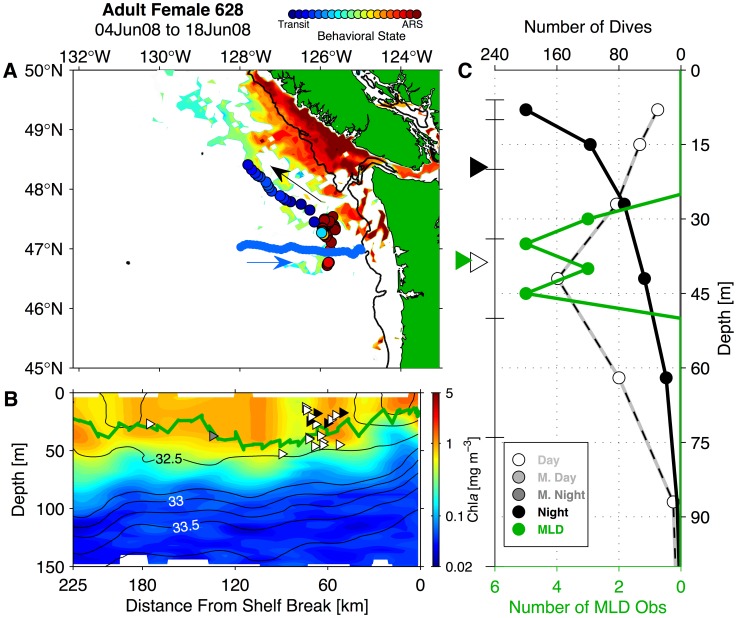
Adult female northern fur seal 628 near Seaglider 101 (SG101), 4 June to 18 June 2008. (A) Estimated locations of female 628 (filled color circles) and reported GPS locations of SG101 (thick blue line) off the Washington and British Columbia coast (green). Colors of filled circles indicate behavioral state (scale at top, ARS = area-restricted search). The 200 m isobath is indicated by thin black line. Arrows indicate direction of travel of 628 and SG101. Background colors in panel A indicate surface chlorophyll-*a* (Chl*a*) in cloud-free regions on 13 June 2008, plotted according to the color scale in panel B. (B) Upper-ocean conditions by depth and distance from the shelf break recorded by SG101 during its crossing: Chl*a* (background colors), salinity (black contours), and mixed-layer depth (MLD, thick green line). Triangles indicate average depths of female northern fur seal 628's dives in each 6 h period. Triangles are plotted by cross-shore distance along the Seaglider line, and triangle fill colors indicate proportion daylight category of each 6 h period (black triangles = night, dark gray = mostly night, light gray = mostly day, white = day, as defined in Results section *Links to Mesoscale Circulation and Coastal Topography*). (C) Histograms of seal 628's diving, one for each proportion daylight category, showing number of dives within each dive tag pre-programmed depth bin. Depth bin bounds are indicated on the left y-axis by whiskers. Green line in panel C is a histogram of MLD observations collected between 50 and 100 km from the shelf break using 5 m depth bins. Filled triangles to the left of Panel C indicate the average dive depth in each category of proportion daylight and the average MLD.

Female seal 628 arrived in May, a month after 626, and beginning 6 June spent a week near 47.5°N, 125.75°W, 50 km north of the inshore portion of the SG101 transect, before departing to the northwest on 11 June (Fig. 15A). Female seal 628 spent nearly 200 hours in an area 60–75 km from the shelf break and the movement model indicated area-restricted search behavior within this zone (Fig. 15A). Seaglider 101 was near the offshore waypoint of the its navigation pattern when 628 first arrived, and subsequently headed inshore along 47°N, crossing 125.75°W on 14 June, three days after 628's departure. Chlorophyll-*a* concentrations were moderate and mesoscale eddies were absent. Seaglider 101 sampled the edge of a freshwater plume as the vehicle reached the shelf break (Fig. 15B) where the MLD shoaled to 20 m depth. Female 628 foraged about 20 km west from the edge of this plume. Day dives (n = 419) were nearly as numerous as night dives (n = 462). The vertical distribution of day diving (mean = 38.7 m) of female 626 was centered on the MLD (mean = 38.3 m of n = 16 profiles between 50 and 100 km from the shelf break), though with a broader vertical range (Fig. 15C). Night dives were surface-intensified and mostly shallower than the MLD (mean = 19.59 m).

#### Columbia River Plume

**Figure 16:**
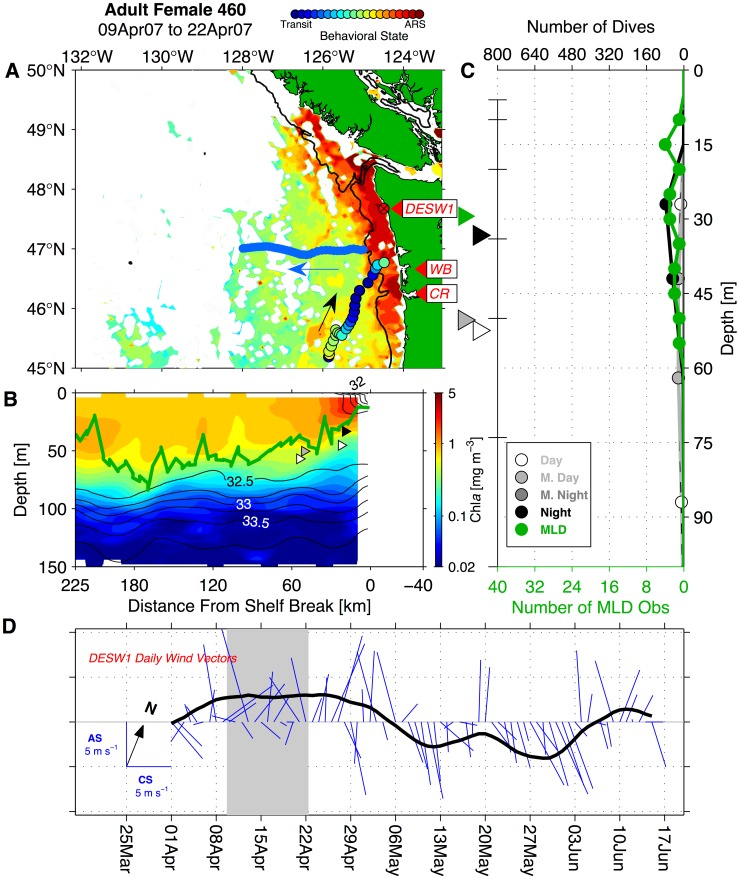
The initial approach of adult female northern fur seal 460 to Seaglider 014 (SG014), 9 April to 22 April 2007 (1 of 4). (A) Estimated locations of female 460 (filled color circles) and reported GPS locations of SG014 (thick blue line) off the Washington and British Columbia coast (green). Red labels indicate the Columbia River mouth (CR), Willapa Bay coastal inlet feature (WB), and the location of National Data Buoy Center station Destruction Island (DESW1, red cross-circle at station). Colors of filled circles indicate behavioral state (scale at top, ARS = area-restricted search). The 200 m isobath is indicated by thin black line. Arrows indicate direction of travel of 460 and SG014. Background colors in panel A indicate surface chlorophyll-*a* (Chl*a*) in cloud-free regions on 22 April 2007, plotted according to the color scale in panel B. (B) Upper-ocean conditions by depth and distance from the shelf break recorded by SG014 during its crossing: Chl*a* (background colors), salinity (black contours), and mixed-layer depth (MLD, dark green line). Triangles indicate average depths of female northern fur seal 460's dives in each 6 h period. Triangles are plotted by cross-shore distance along the Seaglider line, and triangle fill colors indicate proportion daylight category of each 6 h period (black triangles = night, dark gray = mostly night, light gray = mostly day, white = day, as defined in Results section *Links to Mesoscale Circulation and Coastal Topography*). (C) Histograms of seal 460's diving, one for each proportion daylight category, showing number of dives within each dive tag pre-programmed depth bin. Depth bin bounds are indicated on the left y-axis by whiskers. Green line in panel C is a histogram of MLD observations collected between 0 and 60 km from the shelf break using 5 m depth bins. Filled triangles to the left of Panel C indicate the average dive depth in each category of proportion daylight and the average MLD. (D) Daily-averaged wind vectors at 52.7 m height obtained from DESW1. Wind measurements at DESW1 have been rotated into an alongshore (AS)/cross-shore (CS) coordinate system. In panel D, the solid black line is a 10 d running average of the poleward component of wind. Gray shading indicates the time period covered by panels A–C.

**Figure 17:**
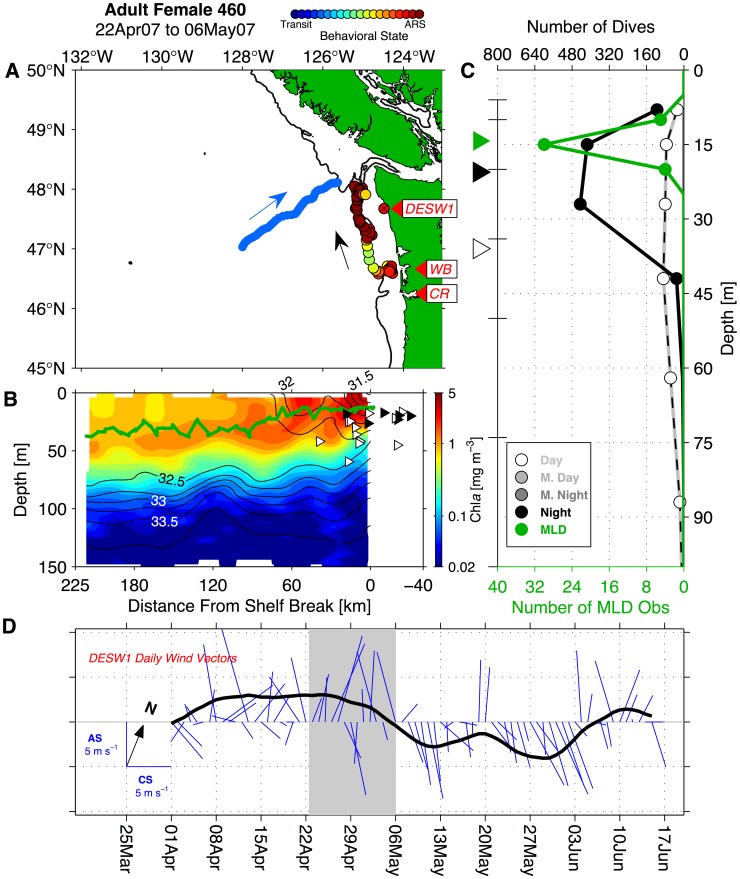
Adult female northern fur seal 460 in area-restricted search off the Washington coast, continued: 22 April to 06 May (2 of 4). (A) Estimated locations of female 460 (filled color circles) and reported GPS locations of Seaglider 014 (SG014, thick blue line) off the Washington and British Columbia coast (green). Red labels indicate the Columbia River mouth (CR), Willapa Bay coastal inlet feature (WB), and the location of National Data Buoy Center station Destruction Island (DESW1, red cross-circle at station). Colors of filled circles indicate behavioral state (scale at top, ARS = area-restricted search). The 200 m isobath is indicated by thin black line. Arrows indicate direction of travel of 460 and SG014. A sufficiently cloud-free surface chlorophyll-*a* (Chl*a*) image was not found for this time period. (B) Upper-ocean conditions by depth and distance from the shelf break recorded by SG014 during its crossing: Chl*a* (background colors), salinity (black contours), and mixed-layer depth (MLD, dark green line). Triangles indicate average depths of female northern fur seal 460's dives in each 6 h period. Triangles are plotted by cross-shore distance along the Seaglider line, and triangle fill colors indicate proportion daylight category of each 6 h period (black triangles = night, dark gray = mostly night, light gray = mostly day, white = day, as defined in Results section *Links to Mesoscale Circulation and Coastal Topography*). (C) Histograms of seal 460's diving, one for each proportion daylight category, showing number of dives within each dive tag pre-programmed depth bin. Depth bin bounds are indicated on the left y-axis by whiskers. Green line in panel C is a histogram of MLD observations collected between 0 and 60 km from the shelf break using 5 m depth bins. Filled triangles to the left of Panel C indicate the average dive depth in each category of proportion daylight and the average MLD. (D) Daily-averaged wind vectors at 52.7 m height obtained from DESW1. Wind measurements at DESW1 have been rotated into an alongshore (AS)/cross-shore (CS) coordinate system. In panel D, the solid black line is a 10 d running average of the poleward component of wind. Gray shading indicates the time period covered by A–C.

**Figure 18:**
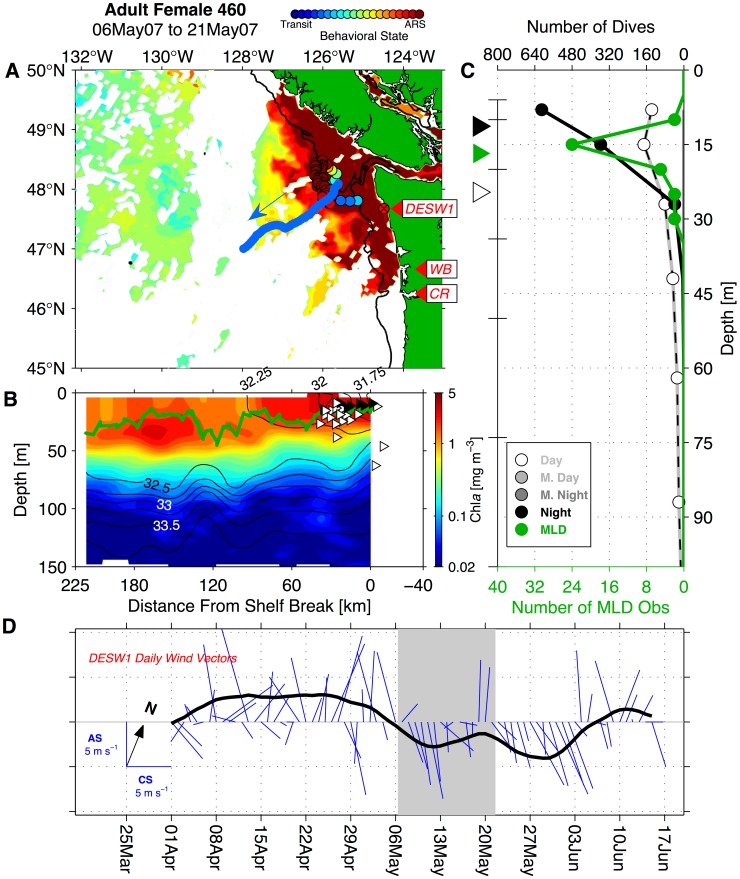
Adult female northern fur seal 460 in area-restricted search off the Washington coast, continued: 06 May to 21 May 2007 (3 of 4). (A) Estimated locations of female 460 (filled color circles) and reported GPS locations of Seaglider 014 (SG014, thick blue line) off the Washington and British Columbia coast (green). Red labels indicate the Columbia River mouth (CR), Willapa Bay coastal inlet feature (WB), and the location of National Data Buoy Center station Destruction Island (DESW1, red cross-circle at station). Colors of filled circles indicate behavioral state (scale at top, ARS = area-restricted search). The 200 m isobath is indicated by thin black line. Arrow indicates direction of travel of SG014. Background colors in panel A indicate surface chlorophyll-*a* (Chl*a*) in cloud-free regions on 11 May, plotted on the same scale as in panel B. (B) Upper-ocean conditions by depth and distance from the shelf break recorded by SG014 during its crossing: Chl*a* (background colors), salinity (black contours), and mixed-layer depth (MLD, dark green line). Triangles indicate average depths of female northern fur seal 460's dives in each 6 h period. Triangles are plotted by cross-shore distance along the Seaglider line, and triangle fill colors indicate proportion daylight category of each 6 h period (black triangles = night, dark gray = mostly night, light gray = mostly day, white = day, as defined in Results section *Links to Mesoscale Circulation and Coastal Topography*). (C) Histograms of seal 460's diving, one for each proportion daylight category, showing number of dives within each dive tag pre-programmed depth bin. Depth bin bounds are indicated on the left y-axis by whiskers. Green line in panel C is a histogram of MLD observations collected between 0 and 60 km from the shelf break using 5 m depth bins. Filled triangles to the left of Panel C indicate the average dive depth in each category of proportion daylight and the average MLD. (D) Daily-averaged wind vectors at 52.7 m height obtained from DESW1. Wind measurements at DESW1 have been rotated into an alongshore (AS)/cross-shore (CS) coordinate system. In panel D, the solid black line is a 10 d running average of the poleward component of wind. Gray shading indicates the time period covered by A–C.

**Figure 19:**
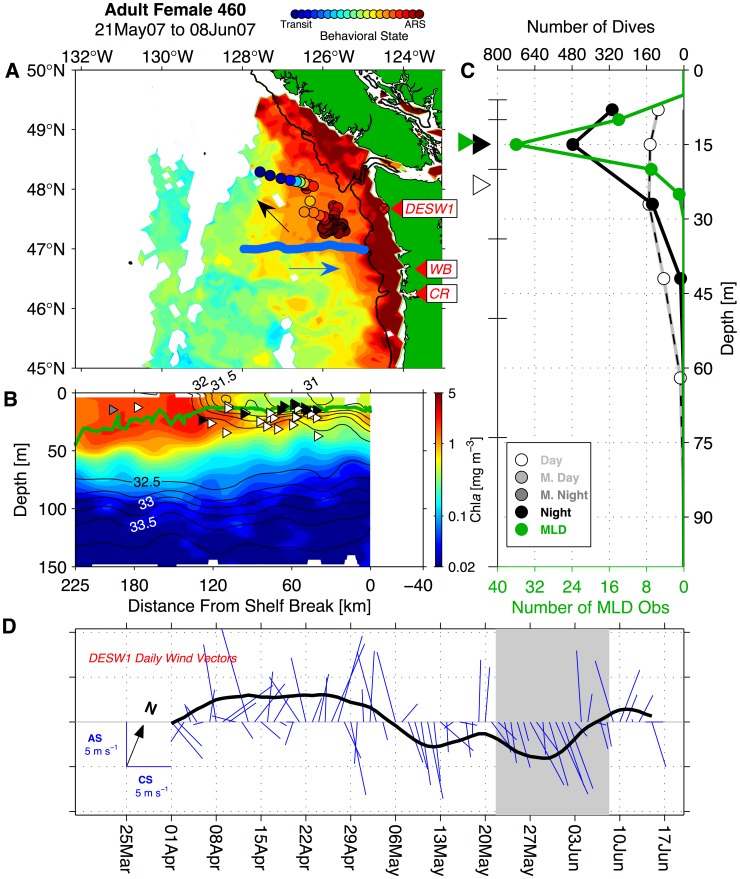
Adult female northern fur seal 460 in area-restricted search and departure from the Washington coast: 21 May to 08 June (4 of 4). (A) Estimated locations of female 460 (filled color circles) and reported GPS locations of Seaglider 014 (SG014, thick blue line) off the Washington and British Columbia coast (green). Red labels indicate the Columbia River mouth (CR), Willapa Bay coastal inlet feature (WB), and the location of National Data Buoy Center station Destruction Island (DESW1, red cross-circle at station). Colors of filled circles indicate behavioral state (scale at top, ARS = area-restricted search). The 200 m isobath is indicated by thin black line. Arrows indicate direction of travel of 460 and SG014. Background colors in panel A indicate surface chlorophyll-*a* (Chl*a*) in cloud-free regions on 29 May 2007, plotted according to the color scale in panel B. (B) Upper-ocean conditions by depth and distance from the shelf break recorded by SG014 during its crossing: Chl*a* (background colors), salinity (black contours), and mixed-layer depth (MLD, dark green line). Triangles indicate average depths of female northern fur seal 460's dives in each 6 h period. Triangles are plotted by cross-shore distance along the Seaglider line, and triangle fill colors indicate proportion daylight category of each 6 h period (black triangles = night, dark gray = mostly night, light gray = mostly day, white = day, as defined in Results section *Links to Mesoscale Circulation and Coastal Topography*). (C) Histograms of seal 460's diving, one for each proportion daylight category, showing number of dives within each dive tag pre-programmed depth bin. Depth bin bounds are indicated on the left y-axis by whiskers. Green line in panel C is a histogram of MLD observations collected between 0 and 60 km from the shelf break using 5 m depth bins. Filled triangles to the left of Panel C indicate the average dive depth in each category of proportion daylight and the average MLD. (D) Daily-averaged wind vectors at 52.7 m height obtained from DESW1. Wind measurements at DESW1 have been rotated into an alongshore (AS)/cross-shore (CS) coordinate system. In panel D, the solid black line is a 10 d running average of the poleward component of wind. Gray shading indicates the time period covered by A–C.

Freshwater outflow from the Columbia River is another important source of mesoscale variability in the WA coastal transition zone that influences NFS foraging. This is illustrated by Seaglider data taken in spring 2007, during which time female 460 foraged from the outer shelf to the outer slope between GH and CF (Figs. 16–19; for track overview see Fig. 11A). Female 460 spent the most time in close proximity to the Seaglider transects of any satellite-tagged individual equipped with a dive recorder. She arrived at 47°N on 21 April 2007 and departed from the CF line on 5 June, moving seaward and generally northward while in the Seaglider area. During this time Seaglider 014 (SG014) made four cross-shore transects.

As female 460 transited from pelagic waters off the OR coast toward the WA shelf in mid-April, SG014 performed a transit along the GH line (Fig. 16A). During this time, late-winter conditions prevailed. Seaglider 014 results along the GH transect line reflect moderate Chl*a* concentrations and a 50 m MLD offshore (Fig. 16B). During this time, seal 460 was mostly in a transitory state and performed few dives to depths at or below the MLD (Fig. 16B–C). Seaglider 014 also crossed a portion of a low-salinity freshwater plume that was straddling the shelf edge. This is evident in the narrow 15 km low-salinity band that was found at the surface just off the shelf break, with a shallow MLD and elevated concentrations of Chl*a* (Fig. 16B). The plume's cross-shore position was consistent with the alongshore component of surface winds observed at DESW1 (47.675°N, 124.485°W), an island on the WA inner shelf between GH and CF. From late March to mid-April winds were strong and predominantly poleward (Fig. 16D), typical of late-winter winds off WA prior to a shift to upwelling-favorable winds known as the “spring transition” [78]. Under late-winter conditions, frictional surface currents, background shelf flow, and buoyancy-driven geostrophic currents keep the Columbia River plume over the shelf and in a meridional orientation extending northward from the mouth of the Columbia estuary [78]–[82]. Female 460 moved onto the WA shelf around 22 April, and, roughly coincident with her encounter of the plume, began foraging outside the mouth of Willapa Bay before moving northward parallel to the shelf break (Fig. 17A).

The next SG014 transect, taken from 22 April to 6 May, showed the plume off CF with low salinity and elevated Chl*a* concentrations at depths less than 25 m and within 60 km of the shelf break (Fig. 17A–B). During this time, 460 spent most of her hours in an area over the shelf break and within the plume. Behavioral state estimates during this time indicate area-restricted search. The number of total dives increased substantially (n_day_ = 349; n_night_ = 1011). The salinity-stratified MLD was shallow enough that both day (mean = 36.0 m) and night dives (mean = 20.6 m) were at or slightly below the MLD (mean = 14.3 m of 39 profiles within 60 km of the shelf break; Fig. 17B–C). The alongshore component of wind from 22 April to 6 May remained poleward (Fig. 17D) but began to relax on 7 May, after which followed an abrupt, sustained reversal to equatorward winds (Fig. 18D). Shifts to equatorward (upwelling) winds, following poleward (downwelling) winds, advect low-salinity plume water westward and southward offshore from the WA shelf. As a result, SG014's return trip offshore along the CF line from 6 May to 21 May observed the plume offshore extent increase to roughly 100 km from the shelf break at its maximum (Fig. 18A–B). It is also possible that freshwater outflow from the Strait of Juan de Fuca contributed to freshwater in the plume off CF. Chlorophyll-*a* concentrations within the plume were still elevated relative to surface concentrations offshore and during this time female seal 460's foraging effort was the most spatially concentrated, contained entirely within 50 km of the shelf break and 50 km from the Seaglider survey (Fig. 18A–B). Foraging was offshore in comparison to the previous time period, roughly tracking the plume's movement. Day diving (mean = 24.6 m, n = 493) was mostly to depths near the MLD but with some diving averaging 30–75 m below (Fig. 18C). Night diving (mean = 11.3 m, n = 936) was more vertically concentrated and some night diving extended to the MLD, which deepened in the plume between early and mid-May (mean MLD = 16.8 m of 35 profiles within 60 km of the shelf break; Fig. 18B). During this time, MLDs began to shoal offshore and a subsurface Chl*a* maximum outside of the stratified freshwater layer developed (150–200 km from the shelf break; Fig. 18B).

From 21 May onwards, female NFS 460 continued foraging within the plume, tracking southward and away from the shelf break while in between the two Seaglider transect locations, before departing around 5 June (Fig. 19A). Seaglider 014 returned to the GH line from 21 May to 8 June and recorded very low concentrations of Chl*a* within the freshwater layer, which indicated that the bloom there was depleted (Fig. 19B). In contrast, Chl*a* concentrations were high offshore, where the spring bloom had just begun. Persistent southward winds, driving near-surface frictional currents, had by this time advected the Columbia River plume offshore with its edge 125 km from the shelf break (Fig. 19B, D). Female 460's final foraging effort fell within this region before she departed to the northwest (Fig. 19A). Consistent with late-season GAM results for depth of diving, 460's day dives (mean = 23.1 m, n = 507) were concentrated ∼10 m deeper than the MLD (mean MLD = 14.5 m, n = 58 profiles collected between 25 and 150 km from the shelf break; Fig. 19B–C). Night dives (mean = 15.0 m, n = 936) were concentrated near the MLD and more vertically localized than day dives (Fig. 19B–C).

## Discussion

The timing of migration and the alongshore and cross-shore distribution of female NFS observed in this study are largely consistent with previous studies derived from pelagic scientific takes or anecdotal knowledge. Historical data indicate that after transiting the Aleutian passes that connect the Bering Sea and NP, female NFS cross the NP and arrive on the North American west coast beginning in late November [1], [4], [23], [52]. Pregnant individuals consistently return to the Pribilof Islands in late June/early July [83], and in the intervening months, analysis of pelagic sampling suggested that concentrations of seals were highest off California in early winter with peak numbers shifting gradually northward as the overwintering period progressed [52], [83], [84]. The monthly patterns of alongshore distribution in this study exhibit both characteristics of this migratory pattern. The cross-shore distribution also supports the observations of Kajimura et al. [85], who noted that scientific pelagic collections found females preferentially concentrated within a 60 km band off the shelf break and over the continental slope, likely due to abundant food resources and/or increased prey availability in this area.

Daytime diving patterns of females in the CC ecosystem suggested that the MLD influences the depth of these dives. Generalized additive model results indicated that female NFS average daytime diving depths in 6 h periods tracked the winter-summer shoaling of the MLD, but with differing offsets and character between early season (day diving shallower than MLD) and late season (day diving highly concentrated and 5–10 m below MLD). Though the female average dive depths used as a response variable in the GAM were not necessarily spatially co-located with Seaglider measurements, with the exception of the presence of freshwater plumes, the Seaglider average winter-summer evolution of MLD is similar to that in other locations in the coastal CC and southern GA (e.g., Fig. 17 of [86]). Three adult females who encountered Seagliders in the WA coast region in April, May, and June dived to or slightly deeper than the MLD during the daytime, while night dive depths remained shallow and were not affected by the MLD, with the possible exception of diving within the Columbia River plume, where the MLD is very shallow (10–20 m).

These dive patterns are likely associated with the diel migration of typical fur seal prey in coastal transition zone habitats. Diet analysis of the scientific pelagic catch concluded that off WA, OR, and BC, NFS are opportunistic foragers that feed on a wide range of species, including various squids (market squid *Loligo opalescens*, also *Gonatus* spp., *Onychoteuthis* spp., especially important off the shelf), northern anchovy (*Engraulis mordax*), Pacific hake (*Merluccius productus*), rockfish (*Sebastes* spp.) and Pacific herring (*Clupea pallasii*) [1], [31]. Juveniles of the latter species feed in the Strait of Juan de Fuca and Hecate Strait where they are preyed upon by salmon (*Oncorhynchus* spp.), which in turn are also fur seal prey. All of these species have been observed undertaking diel vertical migrations, feeding near the surface at night (corresponding to shallow nighttime female dive depths in the CC) and moving deeper during the day. Female diving behavior observed in this study indicates that during daylight hours, some prey species must congregate in the high-stratification transition layer immediately below the well-mixed surface layer rather than in the deep scattering layer at depths of 100 m or below. This places them within the vertical range of adult female physiological diving capabilities.

Ecosystem effects in the GLMM results for number of dives per 6 h period reflect the differing environmental conditions that seals encounter as well as the time of year at which each ecosystem is occupied. Fewer dives in the BS ecosystem at night is a similar result to [25] and likely reflects adult females rapidly exiting the Bering Sea and making their way to the CC and GA ecosystems. The interaction between proportion daylight and the CC ecosystem showed that as females moved closer to the coast, daytime dives represented a greater percentage of total diving effort. This resulted in a nearly even distribution of diving versus proportion daylight in the CC ecosystem. Sterling et al. [25] tracked a single female NFS who entered the CC in one overwintering period, and this individual showed an increase in day diving after entering the CC, though it was unclear in their results if this was an ecosystem response or simply individual variability. The results presented here for number of dives versus proportion daylight per 6 h period confirm that this is a consistent behavioral response to ecosystem traits, present in multiple individuals across multiple years. This is contrary to the pelagic behavior displayed in the NP ecosystem and in previous studies where adult females concentrate most of their effort in shallow, night or crepuscular diving [1], [25], [29], [55], [87]–[92]. Diving behavior of adult females in the CC in this study is, instead, similar to adult male diving behavior expressed during overwintering in the northern NP, where seals dived mostly during the daytime and to the MLD and below [25].

The MLD in the northern NP is deeper (100–125 m) in winter when compared to the MLD in the Seaglider survey region in winter (20–75 m; [77], [93], this study). The majority of female overwinter habitat utilization was concentrated within 150 km of the shelf break, and Seaglider results demonstrate that MLD in this region was 60–70 m on average in mid-winter and rarely exceeded 80 m (Figs. 9–10). If the MLD represents the minimum depth of daytime seal prey fields, the deeper winter MLD in the NP implies that daytime prey fields are located deeper in the water column there than in the CC. The dive duration constraint of female fur seals, owing to their smaller mass, may mean that in the central and northern NP, the depth of winter daytime prey fields could place them outside the aerobic diving capabilities of the smaller females or require energy expenditure and recovery times that are disadvantageous compared to foraging closer to the shelf break. Thus, the cross-shore variability of the Pacific Ocean winter MLD could lead to a lateral gradient in accessibility of daytime prey fields. In the coastal ecosystem, wind-driven upwelling and subsequent productivity creates an abundance and diversity of prey [30]. The diving behavior observed in this study indicates that some of these prey species are localized at shallower depths where female physiology allows access at all times of day. This is consistent with the hypothesis that the location of female overwinter foraging grounds is dictated by interactions between prey availability and fur seal physiology [25], [94].

The greater proportion of dives during daytime in the CC habitat resulted in fewer total dives in the CC LME. The rest periods required between dives increase with dive depth for adult female NFS [88]. As a result, the rate of dives per hour decreases nonlinearly with increasing dive depth. Costa and Gentry [95] compared the energy budgets of deep- and shallow-diving adult females in the Bering Sea and found that although deep divers captured less total prey biomass, their net energy storage and mass gain was comparable to shallow divers over the duration of a foraging trip, due to less total energy expended in deep diving and, presumably, a higher energy content of their prey. Thus an increase in foraging efficiency (net energy stored vs. foraging metabolism) in deeper dives offsets the lower rate at which they occur. Some of these same factors may also be important for overwinter foraging of adult females in the CC, although the depth difference between deep day dives versus shallow night dives in this study (e.g., 30–60 m deep vs. 10–20 m shallow) was less than day/night differences in the Bering Sea (where day dive depths are 60–100 m; [89], [95]). Whether prey energy content in the CC LME differs between near-surface and at the base of the mixed layer, and whether day and night prey fields are comprised of different species or age class composition within a species, is unknown. This may be unimportant in late migration season or in the Columbia River plume, where MLDs are shallow enough that if prey aggregate just below the mixed layer during daytime, they are accessible by dives of only 20–25 m depth as shown in Fig. 18B.

Goebel et al. [89] concluded that aside from prey size and energy density, ease of capture is also another important factor that influences foraging energetics, and this could also partially explain the shift to day diving with continental slope habitat and season. Daytime foraging, especially below the shallow MLD off WA in late spring and early summer, may offer some advantage in visual identification of prey and increase the foraging success of adult females [96]. Increased spatial density of schooling fish in the daylight hours (e.g., [97]–[99]) may also be a contributing factor that adds to ease of capture and thus a potentially greater assimilated energy per dive in deeper daytime bouts. An increase in feeding event size during daytime, suggestive of greater assimilated energy per dive, has been observed in free-ranging adult female gray seals (*Halichoerus grypus*) in the Atlantic Ocean although the reasons for this are unclear [100].

The GLMM results for behavioral state are consistent with Sterling et al. [25] and show that surface wind speed is an important driver of behavioral state. Animals experience stronger winds while in transit during the early migratory period in the central NP and GA, and additionally respond to strong winds in the destination ecosystems by shifting away from area-restricted search towards transitory behavior. The relationship between day dive depths and the MLD observed in this study suggests a mechanism by which strong surface winds could reduce prey accessibility, and thus influence behavioral state, since these winds can force convection and deepen the MLD. The seasonal progression of behavioral state from low indices (transit) to high (area-restricted search) reflects females transiting quickly through the Aleutian passes and across the open NP, which is consistent with historical understanding of NFS migratory patterns. The interaction between surface wind speed and season as a predictor of behavioral state is a new result, reflecting a reduced effect of surface wind speed on behavior in the late spring and early summer.

A key qualitative result of this study, consistent with other analyses of both the NFS overwintering period and summer foraging, is evidence of adult female behavioral cues in response to eddies and energetic surface circulation as revealed by satellite altimetry. Females demonstrated movement alignment with geostrophic surface currents [1] and area-restricted search within and around eddies, or within strong jets forming the boundary between adjacent features [24], [25], [39], [40]. On aggregate, females spent twice as much time (as a fraction of total time within the ecosystem) within and near (20 km from approximate edge) altimetry-identified coherent mesoscale eddies in the CC than during transit in the NP. This effect was magnified for long-duration tracks, which may relate to increased eddy generation late in the overwintering season as upwelling winds induce a strong coastal jet, which becomes unstable and increases EKE values near the coast [101]. Adult female foraging in the eddy-rich margins of energetic boundary currents is similar to that observed for males exiting the Bering Sea in fall and winter [24]. The distribution of female habitat utilization relative to eddies showed increased probability density of habitat utilization near 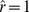 for area-restricted search locations relative to transit. For an axially symmetric eddy identified in the Chelton et al. [63] time series, 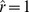 corresponds to the radial station of maximum velocity regardless of azimuthal station (compass point) around the eddy center. It is important to note when considering the eddy results that many individual eddies demonstrate substantial asymmetry or ellipticity (e.g., Fig. 12); thus, the description of the aggregate physical environment encountered by females at 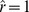 is only an approximate one.

The reason(s) underlying female foraging responses within and near some eddies are unknown, but may relate to physical processes near these features that alter prey characteristics in such a way as to make them energetically advantageous foraging hotspots. Benoit-Bird et al. [102] showed that in the Bering Sea in summer, the vertical distribution of aggregate prey patches and density within those patches were strong predictors of NFS habitat utilization. As noted above, vertical distribution affects the required energy expenditure per individual dive, while within-patch density affects the efficiency of prey capture during a dive. There are a variety of ways in which eddies or fronts could lead to shallower prey vertical localization or increased density within prey patches. For example, Godø et al. [103] hypothesized that grazing zooplankton align themselves with the strong currents at the eddy edge in order to optimize their foraging environment, and that mesopelagic forage fish cue on these resources through a variety of techniques, including passive displacement by background currents. Godø et al. [103] report observations of biomass accumulation near an eddy edge during periods of negligible phytoplankton community growth, suggesting that the mechanisms that make eddies a favorable environment for forage fishes are independent of any boost in primary productivity. This is consistent with female foraging in this study within eddies in January–March, when overall productivity in the CC is low and more spatially confined to the nearshore region [104]. It is also possible that NFS prey that prefer a narrow thermal range may simply avoid eddy core regions due to the contrast in temperature between an eddy cores and surrounding water, leading to apparent aggregation at the edge as an eddy moves through a uniform density of NFS prey. At ocean fronts, mixed-layer instabilities act to restratify the upper water column [105], thus reducing the MLD and potentially influencing prey vertical localization.

Elevated phytoplankton biomass at eddy edges or within frontal features could act as a secondary driver by creating a bottom-up forcing of forage fish concentrations over longer time scales, especially late in the upwelling season. Strong wind-driven vertical velocities near the region of maximum velocity at an eddy edge [106], or along the axis of a jet [107], increase the vertical transport of growth-limiting nutrients to the euphotic zone, and several studies have found elevated primary productivity, zooplankton, and fish populations associated with eddies and fronts in the CC [108]–[112]. This mechanism is consistent with the observed increase in habitat utilization near 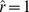 for area-restricted search behavior. In the northern GA, eddies also play an important role in cross-shelf exchange and horizontal stirring of shelf and offshore waters in which phytoplankton growth is limited by different nutrients [113]–[118], thus also potentially leading to an increase in primary productivity relative to background conditions. The stirring of nutrients at the eddy edge, or redistribution of productivity around the rim of any eddy impinging on the shelf (independent of any enhancement), could in part account for NFS cueing on the outer rim of eddies in the GA [119]. A secondary benefit of eddies may be energetic savings due to swimming with eddy currents during either phase of the migration [1].

Given the variety of possible mechanisms involved, there are likely spatial and temporal differences between the migratory ecosystems regarding which factors are most important in affecting eddy-related foraging, and indeed whether an individual eddy would be a profitable foraging area. It should be noted that females avoided many features, and that the observed difference in median 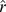 value with behavioral state was not statistically significant for the entire dataset (though it was significant when considering only tracks >200 d, see Fig. S5). Furthermore, despite the qualitative evidence of alignment, efforts to quantify eddy statistical effect on behavioral state by an EKE term were unsuccessful, though EKE did not significantly degrade model performance/variance explained (not shown). This may be due to several factors. Migratory fur seals bypassed some high-EKE features while concentrating their effort in others, indicating that there may be other covariates that interact with EKE, such as subsurface water column structure, that we may not be able to measure in this analysis. Furthermore, mean eddy kinetic energy varies strongly with latitude in the eastern Pacific [120], even though EKE as a fraction of mean energy remains large [121]. Thus, an animal foraging near 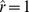 off the WA or BC coast (e.g., Fig. S3) may be in a region of EKE that is locally elevated but weak relative to the overall dataset. Finally, migratory momentum and strong winds likely also play a role in females bypassing high EKE features during their transit across the NP ecosystem [25], [122].

Despite the lack of consistent response, evidence of some female foraging within mesoscale variability in this and other studies suggests an additional driver of increased concentration of female effort within the inner coastal transition zone and near topographic features. In the CC eastern boundary current system, interaction between alongshore flow and topography occurs near canyons, sharp turns in the continental slope, and headlands including Cape Mendocino, Cape Blanco, and Point Sur. The resulting meanders in the coastal jet are unstable and produce eddies, jets, and fronts [30], [86], [101], [123]–[127]. Eddy kinetic energy in these regions is highest during the upwelling season, but altimetry studies indicate that there is still significant EKE in the winter months, located at a greater distance from shore than in spring and summer [101], [127]. Figure 12 illustrates that eddy generation processes are still active during winter at Cape Blanco. By the mechanisms outlined above, regions of enhanced eddy activity may also be regions with a greater likelihood of favorable foraging conditions for adult female NFS. Spatial density of eddy generation and mean EKE are greatest within 100–200 km of the shelf break in the CC and GA ecosystems [63], [120], [128], [129] and this may be an additional driver of the observed cross-shore distribution of adult female habitat utilization beyond regional differences in prey accessibility. Aside from headlands in the central CC and the Columbia River, females congregated near or offshore of Queen Charlotte Sound, formation site for Haida eddies [130], and the entrance to the Strait of Juan de Fuca – habitat for juvenile herring (*C. pallasi*) and salmon (*Oncorhynchus* spp.) on which adult female NFS feed [31], [131]. The persistent and productive Juan de Fuca eddy also appears annually at the mouth of the Strait, though after the spring transition [132]. This is near the time of year when females are probably beginning their return to Bogoslof and St. Paul Islands.

It should be noted that both the alongshore distributions of NFS derived from historical pelagic data [4] and the distribution of female NFS in this study suffer from potential biases. For example, at-sea takes of females from early pelagic sealing were largely weather-dependent and generally restricted to more coastal habitats. Pelagic scientific collections from 1958–74 were conducted with a requirement that each participant nation obtain a minimum quota; as a result, observational effort was concentrated in specific portions of their range that females were known to inhabit based on historical evidence. In this study, satellite tag longevity and retention effects likely reduced the overall number of adult female seal-hours in the late-season months, probably contributing to the apparent departure of females from the region in May–June (Fig. 5E–F). Nonetheless, the distribution of females shows good qualitative agreement with previous understanding of migratory patterns in the coastal region.

The multi-week record of female NFS 460's WA coast foraging in the spring and early summer of 2007 is the first direct evidence of the Columbia River plume as a feature on which female NFS cue to find prey. Female 460's movements tracked within the plume edge and core, moving away from the shelf break along with the plume after a switch to equatorward winds, and also departing at roughly the same time as bloom exhaustion within the plume (although the latter could be purely coincidental). In spring, off the WA coast, the plume serves as an effective lateral transporter of biomass offshore as plume orientation shifts with the alongshore winds [81], [133]. Additionally, a key element of the plume is the low-salinity “cap” it places on the water column [80], [82]. Its strong salinity stratification reduces the size of turbulent vertical overturns and leads to a shallow MLD, potentially increasing the accessibility of daytime prey fields for adult females encountering the plume off the WA coast.

These results provide further support to the idea that as top predators, NFS respond to integrated biological changes cascading upward from the ocean physical environment through the marine ecosystem. In this study, eddies and the Columbia River plume represent sources of mesoscale variability that elicited strong behavioral responses in mature, experienced individuals, underscoring that adult females are highly flexible in altering their foraging intensity in response to ephemeral prey resources encountered during the overwintering phase [90]. At longer scales, seasonal alterations in the character and intensity of adult female NFS diving likely reflect seasonal changes in surface winds and heating, ecosystem phenology, and prey movements in the CC and GA. Females exhibited a shift in character in day dives, towards vertically concentrated foraging below the MLD, that occurred roughly in tandem with the spring shoaling of the MLD in late April and early May. This timing is coincident with the onset of increases in body size (aggregate body weight and length) in adult females collected in historical pelagic sampling and emphasizes that the processes discussed in this manuscript can potentially exert a fundamental control over adult female NFS condition during the overwintering period [134]. The association of day diving depths with the MLD suggests that short-term physical processes such as storms and atmospheric variability can influence vertical localization of NFS prey. The MLD is highly dynamic during fall in the northeast Pacific, where seasonal deepening is episodic, driven by individual storms that force convection and can increase the MLD by 10 m over a 1–2 d span [135], [136]. Experienced adults contend with these biophysical alterations but it is unknown if and how event or seasonal scale biophysical variation could impact the condition of less physiologically capable juveniles or pups, and ultimately NFS demography by affecting overwinter survival. Several studies have reported numerous tagged NFS pup carcasses recovered along the coasts of OR, WA, and BC following heavy weather in the northeast Pacific [137]–[140]. Processes described in this study support the hypothesis that a deeper dispersed prey field altered by winter processes could disproportionately affect smaller, foraging-naive pups or juveniles lacking the physiological diving capabilities of larger adults [55], and warrant further investigation.

## Conclusions

Comparison of NFS behavior to remotely-sensed oceanographic fields, both surface and subsurface, informs our understanding of how oceanographic parameters affect individuals or groups of individuals. This is an important step in exploring both the long-term role of a variable environment on a widely-distributed top marine predator as well as the mechanisms potentially underpinning its population decline. The results of this study demonstrate that in the pelagic environment off WA and in the CC ecosystem, adult female NFS exhibit a shift in their foraging effort towards daylight hours and that foraging preferentially occurs in a cross-shore band with its center 60–80 km from the shelf break. The depth of daytime foraging suggests association of prey fields with the depth of the surface mixed layer, especially late in the migratory season where daytime diving was vertically concentrated 5–10 m below the MLD. Consistent with other studies, behavioral state was found to increase towards area-restricted search as the season progressed and as surface wind speed decreased. Individual females showed behavioral responses (movement alignment and changes to behavioral state) to some mesoscale features during the overwintering period, primarily in the CC ecosystem.

Observations from Seagliders support the idea that preferential habitat utilization in the near-shelf region reflects the abundant prey resources in the CC and basin-scale lateral gradients in MLD that may play a role in increasing accessibility of prey to adult females during the daytime. Three females equipped with dive recorders who traveled through the WA region demonstrated daytime diving to the base of the mixed layer and below, and in one case, movement behavior associated with the Columbia River plume, which is associated with a shallow MLD. Foraging within eddies may contribute to enhanced adult female NFS habitat utilization near eddy generation sites and contribute to the location of the peak in cross-shore distribution at the energetic outer edge of continental slope currents. Enhancements to prey spatial concentration and/or accessibility are possible reasons why females demonstrate behavioral responses to mesoscale eddies and other circulation features.

These results suggest that surface light levels and wind speed, oceanic MLD, coastal primary productivity, freshwater plumes, and eddy activity are important environmental factors that influence the distribution of adult female NFS and their behavioral responses within the destination ecosystems for overwinter foraging. Female prey composition and depth distribution during the overwintering period are areas that require further study, which may reveal more about the functional nature of the interaction between NFS diving and the MLD. The quantitative relationship between adult female behavior and mesoscale physical features during the overwintering period remains unclear and requires further observations in which subsurface structure can be studied along with behavioral responses or lack thereof. A more focused sampling effort using autonomous vehicles and satellite tagging of NFS could address this and other outstanding questions regarding the overwintering period in the future. Increased knowledge of how ocean conditions affect adult female behavior during overwinter migration is important to addressing questions surrounding demography during the pelagic phase of the NFS life cycle, and may shed light on how long-term climate patterns in the eastern Pacific affect the population as a whole.

## Supporting Information

Figure S1 **Generalized additive model residuals and response diagnostic plots.** (Top Left) Plot of residual quantile versus theoretical (normal) quantile. (Bottom Left) Histogram of residuals. Residuals are distributed in approximately normal fashion. (Top Right) Distribution of residuals versus a linear predictor. There is no evidence of significant change in the distribution of residuals as a function of the linear predictor value. (Bottom Right) Response variable versus modeled values.
(TIF)

Figure S2 **Monthly evolution of adult female northern fur seal cross-shore distribution.** January (A) through June (F). Blue bars in each plot are a histogram of estimated time spent in that month versus distance offshore from the shelf break in 20 km bins within the California Current and Gulf of Alaska Large Marine Ecosystems, north of 41°N and south of Haida Gwaii, during the winters 2002–03 to 2009–10. Above each plot, vertical red lines indicate the median cross-shore position in that month.
(TIF)

Figure S3 **Behavioral responses of adult female northern fur seal 626 to mesoscale circulation and surface wind speed.** (A) Overview of satellite-tracked locations of female 626 in the California Current and Gulf of Alaska ecosystems from 1 April 2008 onwards (track prior to this time was localized near the same positions as 1 April–1 May and is not shown in order to avoid visual clutter). Locations every 6 h are colored according to estimated behavioral state (scale at top, ARS = area-restricted search). Gray box in panel A indicates the spatial extent covered by Figure 14A, when seal 626 foraged in close proximity to the Seaglider region. Temporal extent covered by Figure 14A approximately overlaps with that of panel B in this figure. (B)–(G) Weekly intervals of seal 626's estimated locations and behavioral state in 6 h periods (filled color circles, scale at top) plotted over sea level anomaly (color contours, scale at top) and surface geostrophic velocity anomaly (Vel, black arrows, scale at top right). Each plot is centered on female seal 626's locations over the weekly period. Thick gray circles indicate the locations and approximate spatial extent of altimetry-identified mesoscale eddies from Chelton et al. [63]. Eddies are plotted as circular features though this is intended for illustration purposes only. After crossing 47°N on 6 May, 626 transited to the northwest, then transitioned to an area-restricted search near 49°N, 130°W. This location appeared to be at the boundary between a weak cyclonic (counterclockwise-rotating, locally low SLA values) eddy feature centered at 49.83°N, 129.25°W and a possibly-developing anticyclonic (clockwise, locally high SLA values) eddy at 48.75°N, 130.33°W. Seal 626 remained in this location until June, at which point she circuited the eddy in the same sense as its rotation, following the geostrophic surface current. (H) Plot of estimated behavioral state (red line, scale on right axis) and 10 m height wind speed at 626's location (blue, scale on left y-axis) versus time for the overwintering period 2007–08. Wind speed estimates are obtained by interpolating National Centers for Environmental Prediction Reanalysis 2 (NCEP2) product to 626's estimated locations at each 6 h time point. Gray bars in alternating shading display the extent of time covered by panels B–G. Behavioral state for 626 indicated area-restricted search almost uniformly from 10 May to 14 June. This was the longest period of prolonged area-restricted search state for 626's recorded migration. Like other prolonged search periods shown, 10 May–14 June was characterized by lower overall surface wind speeds than the remainder of the satellite record for this individual, though there were still instances of elevated (>10 m s^−1^) winds (e.g., temporal extent corresponding to panels D, F, and G). During these times seal 626's behavioral state was temporarily reduced from the maximum area-restricted search index, though did not fully transition to transient behavior, possibly owing to the short duration of these wind events.
(TIF)

Figure S4 **Behavioral responses of adult female northern fur seal 628 to mesoscale circulation and surface wind speed.** (A) Overview of satellite-tracked locations of female 628 in the California Current and Gulf of Alaska ecosystems from 24 December 2007 onwards. Six h locations are colored according to estimated behavioral state (scale at top, ARS = area-restricted search). Gray box in panel A and gray shading in panel H indicates the spatial and temporal extent covered by Figure 15A, when 628 foraged in close proximity to the Seaglider region. (B)–(G) Weekly intervals of seal 628's estimated locations and behavioral state in 6 h periods (filled color circles) plotted over sea level anomaly (color contours, scale at top) and surface geostrophic velocity anomaly (Vel, black arrows, scale at top right). Each plot is centered on female seal 628's locations over the weekly period. Thick gray circles indicate the locations and approximate spatial extent of altimetry-identified mesoscale eddies from Chelton et al. [63]. Eddies are plotted as circular features though this is intended for illustration purposes only. These panels illustrate transit of 628 through a cyclonic eddy at 37°N, 128.5°W from 26 January to 8 March 2008. Seal 628 moved through a highly elliptical anticyclonic feature, encountered the cyclone, and like female seal 626 moved through this feature with the same sense as the cyclonic currents in panels C–E. Seal 628 then encountered a second cyclone and its movement was briefly aligned with this feature as well in panel F. (H) Plot of estimated behavioral state (red line, scale on right y-axis) and 10 m height wind speed at 628's location (blue, scale on left y-axis) versus time for the overwintering period 2007–08. Wind estimates are obtained by interpolating National Centers for Environmental Prediction Reanalysis 2 (NCEP2) product to 628's estimated locations at each 6 h time point. Gray bars in alternating shading display the extent of time covered by panels B–G. Seal 628's estimated behavioral state in panels B–G was uniformly indicative of area-restricted search, despite occasional increases in surface wind speed. The wind speed and behavioral state history of 628 illustrates higher wind speeds encountered during the transit phase in comparison to those encountered in the California Current (February–June).
(TIF)

Figure S5
**Adult female northern fur seal habitat utilization relative to eddy features, conditioned by estimated behavioral state (evaluated for tracks >200 d only).** (A) One-dimensional radial probability density functions [PDFs, 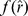] and cumulative distribution functions [CDFs, 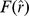] for habitat utilization relative to eddy features at two categories of behavioral state 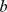 (transit = dark gray, area-restricted search [ARS] = light gray). “Transit” periods are those with 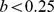; “ARS” (area-restricted search) periods are those with 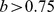. Solid lines show PDFs, dashed lines are CDFs (scale on right y-axis). Distributions are computed as a function of normalized distance to the nearest eddy center 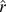, defined as absolute distance to the nearest eddy divided by that eddy's radial length scale 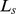 [63]. (B) Observed difference between search PDF and transit PDF (solid black line) with 95% confidence bounds computed using a bootstrap method (gray shading). Probability density functions show significant differences near 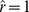 (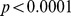) and 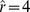 (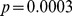). (C) Bootstrap distribution of 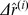, the difference of median values of 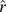 between ARS and transit states on bootstrap iteration 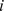. Solid black line shows the PDF of these values, thick dashed line shows CDF (scale at right). Thin vertical dashed line is the observed value of 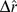. The gray shaded area denotes the bias-corrected/accelerated 95% confidence interval on the observed value of 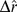. For the observed value of 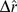 to be significantly different from zero using a two-tailed test, this confidence interval must not include zero.(TIF)

Supporting Methodology S1 **Processing of fluorescence measurements from Seagliders.**
(DOCX)

## References

[1] Ream RR, Sterling JT, Loughlin TR (2005) Oceanographic influences on northern fur seal migratory movements. Deep Sea Res Part II 52: 823–843.

[2] Trites AW (1991) Fetal growth of northern fur seals: life-history strategy and sources of variation. Can J Zool 69: 2608–2617.

[3] Trites AW (1992) Northern fur seals: why have they declined. Aquat Mamm 18: 3–18.

[4] Bigg MA (1982) Migration of northern fur seals in the eastern North Pacific and eastern Bering Sea: an analysis using effort and population composition data. Submitted to the standing scientific committee, 25th annual meeting of the North Pacific Fur Seal Commission. 77 p. Available: NMML Library, Alaska Fisheries Science Center, 7600 Sand Point Way NE, Seattle WA 98115.

[5] Gentry RL, Costa DP, Croxall JP, David JHM, Davis RW, et al.. (1986) Synthesis and conclusions. In: Gentry RL, Kooyman GL, editors. Fur seals: maternal strategies on land and at sea. Princeton NJ: Princeton University Press. pp. 220–264.

[6] Polovina JJ, Howell EA, Kobayashi DR, Seki MP (2001) The transition zone chlorophyll front, a dynamic global feature defining migration and forage habitat for marine resources. Prog Oceanogr 49: 469–483.

[7] Chapman DG (1961) A critical study of Pribilof fur seal population estimates. Fish Bull 63: 657–669.

[8] Eberhardt L (1981) Population dynamics of the Pribilof fur seals. In: Fowler CW, Smith TD, editors. Dynamics of large mammal populations. New York NY: John Wiley & Sons, Inc. pp. 197–220.

[9] Lander RH (1979) Role of land and ocean mortality in yield of male Alaskan fur seal, *Callorhinus ursinus* . Fish Bull 77: 311–314.

[10] Lander RH (1981) A life table and biomass estimate for Alaskan fur seals. Fish Res 1: 55–70.

[11] York AE, Hartley JR (1981) Pup production following harvest of female northern fur seals. Can J Fish Aquat Sci 38: 84–90.

[12] French DP, Reed M (1990) Potential impact of entanglement in marine debris on the population dynamics of the northern fur seal, *Callorhinus ursinus* In: Shomura RS, Godfrey ML, editors. Proceedings of the second international conference on marine debris, 2–7 April 1989. Honolulu HI: US Department of Commerce. NOAA Technical Memorandum NMFS-SWFSC-154. pp. 431–452. Available: http://http://swfsc.noaa.gov/publications/TM/SWFSC/NOAA-TM-NMFS-SWFSC-154_TOC.PDF.

[13] Springer A, Estes J, van Vliet G, Williams T, Doak D, et al (2003) Sequential megafaunal collapse in the North Pacific Ocean: an ongoing legacy of industrial whaling? Proc Nat Acad Sci USA 100: 12223–12228. 14526101 10.1073/pnas.1635156100 PMC218740

[14] York AE (1995) The relationship of several environmental indices to the survival of juvenile male northern fur seals (*Callorhinus ursinus*) from the Pribilof Islands. Can Spec Publ Fish Aquat Sci 121: 317–327.

[15] NMFS (2007) Conservation plan for the Eastern Pacific Stock of northern fur seal (*Callorhinus ursinus*). Washington DC: Department of Commerce/NOAA/Protected Resources Division, Alaska Region. 136 p. Available: http://www.fakr.noaa.gov/protectedresources/seals/fur/cplan/final1207.pdf.

[16] Towell RG, Ream RR, Bengtson JL, Sterling JT (2011) 2010 northern fur seal pup production and adult male counts on the Pribilof Islands, Alaska. Seattle WA: Alaska Fisheries Science Center. 6 p. Available: http://www.afsc.noaa.gov/nmml/pdf/2010-nfs-pup-estimates.pdf.

[17] Melin SR, Sterling JT, Ream RR, Towell RG, Zeppelin TK, et al. (2012) A tale of two stocks: studies of northern fur seals breeding at the northern and southern extent of the range. Seattle WA: NOAA Fisheries Service. Alaska Fisheries Science Center quarterly report feature (April-May-June 2012). 11 p. Available: http://www.afsc.noaa.gov/Quarterly/amj2012/amj12featurelead.htm.

[18] Ream RR, Baker JD, Towell RG (1999) Bogoslof Island studies. In: Sinclair EH, Robson BW, editors. Fur seal investigations, 1997. Seattle WA: National Marine Fisheries Service. NOAA Technical Memorandum NMFS-AFSC-106. pp. 81–92. Available: http://www.afsc.noaa.gov/Publications/AFSC-TM/NOAA-TM-AFSC-106.pdf.

[19] Towell RG, Ream RR, York AE (2006) Decline in northern fur seal (*Callorhinus ursinus*) pup production on the Pribilof Islands. Mar Mamm Sci 22: 486–491.

[20] Kuhn CE, Baker JD, Towell RG, Ream RR (2014) Evidence of localized resource depletion following a natural colonization event by a large marine predator. J Anim Ecol doi: 10.1111/1365-2656.12202 10.1111/1365-2656.12202 24450364

[21] Philander SGH (1983) El Niño southern oscillation phenomena. Nature 302: 295–301.

[22] DeLong R, Antonelis G (1991) Impact of the 1982–1983 El Niño on the northern fur seal population at San Miguel Island, California. In: Trillmich F, Ono KA, editors. Pinnipeds and El Niño: responses to environmental stress. Berlin, Germany: Springer-Verlag. pp. 75–83.

[23] Kenyon KW, Wilke F (1953) Migration of the northern fur seal, *Callorhinus ursinus* . J Mammal 34: 86–98.

[24] Loughlin TR, Baba N, Robson BW (1999) Use of surface-current model and satellite telemetry to assess marine mammal movements in the Bering Sea. In: Loughlin TR, Ohtani K, editors. Dynamics of the Bering Sea. Fairbanks AK: University of Alaska Sea Grant. pp. 615–630.

[25] Sterling JT, Springer AM, Iverson SJ, Johnson SP, Pelland NA, et al (2014) The sun, moon, wind, and biological imperative—shaping contrasting wintertime migration and foraging strategies of adult male and female northern fur seals (*Callorhinus ursinus*). PLoS ONE 9: e93068. 24722344 10.1371/journal.pone.0093068 PMC3983057

[26] Boyd IL, McCafferty DJ, Reid K, Taylor R, Walker TR (1998) Dispersal of male and female Antarctic fur seals (*Arctocephalus gazella*). Can J Fish Aquat Sci 55: 854–852.

[27] Dalton AJM, Rosen DAS, Trites AW (2014) Broad thermal capacity facilitates the primarily pelagic existence of northern fur seals (*Callorhinus ursinus*). Mar Mamm Sci 30: 994–1013.

[28] Rosen DAS, Trites AW (2014) Thermal limits in young northern fur seals, *Callorhinus ursinus* . Mar Mamm Sci 30: 1014–1018.

[29] Gentry RL (1998) Behavior and ecology of the northern fur seal. Princeton NJ: Princeton University Press. 392 p.

[30] Checkley DM Jr, Barth JA (2009) Patterns and processes in the California current system. Prog Oceanogr 83: 49–64.

[31] Kajimura H (1982) Opportunistic feeding of the northern fur seal *Callorhinus ursinus*, in the eastern North Pacific Ocean and eastern Bering Sea [PhD Thesis]. Tokyo, Japan: University of Tokyo. 150 p.

[32] Zeppelin TK, Orr AJ (2010) Stable isotope and scat analyses indicate diet and habitat partitioning in northern fur seals *Callorhinus ursinus* across the eastern Pacific. Mar Ecol Prog Ser 409: 241–253.

[33] Trillmich F, Ono KA (1991) Pinnipeds and El Niño: responses to environmental stress. Berlin, Germany: Springer-Verlag. 293 p.

[34] Weise MJ, Costa DP, Kudela RM (2006) Movement and diving behavior of male California sea lion (*Zalophus californianus*) during anomalous oceanographic conditions of 2005 compared to those of 2004. Geophys Res Lett 33: L22S10.

[35] Trillmich F (1986) Attendance behavior of Galapagos fur seals. In: Gentry RL, Kooyman GL, editors. Fur seals: maternal strategies on land and at sea. Princeton NJ: Princeton University Press. pp. 168–185.

[36] Trillmich F, Dellinger T (1991) The effects of El Niño on Galapagos pinnipeds. In: Trillmich F, Ono KA, editors. Pinnipeds and El Niño: responses to environmental stress. Berlin, Germany: Springer-Verlag. pp. 66–74.

[37] Baker JD, Howell EA, Polovina JJ (2012) Relative influence of climate variability and direct anthropogenic impact on a sub-tropical Pacific top predator, the Hawaiian monk seal. Mar Ecol Prog Ser 469: 175–189.

[38] Mantua N, Hare S, Zhang Y, Wallace J, Francis R (1997) A Pacific interdecadal climate oscillation with impacts on salmon production. Bull Am Meteorol Soc 78: 1069–1079.

[39] Sterling JT (2009) Northern fur seal foraging behaviors, food webs, and interactions with oceanographic features in the eastern Bering Sea [Ph.D. Thesis]. Seattle WA: University of Washington. 219 p.

[40] Nordstrom CA, Battaile BC, Cotté C, Trites AW (2013) Foraging habitats of lactating northern fur seals are structured by thermocline depths and submesoscale fronts in the eastern Bering Sea. Deep Sea Res Part II 88–89: 78–96.

[41] Bailleul F, Cotté C, Guinet C (2010) Mesoscale eddies as foraging area of a deep-diving predator, the southern elephant seal. Mar Ecol Prog Ser 408: 251–264.

[42] Campagna C, Piola AR, Rosa Marin M, Lewis M, Fernandez T (2006) Southern elephant seal trajectories, fronts and eddies in the Brazil/Malvinas confluence. Deep Sea Res Part I 53: 1907–1924.

[43] Nel DC, Lutjeharms JRE, Pakhomov EA, Ansorge IJ, Ryan PG, et al (2001) Exploitation of mesoscale oceanographic features by grey-headed albatross *Thalassarche chrysostoma* in the southern Indian Ocean. Mar Ecol Prog Ser 217: 15–26.

[44] Yen PPW, Sydeman WJ, Bograd SJ, Hyrenbach KD (2006) Spring-time distributions of migratory marine birds in the southern California current: oceanic eddy associations and coastal habitat hotspots over 17 years. Deep Sea Res Part II 53: 399–418.

[45] Yoda K, Shiomi K, Sato K (2014) Foraging spots of streaked shearwaters in relation to ocean surface currents as identified using their drift movements. Prog Oceanogr 122: 54–64.

[46] Haney JC, McGillivary PA (1985) Aggregations of Cory's shearwaters (*Calonectris diomedea*) at Gulf Stream fronts. Wilson Bull 97: 191–200.

[47] Baker JD (2007) Post-weaning migration of northern fur seal *Callorhinus ursinus* pups from the Pribilof Islands, Alaska. Mar Ecol Prog Ser 341: 243–255.

[48] Hooper CL (1895) Report of Captain Hooper, dated November 21, 1892. In: US Senate. 53rd Congress, 2nd session. Fur-seal arbitration, proceedings of the Tribunal of Arbitration, volume VII, Senate executive document 177 (serial set 3166). Washington DC: Government Printing Office. pp. 228–233.

[49] Lea MA, Johnson D, Ream R, Sterling J, Melin S, et al (2009) Extreme weather events influence dispersal of naive northern fur seals. Biol Letters 5: 252–257. 10.1098/rsbl.2008.0643 PMC2665815 19147444

[50] Peterson RS (1965) The behavior of the northern fur seal [Ph.D. Thesis]. Baltimore MD: John Hopkins University. 214 p.

[51] Eriksen CC, Osse TJ, Light RD, Wen T, Lehman TW, et al (2001) Seaglider: a long-range autonomous underwater vehicle for oceanographic research. IEEE J Oceanic Eng 26: 424–436.

[52] US Senate. 54th Congress, 1st session. (1896) Seal life on rookeries of the Pribilof Islands, 1893–95, part II, Senate document 137 (serial set 3351). Washington DC: Government Printing Office. 154 p.

[53] Sherman KR (1991) The large marine ecosystem concept: research and management strategy for living marine resources. Ecol Appl 350–360. 10.2307/1941896 27755673

[54] Loughlin TR, Sterling JT, Merrick RL, Sease JL, York AE (2003) Diving behavior of immature Steller sea lions (*Eumetopias jubatus*). Fish Bull 101: 566–582.

[55] Lea MA, Johnson D, Melin S, Ream RR, Gelatt T (2010) Diving ontogeny and lunar responses in a highly migratory mammal, the northern fur seal *Callorhinus ursinus* . Mar Ecol Prog Ser 419: 233–247.

[56] Block BA, Jonsen ID, Jorgensen SJ, Winship AJ, Shaffer SA, et al (2011) Tracking apex marine predator movements in a dynamic ocean. Nature 475: 86–90. 21697831 10.1038/nature10082

[57] Breed GA, Jonsen ID, Myers RA, Bowen WD, Leonard ML (2009) Sex-specific, seasonal foraging tactics of adult grey seals (*Halichoerus grypus*) revealed by state-space analysis. Ecology 90: 3209–3221. 19967876 10.1890/07-1483.1

[58] Jonsen I, Myers R, Flemming J (2003) Meta-analysis of animal movement using state-space models. Ecology 84: 3055–3063.

[59] Jonsen ID, Flemming JM, Myers RA (2005) Robust state-space modeling of animal movement data. Ecology 86: 2874–2880.

[60] Jonsen ID, Myers RA, James MC (2007) Identifying leatherback turtle foraging behaviour from satellite telemetry using a switching state-space model. Mar Ecol Prog Ser 337: 255–264.

[61] Jonsen ID, Basson M, Bestley S, Bravington MV, Patterson TA, et al (2013) State-space models for bio-loggers: a methodological road map. Deep Sea Res Part II 88–89: 34–46.

[62] Worton BJ (1995) Using Monte Carlo simulation to evaluate kernel-based home range estimators. J Wildl Manag 59: 794–800.

[63] Chelton DB, Schlax MG, Samelson RM (2011) Global observations of nonlinear mesoscale eddies. Prog Oceanogr 91: 167–216.

[64] DiCiccio TJ, Efron B (1996) Bootstrap confidence intervals. Stat Sci 11: 189–212.

[65] Venables WN, Dichmont CM (2004) GLMs, GAMs and GLMMs: an overview of theory for applications in fisheries research. Fish Res 70: 319–337.

[66] Benson SR, Eguchi T, Foley DG, Forney KA, Bailey H, et al (2011) Large-scale movements and high-use areas of western Pacific leatherback turtles, *Dermochelys coriacea* . Ecosphere 2: art84.

[67] Akaike H (1998) Information theory and an extension of the maximum likelihood principle. In: Parzen E, Tanabe K, Kitagawa G, editors. Selected papers of Hirotugu Akaike. New York NY: Springer. pp. 199–213.

[68] Perry M, Sackmann B, Eriksen C, Lee C (2008) Seaglider observations of blooms and subsurface chlorophyll maxima off the Washington coast. Limnol Oceanogr 53: 2169–2179.

[69] Sackmann B (2007) Remote assessment of 4-D phytoplankton distributions off the Washington coast [Ph.D. Thesis]. Orono ME: University of Maine. 204 p.

[70] Pelland NA, Eriksen CC, Lee CM (2013) Subthermocline eddies over the Washington continental slope as observed by Seagliders, 2003–09. J Phys Oceanogr 43: 2025–2053.

[71] de Boyer Montégut C, Madec G, Fischer AS, Lazar A, Iudicone D (2004) Mixed layer depth over the global ocean: an examination of profile data and a profile-based climatology. J Geophys Res-Oceans 109: C12003.

[72] Sackmann BS, Perry MJ, Eriksen CC (2008) Seaglider observations of variability in daytime fluorescence quenching of chlorophyll-*a* in northeastern Pacific coastal waters. Biogeosciences Discuss 5: 2839–2865.

[73] Carroll M, Chigounis D, Gilbert S, Gundersen K, Hayashi K, et al.. (2006) Performance verification statement for the WET Labs ECO FLNTUSB fluorometer. Solomons MD: Alliance for Coastal Technologies. 36 p.

[74] Chaigneau A, Eldin G, Dewitte B (2009) Eddy activity in the four major upwelling systems from satellite altimetry (1992–2007). Prog Oceanogr 83: 117–123.

[75] Feldman GC, McClain CR (2013) Ocean color web, SeaWiFS/MODIS Aqua reprocessing level-2. Kuring N, Bailey SW editors. Greenbelt MD: NASA Goddard Space Flight Center. Accessed: 1 January 2013.

[76] Freeland HJ, Cummins PF (2005) Argo: a new tool for environmental monitoring and assessment of the world's oceans, an example from the NE Pacific. Prog Oceanogr 64: 31–44.

[77] Li M, Myers PG, Freeland H (2005) An examination of historical mixed layer depths along Line P in the Gulf of Alaska. Geophys Res Lett 32: L05613.

[78] Hickey BM (1989) Patterns and processes of circulation over the Washington continental shelf and slope. In: Landry MR, Hickey BM, editors. Coastal oceanography of Washington and Oregon. Elsevier oceanography series, volume 47. Amsterdam, Netherlands: Elsevier Science. pp. 41–115.

[79] Fong DA, Geyer WR (2001) Response of a river plume during an upwelling favorable wind event. J Geophys Res-Oceans 106: 1067–1084.

[80] Hickey BM (1998) Coastal oceanography of western North America from the tip of Baja to Vancouver Island. In: Robinson A, Brink S, editors. The global coastal ocean: regional studies and syntheses. The Sea, volume 11. New York NY: Wiley. pp. 345–393.

[81] Hickey BM, Kudela RM, Nash JD, Bruland KW, Peterson WT, et al (2010) River influences on shelf ecosystems: introduction and synthesis. J Geophys Res-Oceans 115: C00B17.

[82] García Berdeal I, Hickey BM, Kawase M (2002) Influence of wind stress and ambient flow on a high discharge river plume. J Geophys Res-Oceans 107: 13-1–13-24.

[83] Bigg MA (1986) Arrival of northern fur seals, *Callorhinus ursinus*, on St. Paul Island, Alaska. Fish Bull 84: 383–394.

[84] Olesiuk PF (2012) Habitat utilization by northern fur seals (*Callorhinus ursinus*) in the northeastern Pacific Ocean and Canada. Ottawa, Canada: Department of Fisheries and Oceans. Canadian Science Advisory Secretariat Research Document 2012/040. 27 p. Available: http://publications.gc.ca/site/eng/436921/publication.html.

[85] Kajimura H, Lander RH, Perez MA, York AE, Bigg MA (1980) Further analysis of pelagic fur seal data collected by the United States and Canada during 1958–74, part I. Submitted to the standing scientific committee, 23rd annual meeting of the North Pacific fur seal commission. 94 p. Available: NMML Library, Alaska Fisheries Science Center, 7600 Sand Point Way NE, Seattle WA 98115.

[86] Kosro PM, Huyer A, Ramp SR, Smith RL, Chavez FP, et al (1991) The structure of the transition zone between coastal waters and the open ocean off northern California, winter and spring 1987. J Geophys Res-Oceans 96: 14707–14730.

[87] Bigg MA (1990) Migration of northern fur seals (*Callorhinus ursinus*) off western North America. Can Tech Rep Fish Aquat Sci 1764

[88] Gentry RL, Kooyman GL, Goebel ME (1986) Feeding and diving behavior of northern fur seals. In: Gentry RL, Kooyman GL, editors. Fur seals: maternal strategies on land and at sea. Princeton NJ: Princeton University Press. pp. 61–78.

[89] Goebel ME, Bengtson JL, Delong RL, Gentry RL, Loughlin TR (1991) Diving patterns and foraging locations of female northern fur seals. Fish Bull 89: 171–179.

[90] Kajimura H (1984) Opportunistic feeding of the northern fur seal, *Callorhinus ursinus*, in the eastern North Pacific Ocean and eastern Bering Sea. Seattle WA: National Marine Fisheries Service. NOAA Technical Report NMFS SSRF-779. 49 p. Available: http://spo.nmfs.noaa.gov/SSRF/SSRF779.pdf.

[91] Sinclair E, Loughlin T, Pearcy W (1994) Prey selection by northern fur seals (*Callorhinus ursinus*) in the eastern Bering Sea. Fish Bull 92: 144–156.

[92] Sterling JT, Ream RR (2004) At-sea behavior of juvenile male northern fur seals. Can J Zool 82: 1621–1637.

[93] Suga T, Motoki K, Aoki Y, Macdonald AM (2004) The North Pacific climatology of winter mixed layer and mode waters. J Phys Oceanogr 34: 3–22.

[94] Staniland IJ, Robinson SL (2008) Segregation between the sexes: Antarctic fur seals, *Arctocephalus gazella*, foraging at South Georgia. Anim Behav 75: 1581–1590.

[95] Costa DP, Gentry RL (1986) Free-ranging energetics of northern fur seals. In: Gentry RL, Kooyman GL, editors. Fur seals: maternal strategies on land and at sea. Princeton NJ: Princeton University Press. pp. 79–101.

[96] Schusterman RJ, Balliet RF (1971) Aerial and underwater visual acuity in the California sea lion (*Zalophus californianus*) as a function of luminance. Ann NY Acad Sci 188: 37–46. 5288866 10.1111/j.1749-6632.1971.tb13088.x

[97] Alverson DL, Larkins H (1969) Status of knowledge of the Pacific hake resource. Calif Coop Oceanic Fish Invest Rep 13: 24–31.

[98] Appenzeller A, Leggett W (1992) Bias in hydroacoustic estimates of fish abundance due to acoustic shadowing: evidence from day-night surveys of vertically migrating fish. Can J Fish Aquat Sci 49: 2179–2189.

[99] Bertrand A, Gerlotto F, Bertrand S, Gutiérrez M, Alza L, et al (2008) Schooling behaviour and environmental forcing in relation to anchoveta distribution: an analysis across multiple spatial scales. Prog Oceanogr 79: 264–277.

[100] Austin D, Bowen WD, McMillan JI, Boness DJ (2006) Stomach temperature telemetry reveals temporal patterns of foraging success in a free-ranging marine mammal. J Anim Ecol 75: 408–420. 16637994 10.1111/j.1365-2656.2006.01057.x

[101] Kelly KA, Beardsley RC, Limeburner R, Brink KH, Paduan JD, et al (1998) Variability of the near-surface eddy kinetic energy in the California current based on altimetric, drifter, and moored current data. J Geophys Res-Oceans 103: 13067–13083.

[102] Benoit-Bird KJ, Battaile BC, Heppell SA, Hoover B, Irons D, et al (2013) Prey patch patterns predict habitat use by top marine predators with diverse foraging strategies. PLoS ONE 8: e53348. 23301063 10.1371/journal.pone.0053348 PMC3536749

[103] Godø OR, Samuelsen A, Macaulay GJ, Patel R, Hjøllo SS, et al (2012) Mesoscale eddies are oases for higher trophic marine life. PLoS ONE 7: e30161. 22272294 10.1371/journal.pone.0030161 PMC3260222

[104] Collins CA, Pennington JT, Castro CG, Rago TA, Chavez FP (2003) The California current system off Monterey, California: physical and biological coupling. Deep Sea Res Part II 50: 2389–2404.

[105] Boccaletti G, Ferrari R, Fox-Kemper B (2006) Mixed layer instabilities and restratification. J Phys Oceanogr 37: 2228–2250.

[106] Mahadevan A, Thomas LN, Tandon A (2008) Comment on “Eddy/wind interactions stimulate extraordinary mid-ocean plankton blooms”. Science 320: 448. 10.1126/science.1152111 18436758

[107] Niiler PP (1969) On the Ekman divergence in an oceanic jet. J Geophys Res 74: 7048–7052.

[108] Ainley DG, Dugger KD, Ford RG, Pierce SD, Reese DC, et al (2009) Association of predators and prey at frontal features in the California current: competition, facilitation, and co-occurrence. Mar Ecol Prog Ser 389: 271–294.

[109] Ainley DG, Spear LB, Tynan CT, Barth JA, Pierce SD, et al (2005) Physical and biological variables affecting seabird distributions during the upwelling season of the northern California current. Deep Sea Res Part II 52: 123–143.

[110] Logerwell EA, Smith PE (2001) Mesoscale eddies and survival of late stage Pacific sardine (*Sardinops sagax*) larvae. Fish Oceanogr 10: 13–25.

[111] Nishimoto MM, Washburn L (2002) Patterns of coastal eddy circulation and abundance of pelagic juvenile fish in the Santa Barbara Channel, California, USA. Mar Ecol Prog Ser 241: 183–199.

[112] Lara-Lopez AL, Davison P, Koslow JA (2012) Abundance and community composition of micronekton across a front off southern California. J Plankton Res 34: 828–848.

[113] Henson SA, Thomas AC (2008) A census of oceanic anticyclonic eddies in the Gulf of Alaska. Deep Sea Res Part I 55: 163–176.

[114] Ladd C, Crawford WR, Harpold CE, Johnson WK, Kachel NB, et al (2009) A synoptic survey of young mesoscale eddies in the eastern Gulf of Alaska. Deep Sea Res Part II 56: 2460–2473.

[115] Ladd C, Kachel NB, Mordy CW, Stabeno PJ (2005) Observations from a Yakutat eddy in the northern Gulf of Alaska. J Geophys Res-Oceans 110: C03003.

[116] Ladd C, Mordy CW, Kachel NB, Stabeno PJ (2007) Northern Gulf of Alaska eddies and associated anomalies. Deep Sea Res Part I 54: 487–509.

[117] Okkonen SR, Weingartner TJ, Danielson SL, Musgrave DL, Schmidt GM (2003) Satellite and hydrographic observations of eddy-induced shelf-slope exchange in the northwestern Gulf of Alaska. J Geophys Res-Oceans 108: 3033.

[118] Whitney FA, Crawford WR, Harrison PJ (2005) Physical processes that enhance nutrient transport and primary productivity in the coastal and open ocean of the subarctic NE Pacific. Deep Sea Res Part II 52: 681–706.

[119] Crawford WR, Brickley PJ, Peterson TD, Thomas AC (2005) Impact of Haida eddies on chlorophyll distribution in the eastern Gulf of Alaska. Deep Sea Res Part II 52: 975–989.

[120] Marchesiello P, McWilliams JC, Shchepetkin A (2003) Equilibrium structure and dynamics of the California current system. J Phys Oceanogr 33: 753–783.

[121] Paduan JD, Niiler PP (1993) Structure of velocity and temperature in the northeast Pacific as measured with Lagrangian drifters in fall 1987. J Phys Oceanogr 23: 585–600.

[122] Mackas DL, Tsurumi M, Galbraith MD, Yelland DR (2005) Zooplankton distribution and dynamics in a North Pacific eddy of coastal origin: II. Mechanisms of eddy colonization by and retention of offshore species. Deep Sea Res Part II 52: 1011–1035.

[123] Barth JA (1994) Short-wave length instabilities on coastal jets and fronts. J Geophys Res-Oceans 99: 16095–16115.

[124] Barth JA, Pierce SD, Cowles TJ (2005) Mesoscale structure and its seasonal evolution in the northern California current system. Deep Sea Res Part II 52: 5–28.

[125] Barth JA, Pierce SD, Smith RL (2000) A separating coastal upwelling jet at Cape Blanco, Oregon and its connection to the California current system. Deep Sea Res Part II 47: 783–810.

[126] Hayward TL, Mantyla AW (1990) Physical, chemical and biological structure of a coastal eddy near Cape Mendocino. J Mar Res 48: 825–850.

[127] Strub PT, James C (2000) Altimeter-derived variability of surface velocities in the California current system: 2. Seasonal circulation and eddy statistics. Deep Sea Res Part II 47: 831–870.

[128] Kurian J, Colas F, Capet X, McWilliams JC, Chelton DB (2011) Eddy properties in the California current system. J Geophys Res-Oceans 116.

[129] Ladd C (2007) Interannual variability of the Gulf of Alaska eddy field. Geophys Res Lett 34: L11605.

[130] Crawford W (2002) Physical characteristics of Haida eddies. J Oceanogr 58: 703–713.

[131] Miller BS, Simenstad CA, Cross JN, Fresh KL, Steinfort SN (1980) Nearshore fish and macroinvertebrate assemblages along the Strait of Juan de Fuca including food habits of the common nearshore fish. Seattle WA: University of Washington. Fisheries Research Institute Report 8001. 213 p. Available: http://hdl.handle.net/1773/3939.

[132] MacFadyen A, Hickey BM, Cochlan WP (2008) Influences of the Juan de Fuca eddy on circulation, nutrients, and phytoplankton production in the northern California current system. J Geophys Res-Oceans 113: C08008.

[133] Banas NS, MacCready P, Hickey BM (2009) The Columbia River plume as cross-shelf exporter and along-coast barrier. Cont Shelf Res 29: 292–301.

[134] Trites AW, Bigg MA (1996) Physical growth of northern fur seals (*Callorhinus ursinus*): seasonal fluctuations and migratory influences. J Zool (Lond) 238: 459–482.

[135] Large WG, McWilliams JC, Niiler PP (1986) Upper ocean thermal response to strong autumnal forcing of the northeast Pacific. J Phys Oceanogr 16: 1524–1550.

[136] Dohan K, Davis RW (2010) Mixing in the transition layer during two storm events. J Phys Oceanogr 41: 42–66.

[137] Scheffer VB (1950) Winter injury to young fur seals on the northwest coast. Calif Fish Game 36: 378–379.

[138] Kenyon KW, Scheffer VB, Chapman DG (1954) A population study of the Alaska fur seal herd. Washington DC: US Fish and Wildlife Service. Special Scientific Report - Wildlife, No. 12. 77 p.

[139] Ichihara T (1974) Possible effect of surface wind force on the sex-specific mortality of young fur seals in the eastern Pacific. Bull Far Seas Fish Res Lab 11: 1–8.

[140] Niggol K, Fiscus CH Jr, Wilke F (1959) Pelagic fur seal investigations California-Oregon-Washington, 1959. Seattle WA: US Fish and Wildlife Service. 92 p. Available: http://www.afsc.noaa.gov/nmml/library/nfsi/Pelagic-Fur-Seal-Investigations-1959.pdf.

