# Supplementary figures and images for: Fortuitous Encounters between Seagliders and Adult Female Northern Fur Seals (Callorhinus ursinus) off the Washington (USA) Coast: Upper Ocean Variability and Links to Top Predator Behavior
Source: PLoS One. 2014 Aug 25;9(8):e101268. doi: 10.1371/journal.pone.0101268 (PMC4143212; doi:10.1371/journal.pone.0101268)

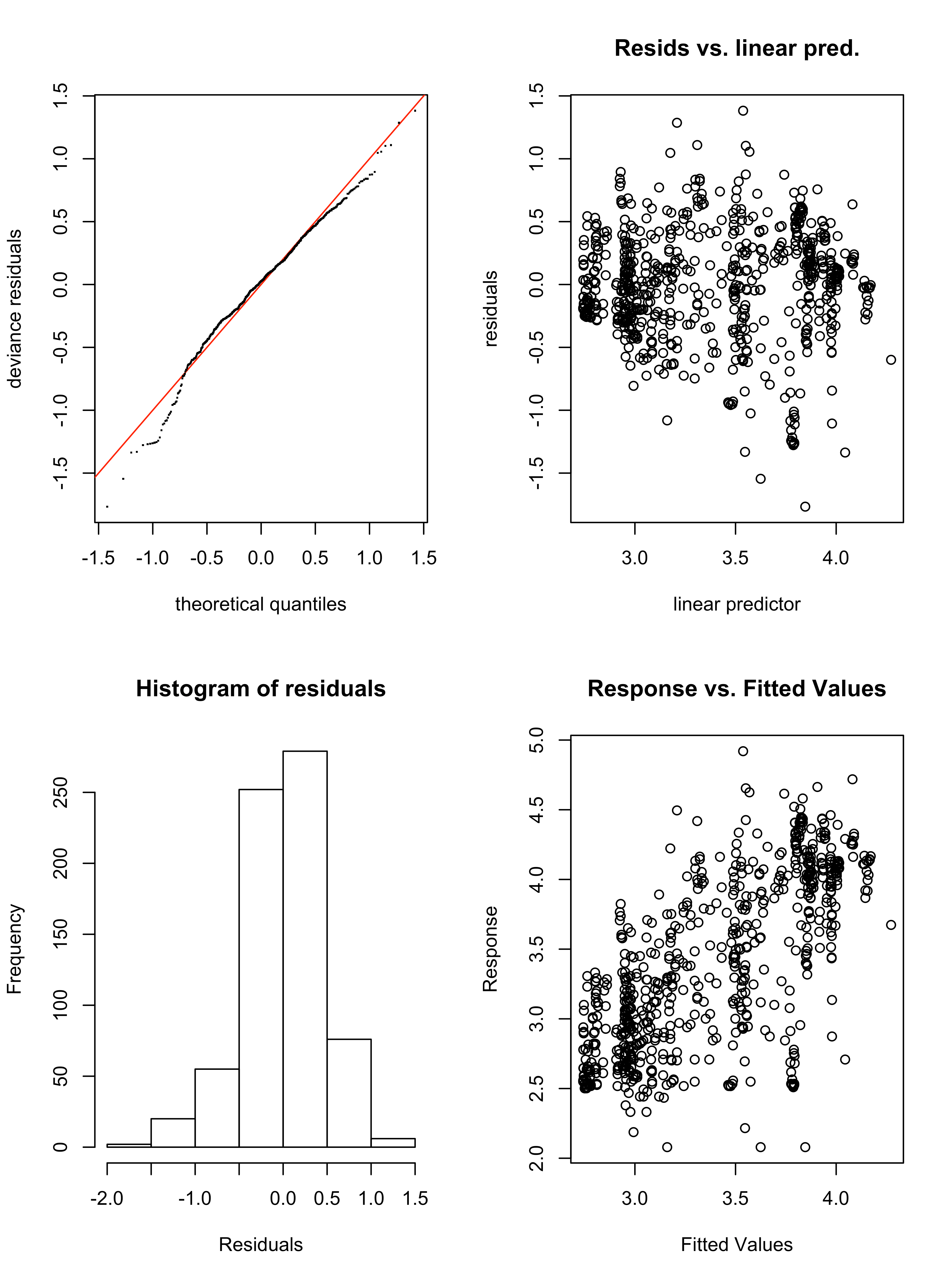

Supplement: Figure S1 — Generalized additive model residuals and response diagnostic plots. (Top Left) Plot of residual quantile versus theoretical (normal) quantile. (Bottom Left) Histogram of residuals. Residuals are distributed in approximately normal fashion. (Top Right) Distribution of residuals versus a linear predictor. There is no evidence of significant change in the distribution of residuals as a function of the linear predictor value. (Bottom Right) Response variable versus modeled values. (TIF) [file pone.0101268.s001.tif]

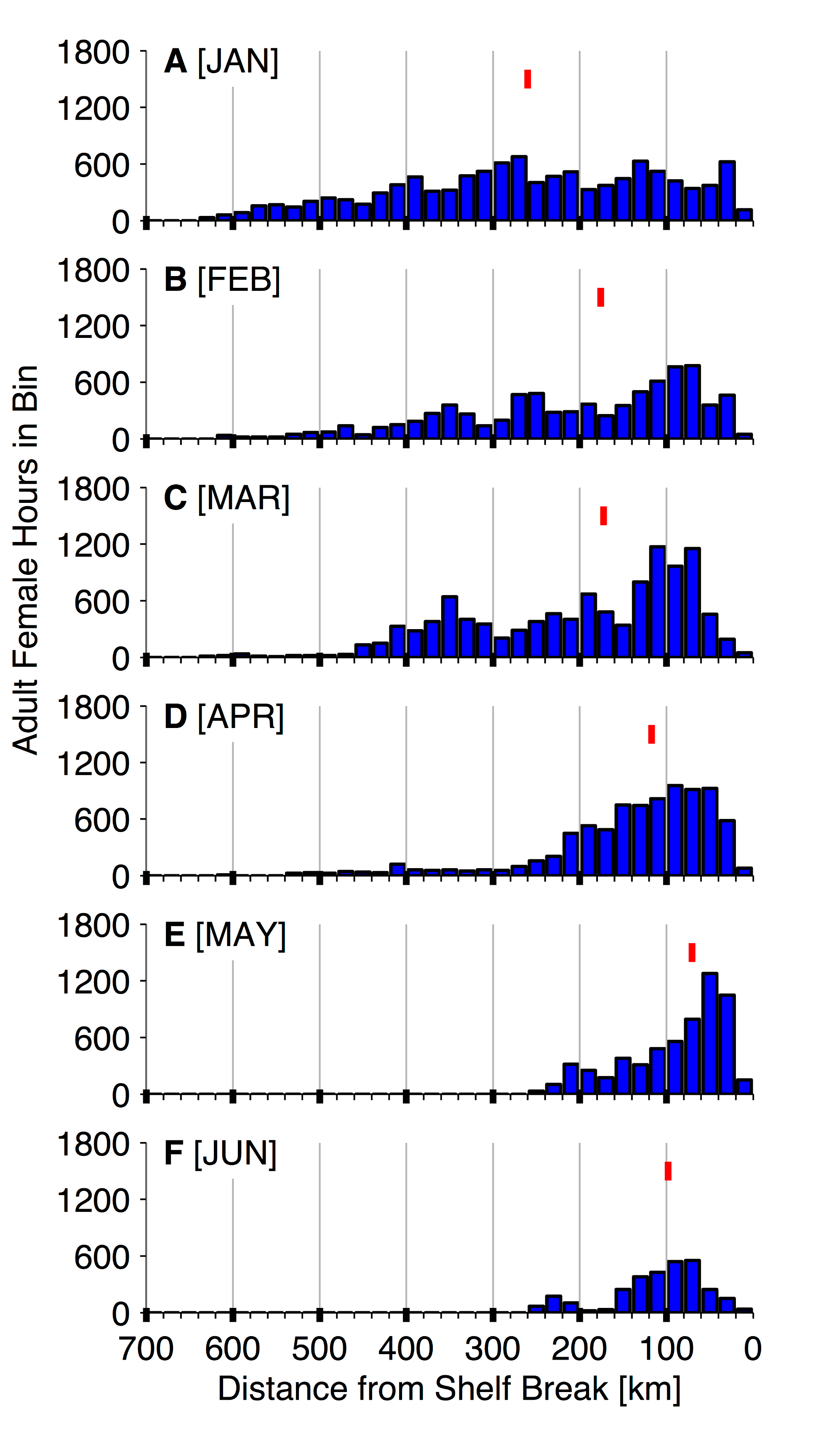

Supplement: Figure S2 — Monthly evolution of adult female northern fur seal cross-shore distribution. January (A) through June (F). Blue bars in each plot are a histogram of estimated time spent in that month versus distance offshore from the shelf break in 20 km bins within the California Current and Gulf of Alaska Large Marine Ecosystems, north of 41°N and south of Haida Gwaii, during the winters 2002–03 to 2009–10. Above each plot, vertical red lines indicate the median cross-shore position in that month. (TIF) [file pone.0101268.s002.tif]

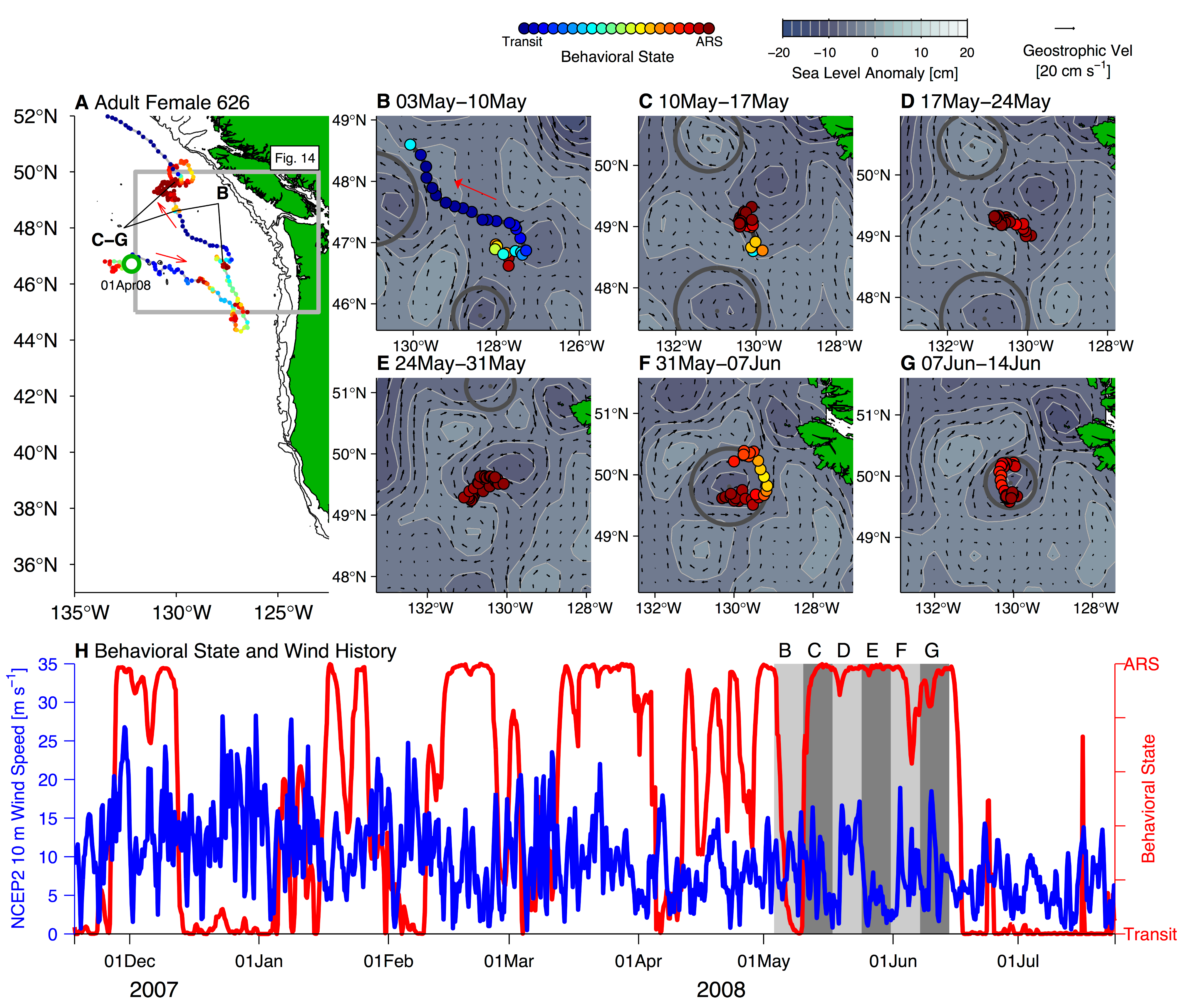

Supplement: Figure S3 — Behavioral responses of adult female northern fur seal 626 to mesoscale circulation and surface wind speed. (A) Overview of satellite-tracked locations of female 626 in the California Current and Gulf of Alaska ecosystems from 1 April 2008 onwards (track prior to this time was localized near the same positions as 1 April–1 May and is not shown in order to avoid visual clutter). Locations every 6 h are colored according to estimated behavioral state (scale at top, ARS = area-restricted search). Gray box in panel A indicates the spatial extent covered by Figure 14A, when seal 626 foraged in close proximity to the Seaglider region. Temporal extent covered by Figure 14A approximately overlaps with that of panel B in this figure. (B)–(G) Weekly intervals of seal 626's estimated locations and behavioral state in 6 h periods (filled color circles, scale at top) plotted over sea level anomaly (color contours, scale at top) and surface geostrophic velocity anomaly (Vel, black arrows, scale at top right). Each plot is centered on female seal 626's locations over the weekly period. Thick gray circles indicate the locations and approximate spatial extent of altimetry-identified mesoscale eddies from Chelton et al. [63]. Eddies are plotted as circular features though this is intended for illustration purposes only. After crossing 47°N on 6 May, 626 transited to the northwest, then transitioned to an area-restricted search near 49°N, 130°W. This location appeared to be at the boundary between a weak cyclonic (counterclockwise-rotating, locally low SLA values) eddy feature centered at 49.83°N, 129.25°W and a possibly-developing anticyclonic (clockwise, locally high SLA values) eddy at 48.75°N, 130.33°W. Seal 626 remained in this location until June, at which point she circuited the eddy in the same sense as its rotation, following the geostrophic surface current. (H) Plot of estimated behavioral state (red line, scale on right axis) and 10 m height wind speed at 626's location (bl [file pone.0101268.s003.tif]

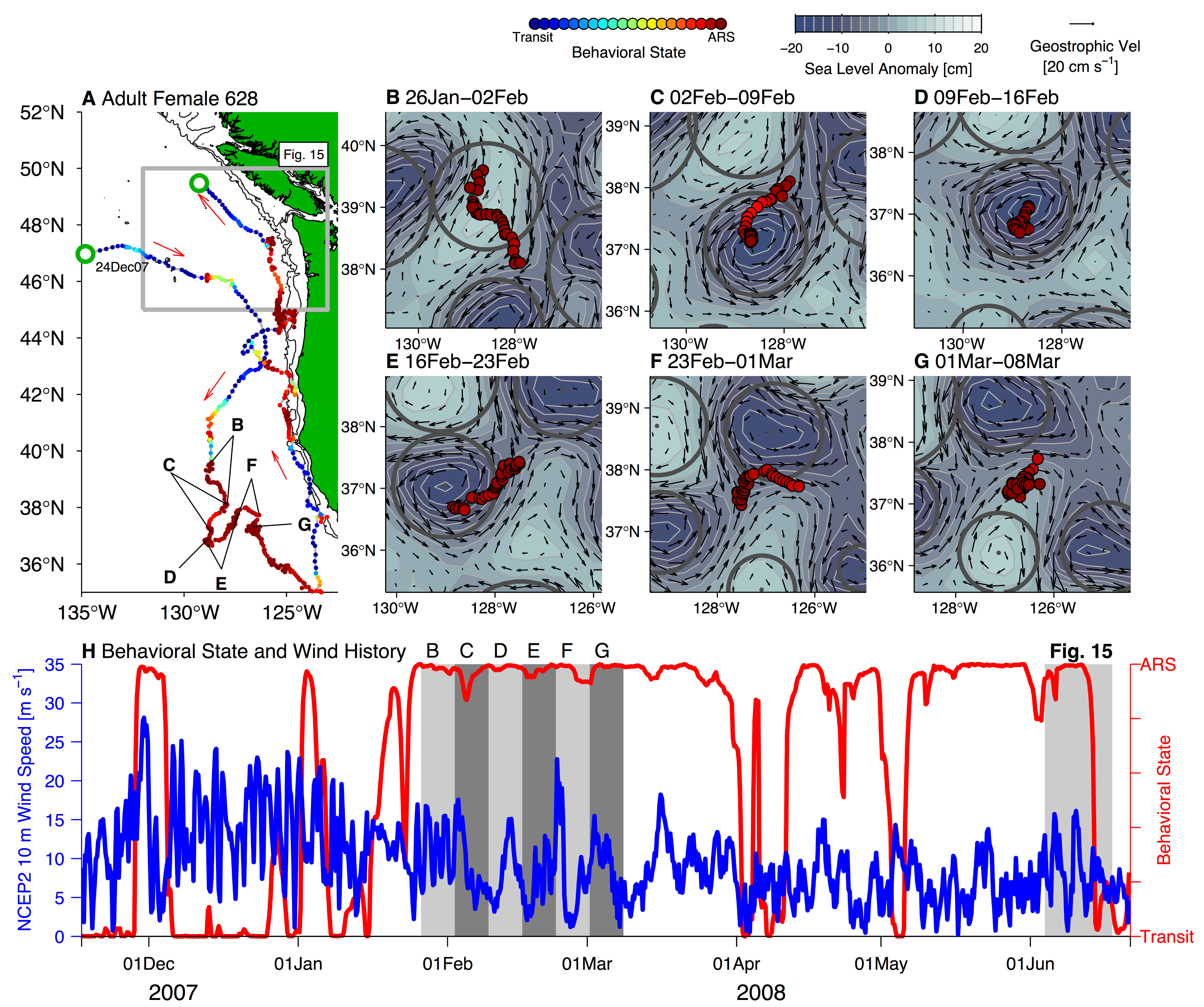

Supplement: Figure S4 — Behavioral responses of adult female northern fur seal 628 to mesoscale circulation and surface wind speed. (A) Overview of satellite-tracked locations of female 628 in the California Current and Gulf of Alaska ecosystems from 24 December 2007 onwards. Six h locations are colored according to estimated behavioral state (scale at top, ARS = area-restricted search). Gray box in panel A and gray shading in panel H indicates the spatial and temporal extent covered by Figure 15A, when 628 foraged in close proximity to the Seaglider region. (B)–(G) Weekly intervals of seal 628's estimated locations and behavioral state in 6 h periods (filled color circles) plotted over sea level anomaly (color contours, scale at top) and surface geostrophic velocity anomaly (Vel, black arrows, scale at top right). Each plot is centered on female seal 628's locations over the weekly period. Thick gray circles indicate the locations and approximate spatial extent of altimetry-identified mesoscale eddies from Chelton et al. [63]. Eddies are plotted as circular features though this is intended for illustration purposes only. These panels illustrate transit of 628 through a cyclonic eddy at 37°N, 128.5°W from 26 January to 8 March 2008. Seal 628 moved through a highly elliptical anticyclonic feature, encountered the cyclone, and like female seal 626 moved through this feature with the same sense as the cyclonic currents in panels C–E. Seal 628 then encountered a second cyclone and its movement was briefly aligned with this feature as well in panel F. (H) Plot of estimated behavioral state (red line, scale on right y-axis) and 10 m height wind speed at 628's location (blue, scale on left y-axis) versus time for the overwintering period 2007–08. Wind estimates are obtained by interpolating National Centers for Environmental Prediction Reanalysis 2 (NCEP2) product to 628's estimated locations at each 6 h time point. Gray bars in alternating shading display the extent of time covered by panels B–G [file pone.0101268.s004.tif]

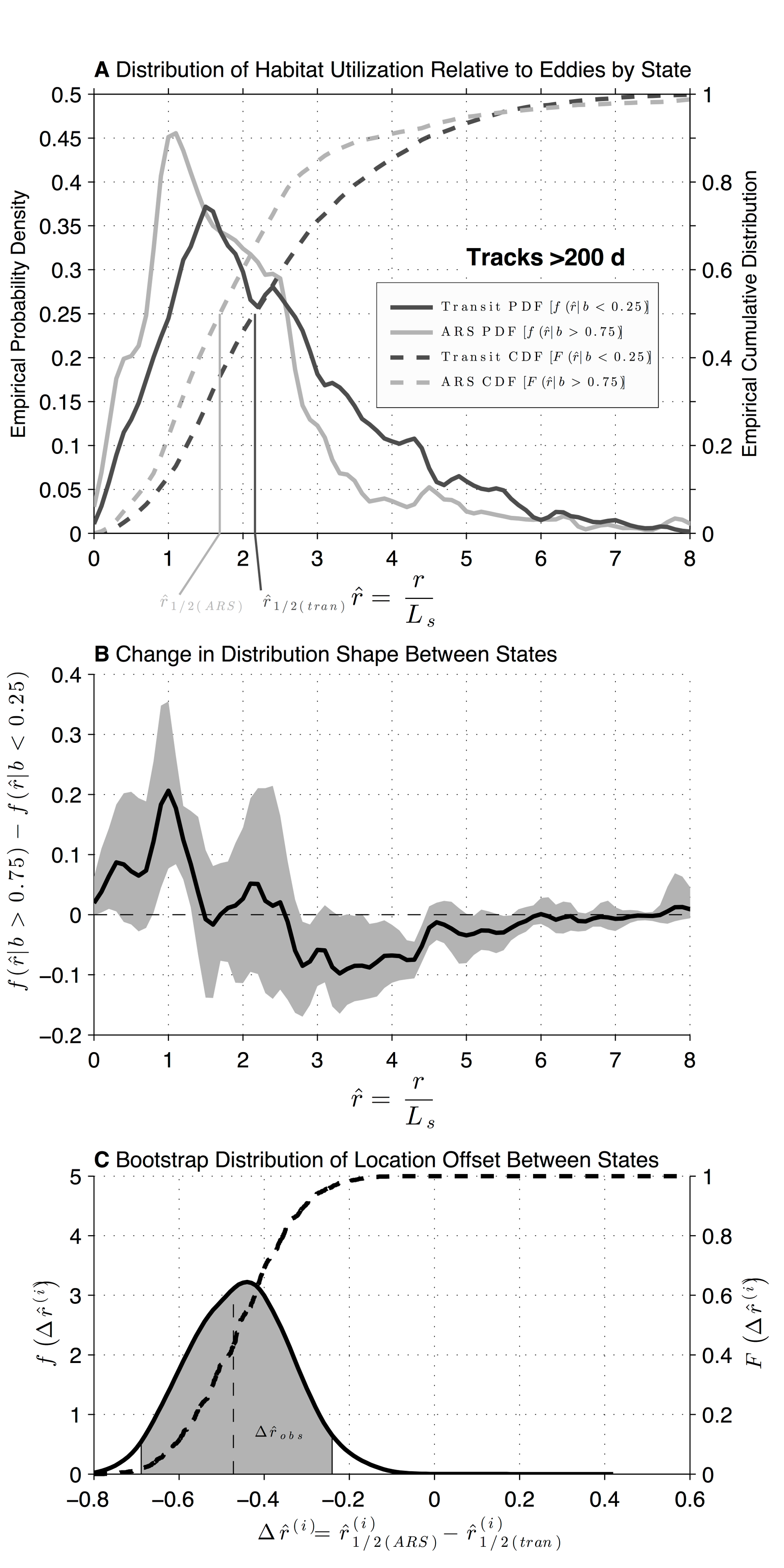

Supplement: Figure S5 — Adult female northern fur seal habitat utilization relative to eddy features, conditioned by estimated behavioral state (evaluated for tracks >200 d only). (A) One-dimensional radial probability density functions [PDFs, ] and cumulative distribution functions [CDFs, ] for habitat utilization relative to eddy features at two categories of behavioral state (transit = dark gray, area-restricted search [ARS] = light gray). “Transit” periods are those with ; “ARS” (area-restricted search) periods are those with . Solid lines show PDFs, dashed lines are CDFs (scale on right y-axis). Distributions are computed as a function of normalized distance to the nearest eddy center , defined as absolute distance to the nearest eddy divided by that eddy's radial length scale [63]. (B) Observed difference between search PDF and transit PDF (solid black line) with 95% confidence bounds computed using a bootstrap method (gray shading). Probability density functions show significant differences near () and (). (C) Bootstrap distribution of , the difference of median values of between ARS and transit states on bootstrap iteration . Solid black line shows the PDF of these values, thick dashed line shows CDF (scale at right). Thin vertical dashed line is the observed value of . The gray shaded area denotes the bias-corrected/accelerated 95% confidence interval on the observed value of . For the observed value of to be significantly different from zero using a two-tailed test, this confidence interval must not include zero. (TIF) [file pone.0101268.s005.tif]
