# Supplementary material for: Fortuitous Encounters between Seagliders and Adult Female Northern Fur Seals (Callorhinus ursinus) off the Washington (USA) Coast: Upper Ocean Variability and Links to Top Predator Behavior
Source: PLoS One. 2014 Aug 25;9(8):e101268. doi: 10.1371/journal.pone.0101268 (PMC4143212; doi:10.1371/journal.pone.0101268)
Supplement: Supporting Methodology S1 — Processing of fluorescence measurements from Seagliders. (DOCX) [file pone.0101268.s006.docx]

**Supporting Methodology S1. Processing of fluorescence measurements from Seagliders.**

Each Seaglider was equipped with a Western Environmental Technologies (WET) Labs ECO BB2F “puck”-style sensor, hereafter referred to as the “optical sensor,” that samples fluorescence on a single channel and optical backscatter on two channels. The sensor excites fluorescence at 470 nm wavelength and one channel measures its emission at 682 nm. The other channels measure total backscatter on two different wavelengths (470 nm and 700 nm). The optical sensor reports in units of digital instrument counts, and is calibrated such that net sensor counts are linearly proportional to *in situ* seawater fluorescence and optical scattering. Net sensor counts refer to counts above a background level that the sensor produces when not exposed to any source light [1]. This background level will be referred to hereafter as the “dark offset.” We report results from only fluorescence measurements in this manuscript.

The first step in processing of the fluorescence measurements is to determine the dark offset. Once this is accomplished, the sensor output can be converted to chlorophyll-*a* (Chl*a*) concentration (mg m^-3^) through the following [1]:

|  | $Chla=SF_{f}\times\left(C_{f}-DO_{f}\right)$ | (1) |
| --- | --- | --- |
|  |  |  |

In Equation 1, $C_{f}$ represents the instrument output in counts for the fluorescence channel, while $DO_{f}$ represents the dark offset. $SF_{f}$ represents the factory-derived scale factor that relates net counts above background on the fluorescence channel to Chl*a*.

To determine the dark offset for the fluorescence channel, two approaches may be used. First, WET Labs provides a factory calibration of each sensor that includes determination of the dark offset for the fluorescence and backscatter channels. The dark offsets are determined by measuring the counts produced when the sensor detector face is covered with dark tape and placed in a clear water bath [1]. A second method is to measure the dark offsets *in situ* after a Seaglider has been deployed, by measuring the counts produced by the sensor when sampling below the euphotic zone (away from any light sources) and in (assumedly) clear water. The first method has the advantage that the factory calibration is performed in water that is known to be clear, but at a temperature that may be different from that experienced when deployed on Seagliders. The second method gives a dark offset in field conditions, but may yield inaccurate values if the measurements used to determine the dark offset are not taken in water that is sufficiently free of organic matter and particles. We adopted the second approach, since [2] and [3] have demonstrated its validity for Seagliders operating off the Washington coast.

We collected a “clearest deep count” for each channel on each dive-climb cycle, defined as the minimum of values sampled below 145 m depth, where typically 150 m was the greatest depth at which fluorescence samples were collected on each profile. The dark offset was defined as the mean of the clearest deep counts over all offshore cycles, defined as those with a midpoint longitude west of 127ºW. This longitude boundary was chosen to ensure that the dark offset calculation did not include profiles that sampled intermediate nepheloid layers (deep layers of suspended bottom sediments; [4]), which were sometimes observed at 150 m depth within 10-20 km of the shelf break off the Cape Flattery line and Grays Harbor line. One sensor was fitted for only a single, brief deployment (23 December 2004 – 3 Jan 2005) and did not record any data west of 127ºW. We inspected the profiles from this deployment and found that no nepheloid layer was present west of 125.5ºW. For this sensor offshore casts were instead defined as those west of 125.5ºW; the deep counts from the 52 profiles collected in this region were comparably stable to all other sensors. The dark offsets were applied to the manufacturer’s formula (Eq. 1), along with factory-determined scale factors $SF_{f}$ for each sensor, to determine net counts above background and convert these to Chl*a* concentration.

Sackmann [3] and Perry et al. [2] reported good stability of dark water counts in all optical pucks through 2007 and we found similar results for the additional sensors deployed in 2008-09 that were not part of their analysis. Despite extensive laboratory calibration, absolute values of Chl*a* measured from Seagliders are roughly 300% too high when compared to shipboard and satellite measurements, though relative temporal and spatial structure measured from the sensors is robust [2,3,5]. The high values of Chl*a* that result from applying the factory calibrations are likely caused by phytoplankton species assemblages off the Washington coast that differ from the monoculture of phytoplankton that is used to determine $SF_{f}$ in the factory. Obtaining more accurate scaling factors for Seaglider fluorescence measurements would have required “ground-truthing” the Seaglider measurements, by collecting shipboard profiles of Chl*a* near Seagliders during the Washington coast time series.

Though performance of ECO sensor fluorometers of the type deployed on Seagliders in this study has been shown to be relatively insensitive to high concentrations of turbidity, sampling in large concentrations of chromorphic dissolved organic matter (CDOM) can induce a stepwise upwards shift in the fluorescence channel dark offset [5] and can also affect particulate scatter measurements by increasing along-path absorption. This is a concern when processing samples collected in river plumes that may contain large inputs of terrestrial CDOM. Seagliders did collect samples within portions of the Columbia River plume, but at large distances from the river mouth in what is known as the “far-field” region (salinities 30-32; [6]). Previous measurements of CDOM absorption coefficients in this region are close to open-ocean values [7] and their influence on ECO sensor fluorometer samples is likely small. Consistent with [2] we use exclusively nighttime values of Chl*a* in order to avoid near-surface fluorescence quenching, a phenomenon that biases near-surface fluorescence measurements collected during the daytime towards lower values [3,8].

We also extended the objective mapping procedure of [9], to map measurements of Chl*a* for each of the 124 cross-shore glider transects to a grid with regular horizontal and vertical spacing, just as with temperature, salinity, and density. Note that in glider data, adjacent profiles of Chl*a* may appear similar but with one profile offset in the vertical relative to the other – this is due to internal waves, which heave water parcels vertically over periods and spatial scales shorter than can be resolved by glider sampling [10]. Thus, when viewing the results of a single transect versus distance from shore and depth, spurious small-scale horizontal variability is apparent due to heaving. One purpose of mapping to a regular grid is to smooth the results of each crossing and remove this small-scale variability. When heaving occurs due to internal waves, water parcels are displaced from their vertical position, but remain on surfaces of neutral density, which in the top 1000 m are well approximated by potential density computed with a reference pressure of 0 dbar (the ocean surface). Thus, prior to mapping, for each Chl*a* sample we recorded the potential density value at which it was taken in the raw profiles. Potential density was then mapped to a regular grid following [9]. The mapping procedure filters small-scale horizontal variability in the depth of density surfaces. After regular grids of potential density were created, the depth of each raw Chl*a* sample was adjusted to match the depth of its density surface in the mapped data. These adjusted-depth Chl*a* samples were then log-normalized and mapped along depth surfaces using a 20 km decorrelation length scale. This scale is the largest observed horizontal spacing between adjacent night profiles.

**References**

1. (2011) ECO three-measurement sensor - triplet puck user's guide, revision H. Philomath OR: WET Labs. 9 p. Available: http://www.stccmop.org/files/tripletpuckh.pdf.

2. Perry M, Sackmann B, Eriksen C, Lee C (2008) Seaglider observations of blooms and subsurface chlorophyll maxima off the Washington coast. Limnol Oceanogr 53: 2169-2179.

3. Sackmann B (2007) Remote assessment of 4-D phytoplankton distributions off the Washington coast [Ph.D. Thesis]. Orono ME: University of Maine. 204 p.

4. Baker ET (1976) Temporal and spatial variability of the bottom nepheloid layer over a deep-sea fan. Mar Geol 21: 67-79.

5. Carroll M, Chigounis D, Gilbert S, Gundersen K, Hayashi K, et al. (2006) Performance verification statement for the WET Labs ECO FLNTUSB fluorometer. Solomons MD: Alliance for Coastal Technologies. 36 p.

6. Hickey BM, Kudela RM, Nash JD, Bruland KW, Peterson WT, et al. (2010) River influences on shelf ecosystems: introduction and synthesis. J Geophys Res-Oceans 115: C00B17.

7. Palacios SL, Peterson TD, Kudela RM (2009) Development of synthetic salinity from remote sensing for the Columbia River plume. J Geophys Res 114: C00B05.

8. Sackmann BS, Perry MJ, Eriksen CC (2008) Seaglider observations of variability in daytime fluorescence quenching of chlorophyll-*a* in northeastern Pacific coastal waters. Biogeosciences Discuss 5: 2839-2865.

9. Pelland NA, Eriksen CC, Lee CM (2013) Subthermocline eddies over the Washington continental slope as observed by Seagliders, 2003-09. J Phys Oceanogr 43: 2025-2053.

10. Rudnick DL, Cole ST (2011) On sampling the ocean using underwater gliders. J Geophys Res 116: C08010.
